# Peripheral brain-derived neurotrophic factor (BDNF) as a biomarker in bipolar disorder: a meta-analysis of 52 studies

**DOI:** 10.1186/s12916-015-0529-7

**Published:** 2015-11-30

**Authors:** Brisa S. Fernandes, Marc L. Molendijk, Cristiano A. Köhler, Jair C. Soares, Cláudio Manuel G. S. Leite, Rodrigo Machado-Vieira, Thamara L. Ribeiro, Jéssica C. Silva, Paulo M. G. Sales, João Quevedo, Viola Oertel-Knöchel, Eduard Vieta, Ana González-Pinto, Michael Berk, André F. Carvalho

**Affiliations:** Deakin University, IMPACT Strategic Research Centre, School of Medicine, Geelong, Australia; Laboratory of Calcium Binding Proteins in the Central Nervous System, Department of Biochemistry, Federal University of Rio Grande do Sul, Porto Alegre, Brazil; Institute of Psychology, Department of Clinical Psychology, Leiden University, Leiden, The Netherlands; Leiden Institute for Brain and Cognition, Leiden University Medical Center, Leiden, The Netherlands; Translational Psychiatry Research Group and Department of Clinical Medicine, Faculty of Medicine, Federal University of Ceará, Fortaleza, CE Brazil; Center of Excellence on Mood Disorders, Department of Psychiatry and Behavioral Sciences, Texas Health and Science University, Houston, TX USA; Laboratory of Neuroscience, LIM-27, Institute and Department of Psychiatry, University of Sao Paulo, Sao Paulo, Brazil; Center for Interdisciplinary Research on Applied Neurosciences (NAPNA), University of Sao Paulo, Sao Paulo, Brazil; Experimental Therapeutics and Pathophysiology Branch, National Institute of Mental Health, NIH, Bethesda, MD USA; Center for Translational Psychiatry, Department of Psychiatry and Behavioral Sciences, The University of Texas Medical School at Houston, Houston, TX USA; Neuroscience Graduate Program, Graduate School of Biomedical Sciences, The University of Texas Health Science Center at Houston, Houston, TX USA; Laboratory of Neurosciences, Graduate Program in Health Sciences, Health Sciences Unit, University of Southern Santa Catarina, Criciúma, SC Brazil; Laboratory for Neuroimaging, Department of Psychiatry, Psychosomatic Medicine and Psychotherapy, Goethe University, Frankfurt/Main, Germany; Bipolar Disorders Unit, Institute of Neuroscience, Hospital Clinic, University of Barcelona, IDIBAPS, CIBERSAM, Barcelona, Catalonia Spain; University of the Basque Country, Biomedical Research Center in Mental Health Net (CIBERSAM), Department of Neurosciences, University of the Basque Country, Leioa, Spain; Florey Institute of Neuroscience and Mental Health, Orygen, The National Centre of Excellence in Youth Mental Health and Orygen Youth Health Research Centre, Parkville, VIC Australia; Department of Psychiatry, University of Melbourne, Parkville, VIC Australia

**Keywords:** Biomarker, Bipolar disorder, Brain-derived neurotrophic factor, Meta-analysis

## Abstract

**Background:**

The neurotrophic hypothesis postulates that mood disorders such as bipolar disorder (BD) are associated with a lower expression of brain-derived neurotrophic factor (BDNF). However, its role in peripheral blood as a biomarker of disease activity and of stage for BD, transcending pathophysiology, is still disputed. In the last few years an increasing number of clinical studies assessing BDNF in serum and plasma have been published. Therefore, it is now possible to analyse the association between BDNF levels and the severity of affective symptoms in BD as well as the effects of acute drug treatment of mood episodes on BDNF levels.

**Methods:**

We conducted a systematic review and meta-analysis of all studies on serum and plasma BDNF levels in bipolar disorder.

**Results:**

Through a series of meta-analyses including a total of 52 studies with 6,481 participants, we show that, compared to healthy controls, peripheral BDNF levels are reduced to the same extent in manic (Hedges’ *g* = −0.57, *P* = 0.010) and depressive (Hedges’ *g* = −0.93, *P* = 0.001) episodes, while BDNF levels are not significantly altered in euthymia. In meta-regression analyses, BDNF levels additionally negatively correlate with the severity of both manic and depressive symptoms. We found no evidence for a significant impact of illness duration on BDNF levels. In addition, in plasma, but not serum, peripheral BDNF levels increase after the successful treatment of an acute mania episode, but not of a depressive one.

**Conclusions:**

In summary, our data suggest that peripheral BDNF levels, more clearly in plasma than in serum, is a potential biomarker of disease activity in BD, but not a biomarker of stage. We suggest that peripheral BDNF may, in future, be used as a part of a blood protein composite measure to assess disease activity in BD.

**Electronic supplementary material:**

The online version of this article (doi:10.1186/s12916-015-0529-7) contains supplementary material, which is available to authorized users.

## Background

The quest for useful biomarkers in bipolar disorder (BD) is gaining momentum. One of the most extensively investigated proteins in BD is brain-derived neurotrophic factor (BDNF). It was first isolated [1] after the serendipitous discovery of the nerve growth factor in 1952 [2]. The neurotrophic hypothesis was originally formulated in 1997 by Duman, Heninger, and Nestler [3], and characterizes major depressive disorder as being secondary to aberrant neurogenesis in brain regions that regulate emotion and memory, with aberrant neurogenesis associated with lower expression of BDNF. BDNF rapidly became a popular research topic, with the first study of BDNF levels in peripheral blood conducted in 2002 by Karege et al*.* [4].

Science historians have often noticed that, at any given time, scholars in a particular field tend to share basic assumptions about their subject [5]; this quickly turned out to be the case for the neurotrophic hypothesis, which, since its original formulation, was first expanded to include schizophrenia by Toyooka in 2002 [6]. BD followed, with Laske et al. [7], in 2005, first showing decreased serum BDNF levels in mania, and Palomino et al*.* [8], in 2006, showing that peripheral BDNF increased after treatment of acute mania.

Research on peripheral BDNF was originally driven by the aim of better understanding the pathophysiology of mood disorders; however, in the last few years, BDNF has been attracting attention as a potential biomarker capable of advancing the elusive field of personalised medicine in psychiatry [9–11]. BDNF was an obvious choice, since its levels in peripheral blood can be assessed easily and relatively non-invasively through venepuncture, and BDNF levels in serum and plasma are highly correlated with BDNF levels in the central nervous system, as BDNF freely crosses the blood–brain barrier [4, 12, 13]. In BD, several studies have been conducted with discrepant results. Most of these studies have suggested peripheral BDNF as a state-marker in BD, with decreased levels in mania and depression returning to normal in euthymia, and also being correlated with severity of mania and depression [14, 15]. Based on that, we earlier proposed peripheral BDNF as a potential biomarker of disease activity in BD [14, 16, 17], and presented preliminary data suggesting that BDNF could play a role as a biomarker capable of supporting the clinical diagnosis of BD. Bipolar disorder has been conceptualized as a neuroprogressive illness, in which recurring affective episodes may lead to cognitive deterioration and/or refractoriness, although it is acknowledged that some cognitive and functional problems are present to a lesser extent since the first bipolar episode [18, 19]. It has been suggested that BDNF levels may reflect neuroprogressive changes in BD, and thus may hold promise as a stage biomarker [11, 14, 20].

The inconsistent findings regarding peripheral BDNF levels in BD might be caused by heterogeneous patient populations or by small sample sizes lacking statistical power. Meta-analysis is a recognized technique used to resolve discrepancies between studies. It is a quantitative method that combines results from independent studies to increase statistical power in order to derive more solid conclusions [21, 22]. In addition, meta-regression may be used to evaluate confounders and discrepancies among different studies [23, 24]. At the moment, five meta-analyses have been conducted on the topic of peripheral BDNF levels in BD with conflicting results. The first three [14–16] showed decreased BDNF during acute mood states, and the last two showed BDNF levels decreased during depression but not in mania [25, 26]. The largest one to date included 35 studies; however, this is a rapidly evolving field, and now 52 studies are available. Thus, there is a sounder basis to definitively analyse the relationship between BDNF levels, mood states, severity of manic and depressive symptoms, and treatment response, including the pivotal role of peripheral BDNF levels as a biomarker in BD. Particularly, from a biomarker development perspective, it is important to analyse which compartment would be more adequate for BDNF measurement, plasma or serum, and if use of psychiatric drugs might influence BDNF as a biomarker.

Therefore, the aims of this large, collaborative meta-analysis were to verify the properties of peripheral BDNF levels as a biomarker of disease activity and of stage in BD. For this, we ascertained if peripheral BDNF levels are indeed decreased in BD across the different mood states and whether its levels are associated with severity of manic and depressive symptoms, thus being a state-marker and, as a result, behaving as a biomarker of disease activity. To determine if peripheral BDNF levels could be a stage biomarker in BD, we examined if its levels are associated with duration of illness. In addition, we aimed to assess whether BDNF levels change following pharmacological treatment of an acute mood episode. With this in mind, we performed a series of meta-analysis of all cross-sectional studies of peripheral BDNF levels in BD compared to healthy subjects, and also evaluated longitudinal studies on BDNF levels before and after prescription of psychiatric medication, exploring its relations to manic and depressive symptoms and response to treatment. Based on results from the previous meta-analyses, we expected high levels of between-study heterogeneity. Thus, we aimed to explore potential moderators of the differences in BDNF levels among individuals with BD compared to healthy controls, as well as longitudinally after pharmacological treatment. We also aimed to group results according to sample source (plasma, serum, or whole blood) to determine which one would be more appropriate for assessment of BDNF levels, and also to search for differences of efficiency in effect sizes and heterogeneity. All these aspirations are now possible due to the large amount of data currently available and will help to clarify the role of peripheral BDNF as a biomarker in BD.

## Methods

We performed four between-group meta-analyses of peripheral levels of BDNF in subjects with BD according to mood state: (1) in subjects with BD in mania compared to healthy controls; (2) in subjects with BD in depression compared to healthy controls; (3) in subjects with BD in a mixed episode compared to healthy controls; and (4) in subjects with BD in euthymia compared to healthy controls. We also conducted the following two within-group meta-analyses: (1) a meta-analysis of peripheral BDNF levels changes in participants with BD in a manic episode at baseline and after pharmacological treatment, and (2) a meta-analysis of peripheral BDNF levels in participants with BD in a depressive episode at baseline and after pharmacological treatment. The protocol developed for this meta-analytic review adhered to the recommendations of the Preferred Reporting Items for Systematic Reviews and Meta-Analyses (PRISMA) statement [27]. Each element of the literature search, selection of eligible studies and data extraction were performed by at least two authors (CMGSL, TLR, JCS, BSF, and MLM). Disagreements were resolved through consensus. Following harmonization of the data extraction process, three authors (AFC, BSF, and PMGS) checked the extracted data and corrected eventual inconsistencies.

### Search strategy

We conducted a systematic search of all potentially eligible references (including meeting abstracts) without language restrictions to avoid language publication bias, using PubMed/MEDLINE, EMBASE, and PsycINFO computerized databases. One article was published in mandarin and was translated by a native mandarin speaker. The search string used for the electronic database search was (Bipolar disorder OR mania OR bipolar depression) AND (BDNF OR brain-derived neurotrophic factor). The last search was performed in May 31st, 2015. This search strategy was augmented by tracking the citations of eligible articles in Google Scholar database to identify additional eligible references.

### Study selection

The inclusion criteria were (1) adult individuals with BD regardless of mood state meeting either International Classifications of Disease or Diagnostic and Statistical Manual for Mental Disorders diagnostic criteria; (2) pairwise comparison with a control group of healthy volunteers for the between-group meta-analyses, or longitudinal studies before and after drug treatment for an acute mood episode for the within-group meta-analyses; and (3) studies that measured peripheral BDNF levels in vivo. Exclusion criteria were (1) post mortem brain studies without information on BDNF levels in the periphery; (2) case reports; (3) genetic studies without information on BDNF levels in the periphery; (4) studies that included samples with mixed psychiatric diagnoses unless data for BD were reported separately or were obtained after contacting the authors; and (5) preclinical data. The authors consensually agreed on the final inclusion of references for this meta-analytic review.

### Data extraction

Two reviewers independently extracted data (sample size, mean and standard deviation) to prevent potential errors. All variables were extracted by diagnostic status (euthymic, manic, depressed, or mixed episode). We recorded age, sex (% female), and length of illness in years. We computed scores for manic as well as depressive symptoms as assessed through standard rating instruments, such as the Young Mania Rating Scale (YMRS) and the Hamilton Depression Rating Scale (HDRS) [28], respectively. We also extracted information regarding sampling (plasma, serum or whole blood), type of assay (radioimmunoassay or enzyme-linked immunosorbent assay), and manufacturer of the assay kit. Finally, information on age- and sex-matching of BD and control samples as well as the type of diagnostic interview was obtained. Subjects with BD were considered drug-free when they were off psychiatric medication for at least 2 weeks prior to venepuncture. Treatment response for the within-group meta-analyses was defined as an at least 50 % reduction in baseline HDRS or YMRS scores for a depressive or manic episode, respectively.

Discrepancies in data entry were double-checked by the reviewers with the original published data and a consensus was reached. Authors of meeting abstracts were contacted by e-mail on at least two different occasions requesting the provision of data. Furthermore, corresponding authors of included articles were contacted whenever necessary data were unavailable, and the required information was then requested. Whenever multiple reports pertained to the same participants, we included only the largest data set. In the within-group meta-analysis, we considered only the last post-intervention BDNF measurement. When data were available only in graphs, we extracted the data according to the procedure explained by Sistrom et al. [29].

### Publication bias

Studies reporting negative results (i.e. statistically non-significant results) are less likely to be published than studies with positive results [30, 31]. We estimated the likelihood of publication bias based on the following assumptions for the existence of small-study effects: (1) the effect size of the largest study is more conservative than the pooled effect size of the respective meta-analysis and (2) a *P* value of less than 0.1 in the Egger’s asymmetry test [32] as suggested by Belbasis et al. [33]. The trim-and-fill procedure, which is a validated model to estimate an effect size (ES) after bias has been taken into account, was employed when publication bias in the funnel plots was demonstrated. Finally, the file drawer statistic (i.e. the Fail-safe N test) was used to quantify the number of possible negative omitted studies necessary to turn the ES estimate non-significant.

### Statistical analysis

Eligible studies included different assay methods; thus, standardized mean difference estimates, using Hedges’ adjusted *g*, which provides a relatively unbiased ES adjusted for sample size, were used to estimate differences in peripheral BDNF levels of individuals with BD compared to healthy controls (in the between-group meta-analyses) and between baseline and post-treatment peripheral levels of BDNF (in the within-group meta-analyses). The 95 % confidence interval (95 % CI) of the ES was also computed. An ES of 0.2 was regarded as small, while an ES of 0.5 was considered moderate and an ES ≥0.8 was considered large.

Heterogeneity across studies was evaluated using the Cochran Q test, a weighed sum of the squares of the deviations in individual study ES estimates from the summary ES estimate, and a *P* value of <0.10 was considered significant (i.e. indicative of heterogeneity). We also calculated the *I*^*2*^ metric [24, 34, 35] as a measure of inconsistency across studies; *I*^2^ values >50 % were regarded as indicative of large heterogeneity, while *I*^2^ values >75 % were deemed as an evidence of very large heterogeneity. The *I*^2^ statistic should be interpreted as the proportion of total variance in study estimates that is due to heterogeneity. We also estimated the 95 % CI, which further accounts for between-study heterogeneity and evaluates the uncertainty of the effect that would be expected in new studies investigating the same association [36, 37]. Both measures of heterogeneity (i.e. the Cochran Q test and the *I*^2^ metric) have a limited power to detect heterogeneity unless very large data sets are available. Thus, we pooled ES estimates of individual studies using random effects, which allows population-level inferences and is more stringent than fixed-effects models. Random-effects models have the assumption that a genuine diversity across study results is present and incorporates a between-study variance into the calculations [38]. The level of significance for the effect estimates was set at α = 0.05.

Unrestricted maximum likelihood random-effects meta-regressions were performed with mean age of participants with BD, mean age of controls, sex (% female) of controls and patients, sample size (N), mean baseline YMRS or HDRS scores, follow-up duration (for within-group meta-analyses), and differences in baseline and post-treatment YMRS or HDRS scores (for within-group meta-analyses). We also performed the following subgroup analyses to search for potential sources of heterogeneity across studies: BDNF sampling (plasma versus serum versus whole blood), medication status (on and off psychiatric medication), and responders versus non-responders (for within-group meta-analyses). We also conducted sensitivity analyses to ascertain whether the summary ES estimates of our meta-analyses were strongly influenced by any single study.

Based on the summary ES estimates of each meta-analysis, we estimated the sample size that would be required for an individual study to detect this effect considering a power of 0.8 and an alpha level of 0.05. These analyses were performed using G*Power 3.1 software [39].

Thereafter, we performed a cumulative meta-analysis, which addresses the impact of new studies in prior pooled results. For this analysis, individual data sets were sorted in chronological order. The earliest available study was included in the analysis first. At each subsequent step of the cumulative meta-analysis, one more study was included in the analysis, and the summary ES and 95 % CI were recalculated. The ‘Proteus phenomenon’ refers to the situation in which the first published studies are often the most biased toward inflated effect sizes (i.e. the winner’s curse); subsequent replication studies tend to be less biased toward the extreme, often finding evidence of smaller effects or even contradicting the findings of initial studies. Thus, cumulative meta-analyses allow the appreciation of these phenomena.

We used a previously described test for excess significance [40]. Briefly, this test evaluates whether the number of studies with nominally significant results (i.e. with *P* <0.05) among those included in a meta-analysis is too large based on the power that these data sets have to detect effects at α = 0.05. The power estimate for each data set was calculated. The sum of the power estimates of each study provides the expected number of data sets with nominal statistical significance. As described elsewhere, the number of expected positive data sets can be compared with the observed number of statistically significant studies in a meta-analysis through a χ^2^-based test. The larger the difference between the observed number and the expected number, the higher the degree of excess significance bias.

We employed a Kruskal-Wallis test followed by a Bonferroni correction to perform a direct comparison of differences in the magnitude of the ESs of the different studies in the different mood states in the between-group meta-analyses. Results of this analysis are shown as medians and interquartile range of the ESs in the different mood states. All analyses were conducted with the Comprehensive Meta-analysis software version 2.0 (Borenstein, NH, USA) and/or the STATA version 13.0 software.

## Results

Overall, the six meta-analyses included 6,481 participants (3,339 cases with BD and 3,142 healthy controls). We identified 1,041 unique references through electronic database searches. Of those, 927 were excluded following title/abstract screening, leaving 114 studies for full-text review. One study for the between-group meta-analysis was excluded because it assessed BDNF levels in different mood states in rapid cycling subjects in a longitudinal manner; therefore, the BDNF assessed was subject to the risk of being influenced by the last episode [41]. Using the same logic, when a study reported BDNF levels in subjects with BD during mania or depression and healthy controls in the baseline, and in euthymia after treatment for an acute mood episode, we did not consider the BDNF values in euthymia for the between-group meta-analysis of euthymia compared to healthy controls.

Fifty-two studies fulfilled our inclusion criteria [7–9, 42–90], 44 for the between-group meta-analyses of BD versus controls in the different mood states [7–9, 42–50, 52, 53, 55, 57–64, 66, 68–77, 79–87, 90], providing data on 5,741 participants, of whom 2,599 were subjects with BD and 3,142 were healthy controls, and 18 studies for the within-group meta-analyses of BDNF changes after treatment following an index mood episode [8, 47, 51, 55–57, 59, 61, 64, 65, 67, 71, 78, 84, 87–90], comprising data on 740 participants. Some studies provided pairwise comparisons towards more than one meta-analysis. The PRISMA flowchart of study selection is depicted in Additional file 1: Figure S1, indicating the 70 studies excluded and the reasons for this (Additional file 2: Table S1).

The 44 studies included in the between-group meta-analyses were published from 2005 to 2015 and varied in sample size (from 26 to 493). The mean age varied from 21 to 65 years. Nineteen studies explored subjects with mania [7, 8, 43, 44, 46, 49, 50, 55, 57, 59–61, 64, 70, 76, 84, 85, 87, 90], 15 studies explored subjects with depression [9, 46, 49, 50, 52, 54, 57, 60, 68, 71, 72, 76, 81, 86, 90], 24 explored subjects in euthymia [42–49, 53, 58, 60, 62, 63, 66, 69, 72–74, 77, 79, 80, 82, 83, 86], and three examined subjects in a mixed episode [57, 72, 75]. In the studies that reported on manic subjects, the mean YMRS varied from 22 to 48. Mean HDRS scores varied from 18 to 28 in studies that reported on depressed subjects. Six studies in mania provided data regarding drug-free subjects [8, 50, 57, 61, 70, 87] and three regarding drug-free persons with depression [50, 68, 81]. All studies in euthymia only provided data for medicated subjects. Regarding the source of the peripheral BDNF levels, two studies analysed BDNF in whole blood [64, 87] and 15 in plasma [8, 43–45, 47, 48, 52, 61, 63, 68, 70, 77, 79, 81, 90]. All other studies considered BDNF in serum. In most of the studies, the control groups were matched by sex and age to the case groups.

The general demographic characteristics of the within-group meta-analyses were similar to the abovementioned for the between-group meta-analyses. Thirteen studies analysed changes in BDNF levels following treatment for an acute manic episode [8, 47, 51, 55, 57, 59, 61, 64, 65, 84, 87, 88, 90] and seven for an acute depressive episode [56, 57, 67, 71, 78, 89, 90]. The studies were published from 2006 to 2015 and also varied in sample size (from 6 to 198). The follow-up ranged from 4 to 52 weeks for an index manic episode, and from 1 to 16 weeks for an index depressive episode. In mania, the baseline mean YMRS scores ranged from 11 to 44, and in depression the mean baseline HDRS scores ranged from 18 to 24. All studies of an index acute manic episode achieved aggregate response, as defined by a decrease of at least 50 % in mean YMRS scores. The mean changes in the decreases in YMRS scores ranged from 51 % to 92 % of the baseline values. The psychiatric medications employed in the treatment of the manic episode in the studies considered included lithium, valproate, quetiapine, risperidone, and other atypical antipsychotics; most studies used a combination of these medications. Of the studies considering BDNF changes with treatment for an index acute depressive episode, three provided data for responders [57, 67, 78], as defined by a decrease of at least 50 % in mean HDRS scores, and four for non-responders [56, 78, 89, 90]. In general, the mean decreases in HDRS scores ranged from 10 % to 65 % of the baseline values. The psychiatric medications employed in the treatment of the depressive episode in the studies included valproate, quetiapine, risperidone, ketamine, a combination of atypical antipsychotics, and mifepristone.

The majority of the studies assessed BDNF levels using an ELISA kit. Detailed information regarding characteristics of the included studies in the between-group and within-group meta-analyses are provided in Additional file 2: Tables S2 and S3, respectively.

### Peripheral BDNF levels are decreased in BD in mania and depression in tandem with severity of symptomatology but not in euthymia

**Fig. 1:**
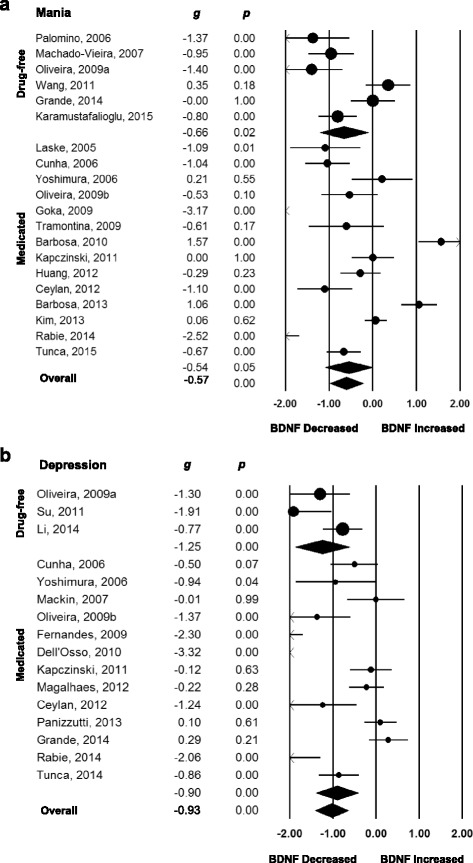
Forest plots of between-group meta-analyses measuring peripheral brain-derived neurotrophic factor (BDNF) levels in subjects with bipolar disorder compared to healthy controls, separated by mood state and medication status. (**a**) Mania, studies separated according use of medication. (**b**) Depression, studies separated according use of medication. The sizes of the circles are proportional to the sample size. Circles depict individual studies and diamonds depict the pooled effect sizes. Serum and plasma BDNF levels were decreased in subjects with bipolar disorder in mania and depression on and off psychiatric medication when compared to healthy controls

**Fig. 2:**
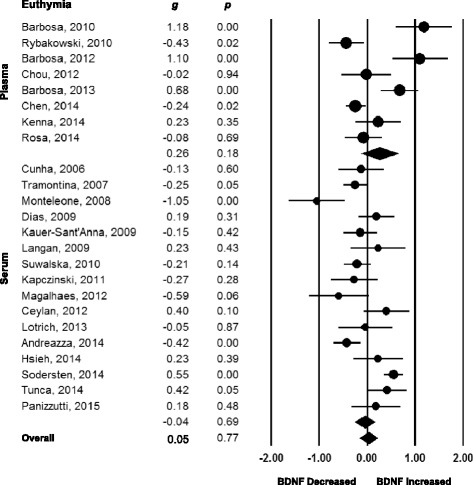
Forest plots of between-group meta-analysis measuring peripheral brain-derived neurotrophic factor (BDNF) levels in subjects with bipolar disorder in euthymia compared to healthy controls. All studies referred to persons on psychiatric medication. The sizes of the circles are proportional to the sample size. Circles depict individual studies and diamonds depict the pooled effect size. Serum and plasma BDNF levels were not altered in persons with bipolar disorder in euthymia when compared to healthy controls

Forty-four studies were included in the between-group meta-analyses of subjects with BD versus healthy controls in the different mood states [7–9, 42–50, 52, 53, 55, 57–64, 66, 68–77, 79–87, 90], providing data on 5,741 participants, of whom 2,599 were subjects with BD and 3,142 were healthy controls, which are summarized in Additional file 2: Table S2. Overall, random-effects between-group meta-analysis showed that peripheral BDNF levels were decreased in subjects with BD in mania with moderate effect sizes (*g* = −0.57, 95 % CI −0.99 to −0.14, *P* = 0.010, 19 between-group comparisons, n = 1,397) and decreased in depression with large effect sizes (*g* = −0.93, 95 % CI −1.37 to −0.50, *P* = 0.001, 15 between-group comparisons, n = 1,074) when compared to healthy controls. In contrast, there were no changes in peripheral BDNF levels in euthymia (*g* = 0.05, 95 % CI −0.13 to 0.24, *P* = 0.569, 24 between-group comparisons, n = 3,057; Table 1, Figs. 1 and 2).

**Table 1:** Statistics on between-group meta-analyses regarding peripheral brain-derived neurotrophic factor levels in bipolar disorder

Between-group	N of pairwise	Number of subjects		Meta-Analysis				Heterogeneity		
		BD	Controls	Hedges’ *g*	95 % CI		*P* value	*I* ^2^	Q	*P* value
Mania										
Mania vs. HC – all*	19	605	792	−0.57	−0.99	−0.14	0.010	92.07	239.69	0.001
Mania vs. HC – drug-naïve or free*	6	175	186	−0.66	−1.14	−0.10	0.020	79.43	29.17	0.001
Mania vs. HC – medicated	14	430	628	−0.54	−1.09	0.00	0.051	92.71	178.41	0.001
Mania vs. HC – serum*	12	288	512	−0.97	−1.41	−0.53	0.001	86.45	81.12	0.001
Mania vs. HC – plasma	6	206	188	−0.03	−0.98	0.95	0.951	94.73	94.94	0.001
Mania vs. HC – plasma Barbosa excluded*	4	124	92	−0.72	−1.27	−0.17	0.001	70.06	10.02	0.018
Mania vs. HC – whole-blood	2	144	153	0.12	−0.11	0.35	0.296	N/A	N/A	N/A
Mania vs. HC – age-/sex-matched	14	488	551	−0.46	−0.99	0.07	0.073	93.09	202.40	0.001
Mania vs. HC – not age-/sex-matched*	5	117	241	−0.84	−1.51	−0.18	0.042	86.17	28.90	0.001
Depression										
Depression vs. HC – all*	15	352	722	−0.93	−1.37	−0.50	0.001	87.88	107.27	0.001
Depression vs. HC – drug-naïve or free*	3	47	210	−1.24	−1.88	−0.61	0.001	64.24	5.59	0.061
Depression vs. HC – medicated*	13	305	534	−0.90	−1.59	−0.55	0.001	88.40	95.51	0.001
Depression vs. HC – serum*	12	305	519	−0.72	−1.17	−0.27	0.002	87.53	114.67	0.001
Depression vs. HC – plasma*	3	47	203	−1.94	−3.38	−0.49	0.009	90.25	20.51	0.001
Depression vs. HC – age-/sex-matched*	10	245	501	−0.81	−1.29	−0.35	0.001	82.26	45.11	0.001
Depression vs. HC – not age-/sex-matched*	5	107	221	−1.23	−2.30	−0.17	0.023	93.18	58.73	0.001
Depression vs. HC – only BD type I*	12	219	369	−1.27	−1.80	−0.72	0.001	86.05	71.70	0.001
Euthymia										
Euthymia vs. HC – all	24	1598	1459	0.05	−0.13	0.24	0.569	81.19	122.29	0.001
Euthymia vs. HC – serum	16	980	1022	−0.04	−0.16	0.17	0.689	86.67	69.66	0.001
Euthymia vs. HC – plasma	8	618	437	0.26	−0.12	0.65	0.626	86.67	52.51	0.001
Euthymia vs. HC – age-/sex-matched	16	658	733	0.02	−0.29	0.33	0.894	87.41	119.19	0.001
Euthymia vs. HC – not age-/sex-matched	7	919	700	−0.06	−0.40	0.26	0.702	88.18	50.75	0.001
Euthymia vs. HC – only BD type I	16	631	757	0.01	−0.30	0.32	0.319	87.00	115.43	0.001
Euthymia vs. HC – only BD type II	2	31	48	−0.36	−1.46	0.73	0.729	81.66	5.45	0.001
Mixed State										
Mixed state vs. HC – all	3	44	169	0.09	−0.57	0.75	0.787	69.14	6.48	0.039
Moderators between-group	N of pairwise	Number of subjects		Meta-regression				Meta-regression		
		BD	Controls	Slope	95 % CI		*P* value	Intercept	Z	*P* value
Mania										
Age of BD patients – all*	19	605	792	0.07	0.02	0.13	0.005	−3.41	−3.27	0.001
Age of BD patients – Barbosa excluded	17	523	696	0.03	−0.03	0.10	0.305	−1.93	−1.54	0.123
Age of controls – all	19	605	792	0.04	−0.01	0.01	0.165	−2.14	−1.85	0.064
Age difference_(BD-HC)_ – all*	19	605	792	0.18	0.05	0.31	0.004	−0.61	−3.15	0.001
Age difference_(BD-HC)_ – Barbosa excluded	17	523	696	0.08	−0.07	0.24	0.295	−0.74	−3.86	0.001
%Females (Patients)– all	17	579	760	0.01	−0.01	0.04	0.288	−1.33	−1.76	0.076
%Females (HC) – all	17	579	760	0.00	−0.02	0.02	0.811	−0.75	−0.96	0.332
Difference in %females _(BD-HC)_ – all	17	579	760	0.02	−0.01	0.05	0.250	−0.54	−2.25	0.023
Mean illness duration (years) – all	13	481	568	0.10	−0.01	0.21	0.070	−1.60	−2.27	0.023
Sample size– all	19	605	792	0.00	−0.00	0.01	0.126	−1.09	−2.68	0.009
YMRS scores – all*	17	583	750	−0.09	−0.15	−0.03	0.004	2.44	2.33	0.019
YMRS scores – plasma*	5	192	176	−0.09	−0.16	−0.01	0.017	2.96	2.47	0.013
YMRS scores – serum	10	247	421	−0.07	−0.15	0.00	0.052	1.63	1.18	0.235
Depression										
Age of BD patients – all	14	346	702	0.01	−0.04	0.06	0.681	−1.41	−1.21	0.225
Age of controls – all	14	346	702	0.00	−0.06	0.07	0.996	−1.00	−0.76	0.445
Age difference_(BD-HC)_ – all	14	346	702	0.08	−0.07	0.24	0.262	−1.22	−3.99	0.001
%Females (HC) – all	14	346	702	−0.00	−0.02	0.01	0.850	−1.03	−1.62	0.104
%Females (Patients) – all	14	346	702	0.00	−0.01	0.01	0.782	−1.28	−2.30	0.020
Difference in %females _(BD-HC)_ – all	14	346	702	0.02	−0.01	0.05	0.193	−0.95	−3.68	0.001
Mean illness duration (years) – all*	5	103	359	−0.14	−0.20	−0.07	0.001	−2.47	3.44	0.001
Sample size – all	15	352	722	0.00	−0.00	0.01	0.070	−1.60	−3.74	0.001
Year of publication – all	15	352	722	0.03	−0.14	0.19	0.726	−61.80	−0.35	0.722
HDRS scores – all*	13	311	609	−0.23	−042	−0.04	0.018	4.40	1.93	0.053
HDRS scores – serum	11	274	427	−0.12	−0.35	0.09	0.265	1.70	0.60	0.542
Euthymia										
Age of BD patients – all	24	1598	1459	−0.00	−0.02	0.02	0.950	0.11	0.23	0.831
Age of controls – all	24	1598	1459	−0.00	−0.02	0.01	0.802	0.16	0.36	0.712
Age difference_(BD-HC)_ – all	24	1598	1459	0.02	−0.06	0.11	0.537	0.04	0.41	0.676
%Females (HC) – all	23	1425	1197	0.00	−0.01	0.01	0.878	−0.03	−0.05	0.995
%Females (Patients) – all	23	1425	1197	0.00	−0.01	0.02	0.690	−0.26	−0.40	0.684
Difference in %females _(BD-HC)_ – all	23	1425	1197	0.01	−0.02	0.03	0.555	0.06	0.68	0.495
Number of mood episodes – all	5	206	220	0.01	−0.05	0.06	0.833	−0.24	−0.58	0.557
Illness duration (years) – all	13	831	696	0.01	−0.02	0.05	0.577	0.01	0.01	0.992
Illness duration (years) – excluding Barbosa 2010, 2012, 2013	10	739	575	−0.01	−0.04	0.02	0.496	0.13	0.48	0.630
Sample size – all	24	1598	1459	−0.00	−0.01	0.01	0.530	0.12	0.85	0.390

HDRS, Hamilton Depression Rating Scale; YMRS, Young Mania Rating Scale; CI, Confidence interval; BD, Bipolar disorder; HC, healthy controls; N/A, not applicable

*p<0.05

When we carried out sub-group analyses according to the use of psychiatric medication, we verified that BDNF levels remained decreased with a moderate effect size in subjects in mania when the subjects were drug-free (*g* = −0.66, 95 % CI −1.14 to −0.10, *P* = 0.020, 6 between-group comparisons, n = 361) but not when the subjects were on psychiatric medication (*g* = −0.54, 95 % CI −1.09 to −0.00, *P* = 0.051, 14 between-group comparisons, n = 1,058). However, in the last sub-group, the 95 % CI was large, and the result was verging on significant (Table 1, Fig. 1a). Similarly, BDNF levels remained decreased with a large effect size in individuals in depression irrespective of them being drug-free (*g* = −1.24, 95 % CI −1.88 to −0.61, *P* = 0.001, 3 between-group comparisons, n = 257) or on psychiatric medication (*g* = −0.90, 95 % CI −1.59 to −0.55, *P* = 0.001, 13 between-group comparisons, n = 839; Table 1, Fig. 1b). Peripheral BDNF levels were not significantly altered in BD participants in a bipolar mixed episode compared to healthy controls (*g* = 0.09, 95 % CI −0.57 to 0.75, *P* = 0.787, 3 between-group comparisons, n = 213); however, only three studies [57, 72, 75] were included in this sub-group and the 95 % CI was extremely large and, consequently, this analysis is likely to be underpowered (Table 1, Additional file 1: Figure S2).

**Fig. 3:**
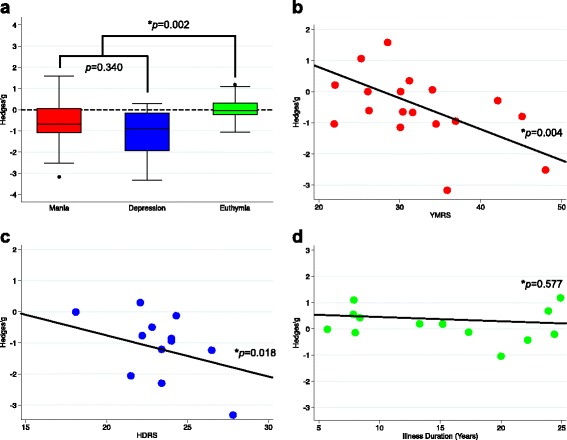
(**a**) Direct comparison of the effect sizes of the different studies in mania, depression, and euthymia. Peripheral brain-derived neurotrophic factor (BDNF) levels were equally decreased in mania and depression (*P* = 0.340), and both manic and depressive states presented BDNF levels decreased when compared to euthymic state (*P* = 0.002). (**b**) Meta-regression of the effect sizes of peripheral BDNF levels against severity of mania as assessed by the Young Mania Rating Scale (YMRS) scores, showing more accentuated decreases in BDNF levels with increase in YMRS scores (*P* = 0.004). (**c**) Meta-regression of the effect sizes of peripheral BDNF levels against severity of depression as assessed by the Hamilton Depression Rating Scale (HDRS) scores, showing more accentuated decreases in BDNF levels with increase in HDRS scores (*P* = 0.018). (**d**) Meta-regression of the effect sizes of peripheral BDNF levels against duration of illness in years in euthymic subjects, showing no association between BDNF levels and duration of bipolar illness in years during euthymia (*P* = 0.577)

We set to verify if there were differences in the extent of the decrease of peripheral BDNF levels according to mood states in order to assess the properties of peripheral BDNF levels as a possible state biomarker of disease activity in BD. For this, we performed a direct comparison of the ESs of the different studies in mania, depression, and euthymia. In general, the median and interquartile range of the ESs were different across the mood spectrum (−0.67, −1.09 to 0.06 in mania; −0.86, −1.91 to −0.13 in depression; −0.03, −0.24 to 0.31 in euthymia; *P* = 0.002, 58 comparisons, n = 5,528). Peripheral BDNF levels were equally decreased in mania and depression (*P* = 0.340, 34 comparisons, n = 2,471), and both manic and depressive states presented BDNF levels to be decreased when compared to the euthymic state (*P* = 0.014, 43 comparisons, n = 4,454 for mania vs. euthymia; *P* = 0.001, 39 comparisons, n = 4,131 for depression vs. euthymia; Bonferroni correction for multiple comparisons applied). Although the difference between those in acute manic and depressive episodes was statistically significant when compared to those in euthymia, the variability was large, and there was considerable overlap between the values of BDNF levels found in mania and depression with those in euthymia (Fig. 3a).

In univariable meta-regression models, we found a negative relationship between BDNF levels and severity of manic symptoms according to YMRS scores in persons with acute mania, and of depressive symptoms according to HDRS scores in persons with a current depressive episode, indicating that the greater the severity of manic and depressive symptoms, the greater the decrease in BDNF levels (Table 1, Fig. 3b, c). When a separate analysis according to the source of peripheral BDNF was performed, we verified that, in mania, the severity of YMRS scores was negatively related to the magnitude of the ES in plasma, but not in serum. In depression, when we sub-grouped the meta-regressions according to source, the previous significant effect was lost in serum, and, since only two studies provided data on HDRS in plasma of depressed individuals, it was not possible to perform a meta-regression in this scenario. However, this may suggest that most of the significance of the association of HDRS scores with BDNF levels was due to the use of plasma in the same line as that in mania (Table 1).

Another important predetermined moderator was length of illness, defined as the difference between age at the moment of the blood draw and age of the occurrence of the first episode in years. In univariable meta-analysis, we found a negative relationship between BDNF levels and length of illness in years during a depressive episode, but not during a manic episode. Since most of the studies did not provide data on length of the current index mood episode, it is not possible to analyse if the significant result found in depression was due to longer depressive episodes and a consequent decrease in time spent in remission. During euthymia, we found no relationship between length of illness and BDNF levels, even when three studies conducted by Barbosa et al*.* [43–45], which could be considered potentially outliers, were excluded (Fig. 3d, Table 1).

### BDNF levels increase after successful treatment of mania

**Fig. 4:**
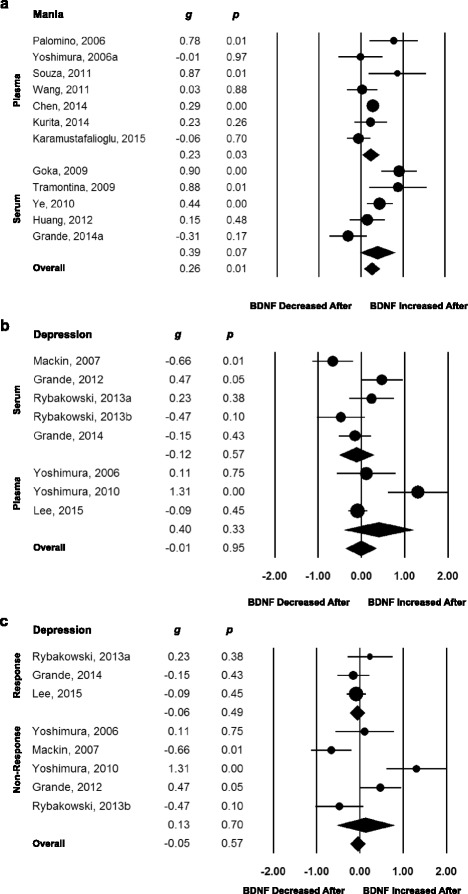
Forest plots of within-group meta-analyses measuring peripheral brain-derived neurotrophic factor (BDNF) levels in subjects with bipolar disorder before and after treatment for an index acute manic or depressive mood episode. (**a**) Mania studies grouped according the source of the blood sample. All studies presented response to the manic episode after pharmacological treatment, as defined as a decrease of at least 50 % on the Young Mania Rating Scale scores. Plasma BDNF levels increased after successful treatment of an index manic episode. Serum BDNF levels remained unchanged. (**b**) Depression studies grouped according the source of the blood sample. Serum and plasma BDNF levels remained unchanged after treatment of an index depressive episode. (**c**) Depression studies grouped according response or non-response to pharmacological treatment of the acute depressive episode, defined as a decrease of at least 50 % on the Hamilton Depression Rating Scale scores. Serum and plasma BDNF levels remained unchanged after treatment of an index depressive episode regardless of response or non-response to treatment. The sizes of the circles are proportional to the sample size. Circles depict individual studies and diamonds depict the pooled effect sizes

In order to verify if pharmacological treatment of an index mood episode induced changes in peripheral BDNF levels, we conducted two within-group meta-analyses of longitudinal studies, one of BDNF changes before and after treatment of a manic episode, and one of BDNF levels before and after treatment of a depressive episode. In total, 18 studies were included [8, 47, 51, 55–57, 59, 61, 64, 65, 67, 71, 78, 84, 87–90]. Thirteen studies referred to pharmacological treatment of a manic episode [8, 47, 51, 55, 57, 59, 61, 64, 65, 84, 87, 88, 90], comprising 556 subjects. In all studies, response, defined as a decrease in at least 50 % of the baseline mean YMRS scores, was achieved in all studies, and full remission, defined as mean YMRS scores of less than 7 at follow-up, was achieved in all but three [51, 65, 88]. Overall, peripheral BDNF levels showed a small increase after treatment of a manic episode (*g* = 0.26, 95 % CI 0.09 to 0. 54, *P* = 0.003, 13 between-group comparisons, n = 556). When performing sub-group analyses according to source, we verified that BDNF levels increased in plasma (*g* = 0.23, 95 % CI 0.02 to 0.44, *P* = 0.028, 7 between-group comparisons, n = 332) but not in serum (*g* = 0.39, 95 % CI −0.03 to 0.81, *P* = 0.065, 5 between-group comparisons, n = 122; Fig. 4a, Table 2). One study assessed BDNF in whole blood [64] and found a non-significant result. In univariable meta-regressions, we found no influence of duration of follow-up in weeks or of improvement of YMRS scores in the ES of BDNF levels before and after treatment (Table 2).

**Table 2:** Statistics on within-group meta-analyses regarding peripheral brain-derived neurotrophic factor levels in bipolar disorder

Within-Group	N of pairwise	Number of subjects	Meta-analysis				Heterogeneity		
			Hedges’ *g*	95 % CI		*P* value	*I* ^2^	Q	*P* value
Mania									
Mania– all*	13	556	0.26	0.09	0.45	0.003	69.69	39.60	0.001
Serum and plasma*	12	454	0.30	0.08	0.44	0.005	78.48	18.59	0.001
Serum	5	122	0.39	−0.03	0.81	0.065	78.48	18.59	0.001
Plasma*	7	332	0.23	0.02	0.44	0.028	55.20	13.39	0.037
Whole blood	1	102	0.01	−0.19	0.20	0.941	N/A	N/A	N/A
Depression									
Depression – all	8	184	0.05	−0.28	0.38	0.747	76.51	29.79	0.001
Depression – except Mackin 2007 [71]	7	164	0.15	−0.18	0.49	0.364	72.84	22.09	0.001
Serum	5	88	−0.12	−0.52	0.29	0.563	72.15	14.36	0.006
Plasma	3	96	0.40	−0.40	1.21	0.329	85.93	14.22	0.001
Response	3	115	−0.06	−0.24	0.18	0.492	N/A	N/A	N/A
Non-response	5	69	0.13	−0.53	0.79	0.702	85.69	1.51	0.001
Moderators within-group	N of pairwise	Number of subjects	Meta-regression				Meta-regression		
			Slope	95 % CI		*P* value	Intercept	Z	*P* value
Mania									
Age of BD Patients – all	9	456	−0.01	−0.04	0.02	0.398	0.80	1.39	0.165
% Females – all	13	556	0.01	−0.03	0.02	0.478	0.07	0.27	0.782
Baseline YMRS scores	12	542	0.01	−0.01	0.02	0.930	0.20	0.63	0.522
Difference in YMRS_(After – Before)_	12	542	−0.01	−0.02	0.02	0.298	0.16	1.42	0.155
% Change in YMRS Scores	12	542	−0.01	−0.02	0.01	0.213	0.77	1.73	0.083
Follow-up duration (weeks)	13	556	0.01	−0.01	0.03	0.396	0.19	1.63	0.101
Year of publication	13	556	−0.07	−0.12	−0.01	0.011	141.63	2.53	0.011
Depression									
Age of BD patients – all	6	164	−0.01	−0.05	0.02	0.393	0.50	0.69	0.489
% Females – all*	6	164	0.01	0.00	0.02	0.042	−0.54	−2.29	0.021
% Females – excluding Mackin 2007 [71]	5	144	0.01	−0.01	0.01	0.555	−0.20	−0.68	0.490
Baseline HDRS scores	8	184	0.02	−0.18	0.22	0.823	−0.42	−0.19	0.845
Difference in HDRS_(After – Before)_	7	164	−0.02	−0.09	0.06	0.658	−0.01	−0.01	0.988
% Change in HDRS scores	7	164	0.01	−0.01	0.02	0.566	−0.05	−0.13	0.892
Follow-up duration (weeks)	8	184	0.01	−0.05	0.07	0.673	−0.04	−0.12	0.899
Year of publication	8	184	0.01	−0.11	0.11	0.987	−1.76	−0.01	0.988

HDRS, Hamilton Depression Rating Scale; YMRS, Young Mania Rating Scale; CI, Confidence interval; BD, Bipolar disorder; N/A, Not applicable

*p<0.05

Seven studies [56, 57, 67, 71, 78, 89, 90] comprising 184 subjects investigated changes in peripheral BDNF levels before and after treatment for a current depressive episode. Overall, there were no changes in peripheral BDNF levels before and after pharmacological treatment (*g* = 0.06, 95 % CI −0.29 to 0.41, *P* = 0.747, 8 between-group comparisons, n = 184). When we excluded the study of Mackin et al. [71], which employed mifepristone as the pharmacological treatment for depression, the results remained unchanged (Table 1). In addition, the results remained non-significant when we sub-grouped the studies conducted using serum or plasma separately (Fig. 4b), and according to presence or absence of response to treatment of the depressive episode as assessed using HDRS scores (Fig. 4c). Again, the meta-regressions showed no effect of length of the treatment employed in weeks and changes of HDRS scores on BDNF levels (Table 2).

### Investigation of heterogeneity: sub-groups and meta-regressions analyses

We performed further sub-group and meta-regression analyses to explore potential sources of between-study heterogeneity in both between- and within-group meta-analyses. In the between-group meta-analyses, the results remained significant in depression when we assessed peripheral BDNF levels according to source (i.e. serum or plasma), and also remained non-significant in euthymia regardless of the source. However, in mania, the results remained significant in serum (*g* = −0.97, 95 % CI −1.41 to −0. 53, *P* = 0.001, 12 between-group comparisons, n = 800) but not in plasma (*g* = −0.03, 95 % CI −0.98 to 0.95, *P* = 0.951, 6 between-group comparisons, n = 394). However, in plasma, only six studies were available, and the 95 % CI were extremely large. When we analysed studies in mania using plasma excluding the studies of Barbosa et al. [43, 44], which included chronic and heavily medicated subjects and were possible outliers, the pooled ES yielded a significant result (*g* = −0.72, 95 % CI −1.27 to −0.17, *P* = 0.001, 4 between-group comparisons, n = 116).

Again, when we performed sub-group analyses considering whether the control group was matched to the bipolar group, we found no differences between matched and non-matched studies in depression and euthymia. However, in mania, BDNF levels only remained significant in the non-matched subgroup, although again the 95 % CI was very large (*g* = −0.46, 95 % CI −0.99 to 0.07, *P* = 0.073, 14 between-group comparisons, n = 1,039 in the matched group; *g* = −0.84, 95 % CI −1.51 to −0.18, *P* = 0.042, 14 between-group comparisons, n = 358 in the not-matched group). In addition, we wanted to verify if there were differences in peripheral BDNF levels in those with types I or II BD. In euthymia, the differences in BDNF levels between persons with BD and healthy controls remained non-significant regardless of whether the subjects had type I or II BD. In depression, the results remained significant only when depressed subjects with type I BD were included (*g* = −1.27, 95 % CI −1.80 to −0.72, *P* = 0.001, 12 between-group comparisons, n = 588). It was not possible to carry out an analysis considering only subjects in a depressive state with type II BD. Heterogeneity remained very high in all sub-group scenarios (Table 1).

In the meta-regressions of the between-group meta-analyses, sex, year of publication, and sample size did not seem to contribute to heterogeneity in between-group meta-analyses comparing peripheral BDNF levels of participants in mania, depression, or euthymia compared to their respective healthy controls (Table 1). The mean age of manic subjects, and most notably the mean difference in age between manic subjects and healthy controls included in a particular study appeared to contribute to heterogeneity in the between-group meta-analysis of mania versus healthy controls. Mean age did not seem to contribute to heterogeneity in between-group meta-analyses comparing peripheral BDNF levels of participants in depression or euthymia compared to their respective healthy controls (Table 1).

In the within-group meta-analysis involving participants with a manic or depressive state, sub-group analyses or meta-regression could not explain the high heterogeneity that was found. In the within-group meta-analysis of mania, when we performed sub-group analyses according to the source of BDNF, we verified that studies that assessed BDNF in plasma showed lower heterogeneity than those in serum (*I*^2^, 55.2 in plasma and 78.5 in serum; Table 2). When we assessed the effect of moderators using meta-regressions, we verified that heterogeneity in mania could not be explained by percentage of female participants, mean age, mean length of illness, follow-up time, sample size, or differences in YMRS scores between baseline and post-intervention (Table 2). Year of publication was the only moderator that achieved statistical significance in the treatment of a manic episode, showing that the magnitude of the ES decreased with time (Table 2). In depression, higher percentages of female participants were associated with a small increase in BDNF levels (Table 2).

### Sensitivity analyses and cumulative meta-analyses

We conducted a sensitivity analysis in all meta-analyses excluding studies one at a time to determine the robustness of the analyses and to verify if a particular study was responsible for the high heterogeneity or significance of the pooled ES estimate. No single study thoroughly explained the heterogeneity, and the results remained significant in all cross-sectional meta-analysis of mania, depression, euthymia, and all longitudinal meta-analysis of mania and depression (Additional file 1: Figures S3 to S7).

We sought to determine the stability of ES estimates over time through cumulative meta-analyses. For the between-group meta-analysis that included participants with mania versus healthy controls, the ES estimates were larger from 2005 to 2013. Thereafter, the ES reached stability (Additional file 1: Figure S8). An opposite behaviour was found for the between-group meta-analysis of depression versus healthy controls, where the magnitude of the pooled ES increased from 2006 to 2010, and thereafter started to decrease again, reaching stability in 2013 (Additional file 1: Figure S9). For the between-group meta-analysis of euthymic BD versus healthy controls, a small dispersion in the ES from 2006 to 2011 was verified, while the ES of individual studies reached stability since then (Additional file 1: Figure S10). When considering the within-group meta-analyses of both depression and mania, ES estimates reached stability since 2013, becoming smaller and tending towards zero (Additional file 1: Figures S11 and S12).

### Bias assessment and power

The evidence suggests the existence of small study effects, which is indicative of publication bias, in the between-group meta-analyses comparing depression or mania to healthy controls (Additional file 2: Tables S4 and S5), whereas evidence for publication bias was not observed in the other meta-analyses. Funnel plots are depicted in Additional file 1: Figures S13 to S17. The trim-and-fill procedure was thus performed in the between-group meta-analyses of depression or mania compared to healthy controls. However, no additional studies were imputed in these meta-analyses (Additional file 1: Figures S13 and S14). In addition, the fail-safe N statistic revealed that 253 and 409 additional studies, respectively, would be required to turn the ESs of the between-group meta-analyses of mania and bipolar depression non-significant.

An excess of significance bias was assessed in all meta-analyses through the Ioannidis test [40], considering the ES of the largest study as the true ES of each meta-analysis. An excess of significance bias was observed for between-group meta-analyses that included participants with euthymic BD and mania compared to healthy controls, and for the within-group meta-analysis of depression (Additional file 2: Table S5). No evidence for this type of bias was found in the between-group meta-analysis of depression versus healthy controls, or in the between-group meta-analysis of serum BDNF differences in manic subjects versus controls. There was also no evidence of excess significance in the within-group meta-analysis that included participants with mania (Additional file 2: Table S5).

Between-study heterogeneity was large or very large for all meta-analyses, and the 95 % prediction intervals of all meta-analyses included the null value, meaning that if a new future study were conducted on the subject, it could generate a non-significant result (Additional file 2: Table S5).

In addition, we estimated the sample size required to identify an ES of 0.26 (we assumed that the true real ES would equal that of the pooled ES of our meta-analysis) taking in account a power of 0.80 and an alpha level of 0.05 (two-tailed paired *t*-test) following treatment of acute mania. These calculations estimated that a sample size of 119 manic participants would be necessary. Eligible study sample sizes ranged from 10 to 116 participants, and thus were likely underpowered to detect differences in BDNF under these assumptions. In the between-group meta-analyses, 11 participants were necessary to reliably detect the observed ES in depression, while 28 participants were needed to detect the observed difference in mania.

## Discussion

Our six meta-analyses of peripheral BDNF levels in BD included a total of 52 cross-sectional or longitudinal studies comprising 6,481 participants. BDNF levels were moderately decreased in persons with BD during mania and largely decreased during depression. The extent of the decrease in peripheral BDNF levels paralleled the severity of manic and depressive episodes [14]. There were no alterations in peripheral BDNF levels in euthymia or in mixed states compared to healthy controls. Notably, there was no association between BDNF levels and duration of illness in euthymia, suggesting it is not a useful biomarker of stage. Importantly, in direct comparisons across distinct mood states, we demonstrated that the magnitude of the reduction in peripheral BDNF levels is comparable in mania and depression, and that both are equally reduced when compared to euthymia, suggesting peripheral BDNF levels as a biomarker capable of addressing the matter of disease activity in BD. Additionally, peripheral BDNF levels increased after a successful treatment of an index manic episode but not of a depressive episode – although it should be noted that, in general, treatments for mania are more robustly efficacious than for depression. Insufficient data were available to clarify whether it is a biomarker of treatment response or prognosis.

Lower levels of peripheral BDNF levels in manic and depressive episodes of BD were found in previous meta-analysis on the topic [14–16, 25, 26]. The first three published found BDNF levels decreased in both mania and depression, with normal levels in euthymia [14–16]. More recently, two published meta-analysis suggested decreased levels of peripheral BDNF in bipolar depression but not in mania [25, 26]; however, one [26] had very strict inclusion criteria, and did find BDNF levels decreased in mania after the exclusion of one study which was an outlier [44]. The second one, the most recently published, also did not pinpoint decreased levels of peripheral BDNF in mania; however, it did so in serum when a sub-group analysis was executed. They also found strong evidence of publication bias, with the trim-and-fill estimation suggesting the presence of several missing reports, and the authors proposed that the studies and the literature that suggest that the finding that BDNF levels are decreased in acute episodes of BD might possibly be unreliable due to the presence of publication bias and bias in the individual included studies. However, this later study had an incomplete systematic search and the possible introduction of metabias (bias inserted in a meta-analysis due to issues in the systematic review) – confounding interpretation of this meta-analysis. Although the previous study discussed herein [25] and the present one had very similar inclusion and exclusion criteria, and considered studies published in a similar period of time, we were able to include almost 50 % more studies and almost twice the number of participants (35 vs. 52 studies, and 3,798 vs. 6,481 individual participants, in the study of Munkholm et al*.* [25] and in the present study, respectively). Contrary to the results of Munkholm et al*.* [25], the trim-and-fill method did not suggest any missing studies in the between-group meta-analyses. This conceivably suggests that the discordance between our findings and those of Munkholm et al. [25] may rest on differences in systematic search strategy. Therefore, our results regarding decreased peripheral BDNF levels in mania are arguably a more accurate portrait of the reality than the null results depicted by Munkholm et al*.* [25].

### Peripheral BDNF as a biomarker in BD

The role of BDNF in serum and plasma as a biomarker in psychiatric conditions, including BD, has been recently debated. We found a negative association between serum and plasma BDNF levels and severity of manic and depressive symptoms in mania and depression, meaning that the higher the severity of manic or depressive symptoms, the lower the BDNF levels. This is in line with a recent meta-analysis conducted by Molendijk et al*.* [91], which also found a negative association with serum BDNF levels and severity of depressive symptoms in drug-free subjects with major depressive disorder. In 2010, we first proposed the measurement of proteins in peripheral blood as a laboratory tool to acquire insight into illness activity, and put forward the idea of peripheral BDNF levels as a biomarker of disease activity in BD [17]. In that preliminary report we found that serum BDNF levels in BD were capable of discriminating subjects in mania from subjects in euthymia and healthy controls with a moderate accuracy of 0.72, and also subjects in depression from subjects in euthymia and healthy controls, again with a moderate accuracy of 0.76 [17]. Subsequently, we investigated if peripheral BDNF levels were decreased to the same extent during acute mood episodes across the schizoaffective spectrum [16], and uncovered that serum and plasma BDNF levels are equally decreased during acute mood episodes of BD and major depressive disorder, and in schizophrenia, and that its levels were normal in BD during euthymia and in major depressive disorder during remission. We confirmed our previous findings in the present study, showing that peripheral BDNF levels diminished similarly in both manic and depressive episodes of BD and were normal in euthymia, and extended our findings by also showing that BDNF levels are decreased in tandem with severity of symptoms. However, the variability and consequent overlap among the results found in acute episodes with those in euthymia were large, thereby preventing its use in isolation in clinical practice as a useful laboratory blood biomarker. This is somehow reflected by the only moderate discriminatory accuracy properties that we described in our earlier findings [17]. In keeping with this view, peripheral BDNF levels hold promise as a biomarker of disease activity in BD, possibly as a component of a panel of several proteins [17]. This approach has also been proposed by others [92, 93], although it has been applied to genomics and not to blood protein content.

Recently, using meta-analytic techniques, we established that peripheral BDNF levels are decreased in schizophrenia [94] and in major depressive disorder [91], and that the extent of the decrease is indistinguishable among acute mood states and schizophrenia along the schizoaffective continuum [16]. The ability of a biomarker to usefully support clinical diagnosis in psychiatry would be of great significance [9, 14, 16, 95]. Considering the schizoaffective spectrum, the most valuable diagnostic biomarkers would be those capable of truly differentiating bipolar from unipolar depression and mania from acute schizophrenia [9] – seemingly different pathologies with overlapping symptoms. In 2009, based on preliminary results, we proposed serum BDNF levels as a possible adjunctive tool to discriminate between bipolar and unipolar depression with a high accuracy of 0.95 [9]. However, based on the present data, and on our subsequent meta-analyses on peripheral BDNF in schizophrenia and in major depressive disorder [16, 91, 94], it seems clear that peripheral BDNF levels are not useful as a diagnostic biomarker in psychiatric disorders because of lack of specificity.

In addition to the above, peripheral BDNF has also been suggested as a stage biomarker in psychiatry, capable of capturing the neuroprogressive nature of BD [11]. The neuroprogression hypothesis postulates that the central nervous system pathologically reorganizes during the course of severe mental illness, resulting in alterations that persist even during euthymia [96], and this notion is the core idea supporting the conceptualization of a staging model in BD [97, 98]. Decreased serum BDNF levels were found in the late stages of BD when compared to earlier stages (i.e. more than 10 years and less than 3 years after disease onset, respectively) [62], and a reanalysis of this data proposed that serum BDNF levels are capable of differentiating late stages from early stages with a sensitivity of 100 %, a specificity of 89 %, and an overall accuracy of 0.95 [11]. However, in this study, the subjects in the later stage presented with HDRS scores of 9.2 against 3.8 in the early stages, and provided no data regarding for how long the subjects were in euthymia. This raises the possibility that the decreased serum BDNF levels found in late-stage BD may be an artefact of more severe depressive symptoms, as well as the possibility of being a ‘scar’ of a depressive episode [99]. In our present study, we found absolutely no association between length of illness or age and peripheral BDNF levels in euthymia, suggesting that BDNF does not have value as a biomarker of stage. These findings are in contrast to what is seen in schizophrenia, where there is evidence of a decrease in BDNF levels with age and length of illness [94]. This line of reasoning is also in accordance with a recent study that found normal BDNF levels in the late stage of BD [74]. In addition, there is no correlation between cognition – a domain considered with neuroimaging changes to be a nucleus of allostatic load and the staging model [100] – and peripheral BDNF levels in euthymia [53]. Also, there were no differences in BDNF levels between BD types I and II in euthymia. It is also conceivable that peripheral BDNF levels might only be altered in a subgroup with a more detrimental long BD course, but either way, our current data do not support the measurement of peripheral BDNF levels as a biomarker of staging in BD.

### The neurotrophin hypothesis of BD

Scientists have debated the temporal relationship and the consequent matter of causality concerning alterations in BDNF levels and changes in mood states in BD. It remains unclear whether a decrease in peripheral BDNF levels or the initiation of a mood episode materializes first, and whether an increase in peripheral BDNF levels or the recovery from a mood episode occurs first, or even if changes in BDNF levels and symptoms happen concomitantly. While the temporal behaviour of peripheral BDNF levels preceding a mood episode remains largely unknown, there are far more data regarding its changes after the treatment of an index mood episode. In our longitudinal meta-analyses, we were able to show that peripheral BDNF levels increase following the successful treatment of an index manic episode. Moreover, we suggested that BDNF levels increase in plasma but not in serum, although not proportionally to the severity of manic symptoms as assessed by the YMRS scores, meaning that, if remission is achieved, BDNF levels increase, no matter how severe the manic episode previously was. The fact that plasma BDNF levels increased with an ES of 0.23 with achieving euthymia, while the ES of plasma BDNF levels was −0.72, might suggest that BDNF levels keep increasing after improvement of manic symptoms. Still, we cannot draw any definitive causal association in the matter. In sharp contrast to the increased plasma BDNF levels found with treatment of a manic episode, no increase in peripheral BDNF levels were found after treatment of a depressive episode, regardless of whether it was plasma or serum, and regardless of the presence, or not, of response to treatment. However, it is important to point here that response to treatment in depression was mostly incomplete, with subjects in most studies still presenting depressive symptoms at follow-up.

If one considers this line of reasoning correct, then it is tempting to speculate that decreased levels of BDNF in BD may very well represent an epiphenomenon, without implying causality; in support of this is the fact that the polymorphisms of the *BDNF* gene are not associated with BD [101] or with hippocampal volumes in neuropsychiatric disorders [102]. Most importantly, BDNF levels are ubiquitously decreased across diverse psychiatric pathologies [16, 91, 94], and decreased in both poles of BD – mania and depression – when one would possibly expect opposite behaviours if its levels were causally related to the development of a mood episode. Either way, our results suggest that the neurotrophin pathway is altered in BD, and provide further evidence supporting the neurotrophic hypothesis in mood disorders. In addition, notwithstanding evidence that indicates that peripheral levels of BDNF may reflect BDNF activity in the brain [103, 104], it remains unproved whether peripheral BDNF levels would proxy brain levels of this protein.

### Strengths and limitations

Our study relied on a large sample size (52 studies with 6,481 subjects), which permitted us to draw conclusions through meta-analyses and meta-regression techniques. Our positive results are unlikely to be substantially influenced by publication bias, since the funnel plots in all cases were symmetrical, the trim-and-fill procedure did not point to any missing study necessary to impute in order to ‘correct’ the ES, and in general there were no associations between the magnitude of the pooled ESs and year of publication; however, this cannot be completely excluded in mania, since the presence of an excess of positive (i.e. statistically significant) published studies was suggested by the Ioannidis test. However, the Ioannidis test of excess of significance relies on the assumption that the ‘true’ ES is that of the largest study, and this premise may be threatened when the largest study included is not particularly large or well-conducted, and when there is significant heterogeneity. In both scenarios, the test of excess of significance can falsely signal bias, when in truth what is being pointed out is genuine heterogeneity due to real differences among the different studies among dissimilar populations. Since the largest report included in our meta-analyses included 196 subjects and, in general, there were ample differences in the demographic characteristics of the studies included, this possibility cannot be discarded. In addition, through a series of sensitivity and sub-group analyses, we were able to rule out the possibility that the results were biased due to a unique outlier. The abovementioned approach also allowed us to investigate and rule out any single study as the sole source of the high heterogeneity found in virtually all analyses.

Notwithstanding its strengths, our paper has some inherent limitations due to its design and statistical methods employed. First, meta-analyses are retrospective in nature, affected by the methodological rigour of the studies included, comprehensiveness of search strategies, and possible publication bias. We tried to keep the probability of bias to a minimum by doing a thorough search for data and by using explicit criteria for study selection, data collection, and data analysis. We did not restrict the studies included to those in English, and included not only published articles, but conference proceedings as well, therefore, avoiding missing negative results. This allowed us to conduct the most comprehensive meta-analysis on the topic so far. We believe that using this approach, the results and conclusions can provide reliable information. Second, some of the sub-groups and meta-regression analyses may have failed to achieve statistical significance due to a lack of power in these specific analyses, giving a potentially false negative result. This may have been the reason for the lack of significance of decreased BDNF levels in medicated persons in a manic episode when compared to controls, in mania compared to matched controls by age and sex, and of the association between YMRS and serum BDNF levels. Third, the meta-analysis of BDNF levels in persons with BD compared to controls provides us with a pooled result originating from cross-sectional studies. Therefore, we cannot draw any causal associations. Thus, we do not know if a decrease in peripheral BDNF levels is a cause and pre-requisite for the occurrence of an index mood episode or an allostatic counterbalancing mechanism as a consequence of the occurrence of the mood episode. It is also not possible to infer if peripheral BDNF levels decrease before, concomitantly, or after euthymia is successfully achieved. Fourth, virtually no study included data on length of the acute mood episode, which would be a potentially crucial moderator, if one considers the prospect of peripheral BDNF levels further decreasing during the course of an unmedicated acute mood episode. This appears to be the case in schizophrenia, where a more accentuated decrease in BDNF is present with longer untreated psychosis [105]. Fifth, in our meta-analyses of changes in BDNF levels with treatment for acute mania or depression, we considered the studies as responders or non-responders using the mean values of the YMRS or HDRS scores of each study, and not of individual subjects, with the exception of the study of Rybakowski et al. [78], which provided data for responders and non-responders separately. This may specifically have been an issue in the meta-analysis regarding treatment of depression, where probably the sample of each study has more variability in response than in the mania studies. It should also be noted that, in depression, the greatest mean response rate was 65 %, that none achieved remission, and that in all studies, subjects had at least residual manifestations of depression. Consequently, the absence of an increase in BDNF levels after treatment of a depressive episode may indicate an absence of substantial improvement. In addition, there was a manifold of distinct medications employed in the therapeutics of the depressive episodes, ranging from quetiapine, an approved medication for this condition, to valproate, ketamine and mifepristone, which can be considered experimental in this situation. Sixth, we used length of illness as a proxy for stage. Finally, as in other meta-analyses, our results should be interpreted with caution because individual studies varied greatly with respect to the demographic characteristics and ethnicity of participants, type of psychiatric medication on use, and duration of follow-up in the within-group meta-analyses.

### Future research

The temporal changes in BDNF levels in relation to a mood episode remains one of the known unknowns of science. Peripheral BDNF level changes in relation to the occurrence of a mood episode could theoretically follow three different patterns. First, peripheral BDNF could decrease before the beginning of a mood episode, which would make BDNF a possible predictor of a future mood episode; second, BDNF levels could decrease concomitantly with a mood episode, turning BDNF into a biomarker of disease activity; and finally, peripheral BDNF levels could decrease after the beginning of a mood episode. In this case, BDNF changes would be a consequence of a mood episode, and its assessment could be useful perhaps as a surrogate biomarker. To provide a definitive answer to these questions a longitudinal study with frequent blood draws would be necessary, with a within-subject design that also reduces noise and consequently is less prone to bias. It is also true that a biomarker may be capable of measuring a variety of clinical endpoints simultaneously.

One potentially interesting application of peripheral BDNF levels is as a biomarker for predicting response to treatment in an index mood episode. It would be of great value to determine if baseline levels of peripheral BDNF can predict response to treatment, differing between those who achieve remission and identifying those who will be refractory. This is a simple analysis that can be easily performed; however, it has been overlooked in almost all studies. Data on this regard is scant and the field would benefit from such investigation.

There is evidence from our present study that BDNF levels can vary according to its source, meaning plasma or serum. For instance, we found an association in the meta-regressions between severity of manic and depressive episodes, mostly in plasma, and in the within-group meta-analysis BDNF increased after treatment of a manic episode in plasma, but not in serum. We previously described a similar pattern in schizophrenia, with antipsychotics increasing BDNF levels only in plasma [94]. At the moment, most of the BDNF enquiries in BD have used serum, with far less studies conducted in plasma. However, serum and plasma represent, in fact, two different compartments. This is highlighted by the fact that BDNF concentrations in serum are 20- to 50-times higher than in plasma [106]. Peripheral BDNF is largely stored in platelets, which actively absorb it from the circulation, and is released from activated platelets in serum during the clotting process, and consequently serum BDNF levels largely reflect the pool of BDNF stored and released from platelets during coagulation [107]. However, after being produced by the brain and released into the circulation, BDNF has a half-life of less than 10 minutes in the blood and is rapidly cleared from the circulation mostly by the liver, in contrast to a platelet’s life-span of 9–11 days [108]. This logically implies that plasma BDNF levels may be a more accurate marker of acute changes in the central nervous system than serum BDNF. This may be because of the fact that the still ongoing release of BDNF from platelets obscures serum BDNF levels, with ‘old’ BDNF actually being measured. We therefore suggest that future research should preferentially consider plasma when assessing the BDNF as a potential clinical biomarker. We could trace a parallel with diabetes, where plasma would behave as fasting glycaemia, representing acute changes in glycaemia, and serum as glycated haemoglobin, representing a general picture of glycaemia in the last 90 days. Since the life-span of platelets is 10 days, serum BDNF levels would better represent the general behaviour of BDNF in last 10 days, and plasma would represent acute changes in BDNF. Finally, in our analyses, we uncovered that studies with an ‘imperfect’ pairing of the control group regarding age and sex may be more susceptible to bias, particularly with differences in age. Lack of matching increases noise and unduly distorts data. This is a concern not only for studies on BDNF levels, but for all studies investigating biomarker properties. We suggest that future study design should take this into consideration.

## Conclusions

Our meta-analyses of 52 cross-sectional or longitudinal studies comprising 6,481 persons with BD and healthy controls provide evidence that peripheral BDNF levels are equally decreased in BD during the occurrence of manic and depressive episodes, and that its levels appear normal in euthymia. The extent of the decrease in peripheral BDNF levels paralleled the severity of manic and depressive symptoms, providing further evidence of peripheral BDNF as a state-marker and consequentially as a biomarker of disease activity for acute mood episodes of BD. Our study also provides further evidence of the neurotrophic hypothesis of BD, and solidifies the notion of BD as a systemic disorder with peripheral manifestations. In summary, peripheral BDNF level, better documented in plasma than in serum, is a potential biomarker of disease activity in BD. We propose that peripheral BDNF may have a clinical application as a part of a laboratory blood protein composite measure to assess disease activity in BD.

## Additional files

Additional file 1:
**Figure S1.** PRISMA flowchart of the meta-analytic review. **Figure S2.** Forest plot for random effects between-group meta-analysis of peripheral BDNF levels in participants with bipolar disorder on a mixed episode. **Figure S3.** Sensitivity analysis of included studies in between-group meta-analyses of peripheral BDNF levels in participants with bipolar disorder in mania. **Figure S4.** Sensitivity analysis of included studies in between-group meta-analyses of peripheral BDNF levels in participants with bipolar disorder in depression. **Figure S5.** Sensitivity analysis of included studies in between-group meta-analyses of peripheral BDNF levels in participants with bipolar disorder in euthymia. **Figure S6.** Sensitivity analysis of included studies in within-group meta-analyses of peripheral BDNF levels in participants with bipolar disorder in mania. **Figure S7.** Sensitivity analysis of included studies in within-group meta-analyses of peripheral BDNF levels in participants with bipolar disorder in depression. **Figure S8.** Cumulative Meta-Analysis of included studies in between-group meta-analyses of peripheral BDNF levels in participants with mania. **Figure S9.** Cumulative Meta-Analysis of included studies in between-group meta-analyses of peripheral BDNF levels in participants with depression. **Figure S10.** Cumulative Meta-Analysis of included studies in between-group meta-analyses of peripheral BDNF levels in participants with euthymia. **Figure S11.** Cumulative Meta-Analysis of included studies in within-group meta-analyses of peripheral BDNF levels in participants with bipolar disorder in mania. **Figure S12.** Cumulative Meta-Analysis of included studies in within-group meta-analyses of peripheral BDNF levels in participants with bipolar disorder in depression. **Figure S13.** Funnel plot of included studies in between-group meta-analyses of peripheral BDNF levels in participants with bipolar disorder in mania compared to healthy controls. The white diamond shows the observed summary effect size. The black diamond shows the adjusted effect size after the trim and fill procedure. **Figure S14.** Funnel plot of included studies in between-group meta-analyses of peripheral BDNF levels in participants with bipolar disorder in depression compared to healthy controls. **Figure S15.** Funnel plot of included studies in between-group meta-analyses of peripheral BDNF levels in participants with bipolar disorder in euthymia compared to healthy controls. **Figure S16.** Funnel plot of included studies in within-group meta-analyses of peripheral BDNF level in participants with bipolar disorder in mania before and after treatment. **Figure S17.** Funnel plot of included studies in within-group meta-analyses of peripheral BDNF levels in participants with bipolar disorder in depression before and after treatment. (PDF 567 kb) Additional file 2:
**Table S1.** Studies excluded in secondary screening (with reasons). **Table S2.** Characteristics of studies included in between-group meta-analyses of peripheral BDNF levels in bipolar disorder, across different mood states. **Table S3.** Characteristics of studies included in within-group meta-analyses of peripheral BDNF levels in bipolar disorder across different mood states. **Table S4.** Characteristics of meta-analyses of peripheral BDNF in bipolar disorder. **Table S5.** Assessment of bias across meta-analyses of peripheral BDNF in bipolar disorder. (PDF 690 kb)

## Abbreviations

BD: Bipolar disorder
BDNF: Brain-derived neurotrophic factor
CI: Confidence interval
ES: Effect size
HDRS: Hamilton Depression Rating Scale
PRISMA: Preferred Reporting Items for Systematic Reviews and Meta-Analyses
YMRS: Young Mania Rating Scale

## References

[1] Barde YA, Edgar D, Thoenen H (1982). Purification of a new neurotrophic factor from mammalian brain. EMBO J.

[2] Levi-Montalcini R, Hamburger V (1951). Selective growth stimulating effects of mouse sarcoma on the sensory and sympathetic nervous system of the chick embryo. J Exp Zool.

[3] Duman RS, Heninger GR, Nestler EJ (1997). A molecular and cellular theory of depression. Arch Gen Psychiatry.

[4] Karege F, Perret G, Bondolfi G, Schwald M, Bertschy G, Aubry JM (2002). Decreased serum brain-derived neurotrophic factor levels in major depressed patients. Psychiatry Res.

[5] Kuhn T (1962). The structure of scientific revolutions.

[6] Toyooka K, Asama K, Watanabe Y, Muratake T, Takahashi M, Someya T (2002). Decreased levels of brain-derived neurotrophic factor in serum of chronic schizophrenic patients. Psychiatry Res.

[7] Laske C, Stransky E, Eschweiler GW, Wittorf A, Richartz-Salzburger E, Bartels M (2005). Brain-derived neurotrophic factor (BDNF) in serum of patients with mania, major depression and healthy controls. Neurol Psychiatry Brain Res.

[8] Palomino A, Vallejo-Illarramendi A, Gonzalez-Pinto A, Aldama A, Gonzalez-Gomez C, Mosquera F (2006). Decreased levels of plasma BDNF in first-episode schizophrenia and bipolar disorder patients. Schizophr Res.

[9] Fernandes BS, Gama CS, Kauer-Sant'Anna M, Lobato MI, Belmonte-de-Abreu P, Kapczinski F (2009). Serum brain-derived neurotrophic factor in bipolar and unipolar depression: a potential adjunctive tool for differential diagnosis. J Psychiatr Res.

[10] Frey BN, Andreazza AC, Houenou J, Jamain S, Goldstein BI, Frye MA (2013). Biomarkers in bipolar disorder: a positional paper from the International Society for Bipolar Disorders Biomarkers Task Force. Aust N Z J Psychiatry.

[11] Kapczinski F, Fernandes BS, Kauer-Sant'Anna M, Gama CS, Yatham LN, Berk M (2009). The concept of staging in bipolar disorder: the role of BDNF and TNF-alpha as biomarkers. Acta Neuropsychiatrica.

[12] Pan W, Banks WA, Fasold MB, Bluth J, Kastin AJ (1998). Transport of brain-derived neurotrophic factor across the blood–brain barrier. Neuropharmacology.

[13] Karege F, Schwald M, Cisse M (2002). Postnatal developmental profile of brain-derived neurotrophic factor in rat brain and platelets. Neurosci Lett.

[14] Fernandes BS, Gama CS, Maria Cereser K, Yatham LN, Fries GR, Colpo G (2011). Brain-derived neurotrophic factor as a state-marker of mood episodes in bipolar disorders: a systematic review and meta-regression analysis. J Psychiatr Res.

[15] Lin PY (2009). State-dependent decrease in levels of brain-derived neurotrophic factor in bipolar disorder: a meta-analytic study. Neurosci Lett.

[16] Fernandes BS, Berk M, Turck CW, Steiner J, Goncalves CA (2014). Decreased peripheral brain-derived neurotrophic factor levels are a biomarker of disease activity in major psychiatric disorders: a comparative meta-analysis. Mol Psychiatry.

[17] Fernandes BS, Gomes FA, Fries G, Stertz L, Cereser KM, Pessoa C (2010). Brain-derived neurotrophic factor as a possible biomarker of bipolar disorder activity. Bipolar Disord..

[18] Fernandes BS, Steiner J, Bernstein HG, Dodd S, Pasco JA, Dean OM, et al. C-reactive protein is increased in schizophrenia but is not altered by antipsychotics: meta-analysis and implications. Mol Psychiatry. 2015 Jul 14. doi:10.1038/mp.2015.87. [Epub ahead of print]. 10.1038/mp.2015.87 26169974

[19] Rosa AR, Gonzalez-Ortega I, Gonzalez-Pinto A, Echeburua E, Comes M, Martinez-Aran A (2012). One-year psychosocial functioning in patients in the early vs. late stage of bipolar disorder. Acta Psychiatr Scand.

[20] Davis J, Maes M, Andreazza A, McGrath JJ, Tye SJ, Berk M (2015). Towards a classification of biomarkers of neuropsychiatric disease: from encompass to compass. Mol Psychiatry.

[21] Higgins JPT, Green S. Cochrane Handbook for Systematic Reviews of Interventions Version 5.1.0. The Cochrane Collaboration, 2011*.* The Cochrane Collaboration. http://www.cochrane-handbook.org.

[22] Stroup DF, Berlin JA, Morton SC, Olkin I, Williamson GD, Rennie D (2000). Meta-analysis of observational studies in epidemiology: a proposal for reporting. Meta-analysis Of Observational Studies in Epidemiology (MOOSE) group. JAMA.

[23] Lau J, Ioannidis JP, Schmid CH (1997). Quantitative synthesis in systematic reviews. Ann Intern Med.

[24] Higgins JP, Thompson SG (2002). Quantifying heterogeneity in a meta-analysis. Stat Med.

[25] Munkholm K, Vinberg M, Kessing LV. Peripheral blood brain-derived neurotrophic factor in bipolar disorder: a comprehensive systematic review and meta-analysis. Mol Psychiatry. 2015. Ahead of print. doi: 10.1038/mp.2015.54. 10.1038/mp.2015.54 26194180

[26] Polyakova M, Stuke K, Schuemberg K, Mueller K, Schoenknecht P, Schroeter ML (2015). BDNF as a biomarker for successful treatment of mood disorders: a systematic & quantitative meta-analysis. J Affect Disord..

[27] Moher D, Liberati A, Tetzlaff J, Altman DG, PRISMA Group (2009). Preferred reporting items for systematic reviews and meta-analyses: the PRISMA statement. J Clin Epidemiol.

[28] Hamilton M (1960). A rating scale for depression. J Neurol Neurosurg Psychiatry.

[29] Sistrom CL, Mergo PJ (2000). A simple method for obtaining original data from published graphs and plots. AJR Am J Roentgenol.

[30] Dwan K, Gamble C, Williamson PR, Kirkham JJ (2013). Systematic review of the empirical evidence of study publication bias and outcome reporting bias - an updated review. PLoS One.

[31] Young SS, Bang H (2004). The file-drawer problem, revisited. Sci.

[32] Egger M, Davey Smith G, Schneider M, Minder C (1997). Bias in meta-analysis detected by a simple, graphical test. BMJ.

[33] Belbasis L, Bellou V, Evangelou E, Ioannidis JP, Tzoulaki I (2015). Environmental risk factors and multiple sclerosis: an umbrella review of systematic reviews and meta-analyses. Lancet Neurol.

[34] Huedo-Medina TB, Sánchez-Meca J, Marin-Martinez F, Botella J (2006). Assessing heterogeneity in meta-analysis: Q statistic or I^2^ index?. Psychol Methods.

[35] Higgins JP, Thompson SG, Deeks JJ, Altman DG (2003). Measuring inconsistency in meta-analyses. BMJ.

[36] Higgins JP, Thompson SG, Spiegelhalter DJ (2009). A re-evaluation of random-effects meta-analysis. J R Stat Society Ser A.

[37] Higgins JP (2008). Commentary: Heterogeneity in meta-analysis should be expected and appropriately quantified. Int J Epidemiol.

[38] Higgins J, Green, S. Cochrane Handbook for systematic reviews of interventions version 5.0. 2 [updated September 2009]. The Cochrane Collaboration, 2009. www.cochrane-handbook.org. Accessed 18 August 2014.

[39] Faul F, Erdfelder E, Buchner A, Lang AG (2009). Statistical power analyses using G*Power 3.1: tests for correlation and regression analyses. Behav Res Methods.

[40] Ioannidis JP, Trikalinos TA (2007). An exploratory test for an excess of significant findings. Clin Trials.

[41] Munkholm K, Pedersen BK, Kessing LV, Vinberg M (2014). Elevated levels of plasma brain derived neurotrophic factor in rapid cycling bipolar disorder patients. Psychoneuroendocrinology..

[42] Andreazza AC, Rajji TK, Gildengers A, Soares AT, Lafer B, Young LT (2014). Decrease brain-derived neurotrophic factor (BDNF) in older patients with bipolar disorder. Biol Psychiatry..

[43] Barbosa IG, Huguet RB, Mendonca VA, Neves FS, Reis HJ, Bauer ME (2010). Increased plasma levels of brain-derived neurotrophic factor in patients with long-term bipolar disorder. Neurosci Lett.

[44] Barbosa IG, Rocha NP, de Miranda AS, Huguet RB, Bauer ME, Reis HJ (2013). Increased BDNF levels in long-term bipolar disorder patients. Rev Bras Psiquiatr.

[45] Barbosa IG, Rocha NP, Huguet RB, Ferreira RA, Salgado JV, Carvalho LA (2012). Executive dysfunction in euthymic bipolar disorder patients and its association with plasma biomarkers. J Affect Disord.

[46] Ceylan D, Ozerdem A, Gurz Yalcin SN, Hidirotlu C, Aslan YC, Batci B (2012). Can serum BDNF levels be identified as a candidate endophenotype in bipolar disorder?. Bipolar Disord..

[47] Chen SL, Lee SY, Chang YH, Chen SH, Chu CH, Wang TY (2014). The BDNF Val66Met polymorphism and plasma brain-derived neurotrophic factor levels in Han Chinese patients with bipolar disorder and schizophrenia. Prog Neuro-Psychopharmacol Biol Psychiatry..

[48] Chou YH, Wang SJ, Lirng JF, Lin CL, Yang KC, Chen CK (2012). Impaired cognition in bipolar I disorder: the roles of the serotonin transporter and brain-derived neurotrophic factor. J Affect Disord.

[49] Cunha AB, Frey BN, Andreazza AC, Goi JD, Rosa AR, Goncalves CA (2006). Serum brain-derived neurotrophic factor is decreased in bipolar disorder during depressive and manic episodes. Neurosci Lett.

[50] de Oliveira GS, Cereser KM, Fernandes BS, Kauer-Sant'Anna M, Fries GR, Stertz L (2009). Decreased brain-derived neurotrophic factor in medicated and drug-free bipolar patients. J Psychiatr Res.

[51] de Sousa RT, van de Bilt MT, Diniz BS, Ladeira RB, Portela LV, Souza DO (2011). Lithium increases plasma brain-derived neurotrophic factor in acute bipolar mania: a preliminary 4-week study. Neurosci Lett.

[52] Dell'Osso L, Bianchi C, Del Debbio A, Roncaglia I, Veltri A, Carlini M (2010). Plasma brain-derived neurotrophic factor in bipolar and unipolar depression. Ital J Psychopathol.

[53] Dias VV, Brissos S, Frey BN, Andreazza AC, Cardoso C, Kapczinski F (2009). Cognitive function and serum levels of brain-derived neurotrophic factor in patients with bipolar disorder. Bipolar Disord.

[54] F PBW-ABPSLCMVEFMKMK (2013). Evaluation of peripheral biomarkers in bipolar and unipolar depression. Eur Neuropsychopharmacol.

[55] Goka E, Goka S, Aydemir C, Aksaray S, Yalcin ES, Kisa C. [BDNF levels and change with treatment in patients with bipolar disorder manik episode.] Klin Psikofarmakoloji Bulteni. 2009;19 Suppl 1:S8–S13. In Turkish.

[56] Grande I, Kapczinski F, Stertz L, Colpo GD, Kunz M, Cereser KM (2012). Peripheral brain-derived neurotrophic factor changes along treatment with extended release quetiapine during acute mood episodes: An open-label trial in drug-free patients with bipolar disorder. J Psychiatr Res.

[57] Grande I, Magalhaes PV, Chendo I, Stertz L, Fries GR, Cereser KM (2014). Val66Met polymorphism and serum brain-derived neurotrophic factor in bipolar disorder: an open-label trial. Acta Psychiatr Scand.

[58] Hsieh WC, Jou YT, Lin JL, Wang SJ, Chou YH (2014). The effect of cortisol and BDNF on serotonin transporter in bipolar I disorder. Bipolar Disord..

[59] Huang TL, Hung YY, Lee CT, Chen RF (2012). Serum protein levels of brain-derived neurotrophic factor and tropomyosin-related kinase B in bipolar disorder: effects of mood stabilizers. Neuropsychobiology.

[60] Kapczinski F, Dal-Pizzol F, Teixeira AL, Magalhaes PV, Kauer-Sant'Anna M, Klamt F (2011). Peripheral biomarkers and illness activity in bipolar disorder. J Psychiatr Res.

[61] Karamustafalioglu N, Genc A, Kalelioglu T, Tasdemir A, Umut G, Incir S (2015). Plasma BDNFs level initially and post treatment in acute mania: comparison between ECT and atypical antipsychotic treatment and healthy controls. J Psychopharmacol.

[62] Kauer-Sant'Anna M, Kapczinski F, Andreazza AC, Bond DJ, Lam RW, Young L (2009). Brain-derived neurotrophic factor and inflammatory markers in patients with early- vs. late-stage bipolar disorder. Int J Neuropsychopharmacol.

[63] Kenna HA, Reynolds-May M, Stepanenko A, Ketter TA, Hallmayer J, Rasgon NL (2014). Blood levels of brain derived neurotrophic factor in women with bipolar disorder and healthy control women. J Affect Disord..

[64] Kim Y-K, Na K-S, Hwang J-A, Yoon H-K, Lee H-J, Hahn S-W (2013). High insulin-like growth factor-1 in patients with bipolar I disorder: a trait marker?. J Affect Disord.

[65] Kurita M, Nishino S, Numata Y, Okubo Y, Sato T (2014). The noradrenaline metabolite MHPG is a candidate biomarker from the manic to the remission state in bipolar disorder I: a clinical naturalistic study. PLoS One.

[66] Langan C, Doyle K, Kelly J, Emsell L, Skinner R, McDonald C (2009). Serum BDNF levels in euthymic bipolar disorder: Preliminary results from the Galway bipolar study. Bipolar Disord.

[67] Lee SY, Chen SL, Chang YH, Chen PS, Huang SY, Tzeng NS (2015). Correlation of plasma brain-derived neurotrophic factor and metabolic profiles in drug-naive patients with bipolar II disorder after a twelve-week pharmacological intervention. Acta Psychiatrica Scandinavica.

[68] Li Z, Zhang C, Fan J, Yuan C, Huang J, Chen J (2014). Brain-derived neurotrophic factor levels and bipolar disorder in patients in their first depressive episode: 3-year prospective longitudinal study. Br J Psychiatry.

[69] Lotrich FE, Butters MA, Aizenstein H, Marron MM (2014). Reynolds 3rd CF, Gildengers AG. The relationship between interleukin-1 receptor antagonist and cognitive function in older adults with bipolar disorder. Int J Geriatr Psychiatry.

[70] Machado-Vieira R, Dietrich MO, Leke R, Cereser VH, Zanatto V, Kapczinski F (2007). Decreased plasma brain derived neurotrophic factor levels in unmedicated bipolar patients during manic episode. Biol Psychiatry.

[71] Mackin P, Gallagher P, Watson S, Young A, Ferrier IN (2007). Changes in brain-derived neurotrophic factor following treatment with mifepristone in bipolar disorder and schizophrenia. Aust N Z J Psychiatry.

[72] Magalhaes PV, Jansen K, Pinheiro RT, Fries GR, Teixeira AL, Da Silva RA (2012). A nested population-based case–control study on peripheral inflammation markers and brain-derived neurotrophic factor in early-stage mood disorders. Bipolar Disord..

[73] Monteleone P, Serritella C, Martiadis V, Maj M (2008). Decreased levels of serum brain-derived neurotrophic factor in both depressed and euthymic patients with unipolar depression and in euthymic patients with bipolar I and II disorders. Bipolar Disord.

[74] Panizzutti BS, Gubert C, Schuh AL, Ferrari P, Bristot G, Fries GR (2014). Increased serum levels of CCL11/eotaxin in late stage bipolar patients. Biol Psychiatry..

[75] Piccinni A, Veltri A, Costanzo D, Vanelli F, Franceschini C, Moroni I (2014). Decreased plasma levels of brain-derived neurotrophic factor (BDNF) during mixed episodes of bipolar disorder. J Affect Disord.

[76] Rabie MA, Mohsen M, Ibrahim M, El-Sawy Mahmoud R (2014). Serum level of brain derived neurotrophic factor (BDNF) among patients with bipolar disorder. J Affect Disord..

[77] Rosa AR, Singh N, Whitaker E, de Brito M, Lewis AM, Vieta E (2014). Altered plasma glutathione levels in bipolar disorder indicates higher oxidative stress; a possible risk factor for illness onset despite normal brain-derived neurotrophic factor (BDNF) levels. Psychol Med..

[78] Rybakowski JK, Permoda-Osip A, Skibinska M, Adamski R, Bartkowska-Sniatkowska A (2013). Single ketamine infusion in bipolar depression resistant to antidepressants: are neurotrophins involved?. Hum Psychopharmacol.

[79] Rybakowski JK, Suwalska A (2010). Excellent lithium responders have normal cognitive functions and plasma BDNF levels. Int J Neuropsychopharmacol.

[80] Sodersten K, Palsson E, Ishima T, Funa K, Landen M, Hashimoto K (2014). Abnormality in serum levels of mature brain-derived neurotrophic factor (BDNF) and its precursor proBDNF in mood-stabilized patients with bipolar disorder: a study of two independent cohorts. J Affect Disord..

[81] Su SC, Sun MT, Wen MJ, Lin CJ, Chen YC, Hung YJ (2011). Brain-derived neurotrophic factor, adiponectin, and proinflammatory markers in various subtypes of depression in young men. Int J Psychiatry Med.

[82] Suwalska A, Sobieska M, Rybakowski J (2010). Serum brain-derived neurotrophic factor in euthymic bipolar patients on prophylactic lithium therapy. Neuropsychobiology.

[83] Tramontina J, Frey B, Andreazza A, Zandona M, Santin A, Kapczinski F (2007). Val66met polymorphism and serum brain-derived neurotrophic factor levels in bipolar disorder. Mol Psychiatry.

[84] Tramontina JF, Andreazza AC, Kauer-Sant'anna M, Stertz L, Goi J, Chiarani F (2009). Brain-derived neurotrophic factor serum levels before and after treatment for acute mania. Neurosci Lett.

[85] Tunca Z, Kivircik Akdede B, Ozerdem A, Alkin T, Polat S, Ceylan D (2015). Diverse glial cell line-derived neurotrophic factor (GDNF) support between mania and schizophrenia: a comparative study in four major psychiatric disorders. Eur Psychiatry.

[86] Tunca Z, Ozerdem A, Ceylan D, Yalcin Y, Can G, Resmi H (2014). Alterations in BDNF (brain derived neurotrophic factor) and GDNF (glial cell line-derived neurotrophic factor) serum levels in bipolar disorder: the role of lithium. J Affect Disord..

[87] Wang ZW, Li ZZ, Lin ZG, Wu ZG, Yuan CM, Hong W (2011). Changes of plasma brain-derived neurotrophic factor in patients with bipolar disorder type I. J Shanghai Jiaotong Univ.

[88] Ye C, Xu Y, Hu H (2010). The serum concentration of brain-derived neurotrophic factor (BDNF) increased after four weeks treatment in acute mania patients. Int J Neuropsychopharmacol..

[89] Yoshimura R, Ikenouchi-Sugita A, Hori H, Umene-Nakano W, Katsuki A, Hayashi K (2010). Adding a low dose atypical antipsychotic drug to an antidepressant induced a rapid increase of plasma brain-derived neurotrophic factor levels in patients with treatment-resistant depression. Prog Neuro-Psychopharmacol Biol Psychiatry.

[90] Yoshimura R, Nakano Y, Hori H, Ikenouchi A, Ueda N, Nakamura J (2006). Effect of risperidone on plasma catecholamine metabolites and brain-derived neurotrophic factor in patients with bipolar disorders. Hum Psychopharmacol.

[91] Molendijk ML, Spinhoven P, Polak M, Bus BA, Penninx BW, Elzinga BM (2014). Serum BDNF concentrations as peripheral manifestations of depression: evidence from a systematic review and meta-analyses on 179 associations (N = 9484). Mol Psychiatry.

[92] Munkholm K, Peijs L, Vinberg M, Kessing LV (2015). A composite peripheral blood gene expression measure as a potential diagnostic biomarker in bipolar disorder. Transl Psychiatry..

[93] Niculescu AB, Le-Niculescu H, Patel S, Bhat M, Kuczenski R, Faraone SV (2009). Convergent functional genomics of genome-wide association data for bipolar disorder: Comprehensive identification of candidate genes, pathways and mechanisms. Biol Psychiatry..

[94] Fernandes BS, Steiner J, Berk M, Molendijk ML, Gonzalez-Pinto A, Turck CW (2015). Peripheral brain-derived neurotrophic factor in schizophrenia and the role of antipsychotics: meta-analysis and implications. Mol Psychiatry.

[95] Carvalho AF, Kohler CA, McIntyre RS, Knochel C, Brunoni AR, Thase ME (2015). Peripheral vascular endothelial growth factor as a novel depression biomarker: a meta-analysis. Psychoneuroendocrinology..

[96] Berk M, Kapczinski F, Andreazza AC, Dean OM, Giorlando F, Maes M (2011). Pathways underlying neuroprogression in bipolar disorder: focus on inflammation, oxidative stress and neurotrophic factors. Neurosci Biobehav Rev.

[97] Kapczinski F, Magalhaes PV, Balanza-Martinez V, Dias VV, Frangou S, Gama CS (2014). Staging systems in bipolar disorder: an International Society for Bipolar Disorders Task Force Report. Acta Psychiatr Scand.

[98] Berk M, Berk L, Dodd S, Cotton S, Macneil C, Daglas R (2014). Stage managing bipolar disorder. Bipolar Disord.

[99] Bus BA, Molendijk ML, Tendolkar I, Penninx BW, Prickaerts J, Elzinga BM (2015). Chronic depression is associated with a pronounced decrease in serum brain-derived neurotrophic factor over time. Mol Psychiatry.

[100] Vieta E (2015). Staging and psychosocial early intervention in bipolar disorder. Lancet Psychiatry.

[101] Gonzalez-Castro TB, Nicolini H, Lanzagorta N, Lopez-Narvaez L, Genis A, Pool Garcia S (2015). The role of brain-derived neurotrophic factor (BDNF) Val66Met genetic polymorphism in bipolar disorder: a case–control study, comorbidities, and meta-analysis of 16,786 subjects. Bipolar Disord.

[102] Harrisberger F, Smieskova R, Schmidt A, Lenz C, Walter A, Wittfeld K (2015). BDNF Val66Met polymorphism and hippocampal volume in neuropsychiatric disorders: a systematic review and meta-analysis. Neurosci Biobehav Rev..

[103] Klein AB, Williamson R, Santini MA, Clemmensen C, Ettrup A, Rios M (2011). Blood BDNF concentrations reflect brain-tissue BDNF levels across species. Int J Neuropsychopharmacol.

[104] Dawood T, Anderson J, Barton D, Lambert E, Esler M, Hotchkin E (2007). Reduced overflow of BDNF from the brain is linked with suicide risk in depressive illness. Mol Psychiatry.

[105] Rizos EN, Michalopoulou PG, Siafakas N, Stefanis N, Douzenis A, Rontos I (2010). Association of serum brain-derived neurotrophic factor and duration of untreated psychosis in first-episode patients with schizophrenia. Neuropsychobiology.

[106] Pillai A, Kale A, Joshi S, Naphade N, Raju MS, Nasrallah H (2010). Decreased BDNF levels in CSF of drug-naive first-episode psychotic subjects: correlation with plasma BDNF and psychopathology. Int J Neuropsychopharmacol.

[107] Fujimura H, Altar CA, Chen R, Nakamura T, Nakahashi T, Kambayashi J (2002). Brain-derived neurotrophic factor is stored in human platelets and released by agonist stimulation. Thromb Haemost.

[108] Baseri B, Choi JJ, Deffieux T, Samiotaki G, Tung YS, Olumolade O (2012). Activation of signaling pathways following localized delivery of systemically administered neurotrophic factors across the blood–brain barrier using focused ultrasound and microbubbles. Phys Med Biol.

