# Supplementary material for: Peripheral brain-derived neurotrophic factor (BDNF) as a biomarker in bipolar disorder: a meta-analysis of 52 studies
Source: BMC Med. 2015 Nov 30;13:289. doi: 10.1186/s12916-015-0529-7 (PMC4666054; doi:10.1186/s12916-015-0529-7)
Supplement: Additional file 1: — Figure S1. PRISMA flowchart of the meta-analytic review. Figure S2. Forest plot for random effects between-group meta-analysis of peripheral BDNF levels in participants with bipolar disorder on a mixed episode. Figure S3. Sensitivity analysis of included studies in between-group meta-analyses of peripheral BDNF levels in participants with bipolar disorder in mania. Figure S4. Sensitivity analysis of included studies in between-group meta-analyses of peripheral BDNF levels in participants with bipolar disorder in depression. Figure S5. Sensitivity analysis of included studies in between-group meta-analyses of peripheral BDNF levels in participants with bipolar disorder in euthymia. Figure S6. Sensitivity analysis of included studies in within-group meta-analyses of peripheral BDNF levels in participants with bipolar disorder in mania. Figure S7. Sensitivity analysis of included studies in within-group meta-analyses of peripheral BDNF levels in participants with bipolar disorder in depression. Figure S8. Cumulative Meta-Analysis of included studies in between-group meta-analyses of peripheral BDNF levels in participants with mania. Figure S9. Cumulative Meta-Analysis of included studies in between-group meta-analyses of peripheral BDNF levels in participants with depression. Figure S10. Cumulative Meta-Analysis of included studies in between-group meta-analyses of peripheral BDNF levels in participants with euthymia. Figure S11. Cumulative Meta-Analysis of included studies in within-group meta-analyses of peripheral BDNF levels in participants with bipolar disorder in mania. Figure S12. Cumulative Meta-Analysis of included studies in within-group meta-analyses of peripheral BDNF levels in participants with bipolar disorder in depression. Figure S13. Funnel plot of included studies in between-group meta-analyses of peripheral BDNF levels in participants with bipolar disorder in mania compared to healthy controls. The white diamond shows the observed summary effect size [file 12916_2015_529_MOESM1_ESM.pdf]

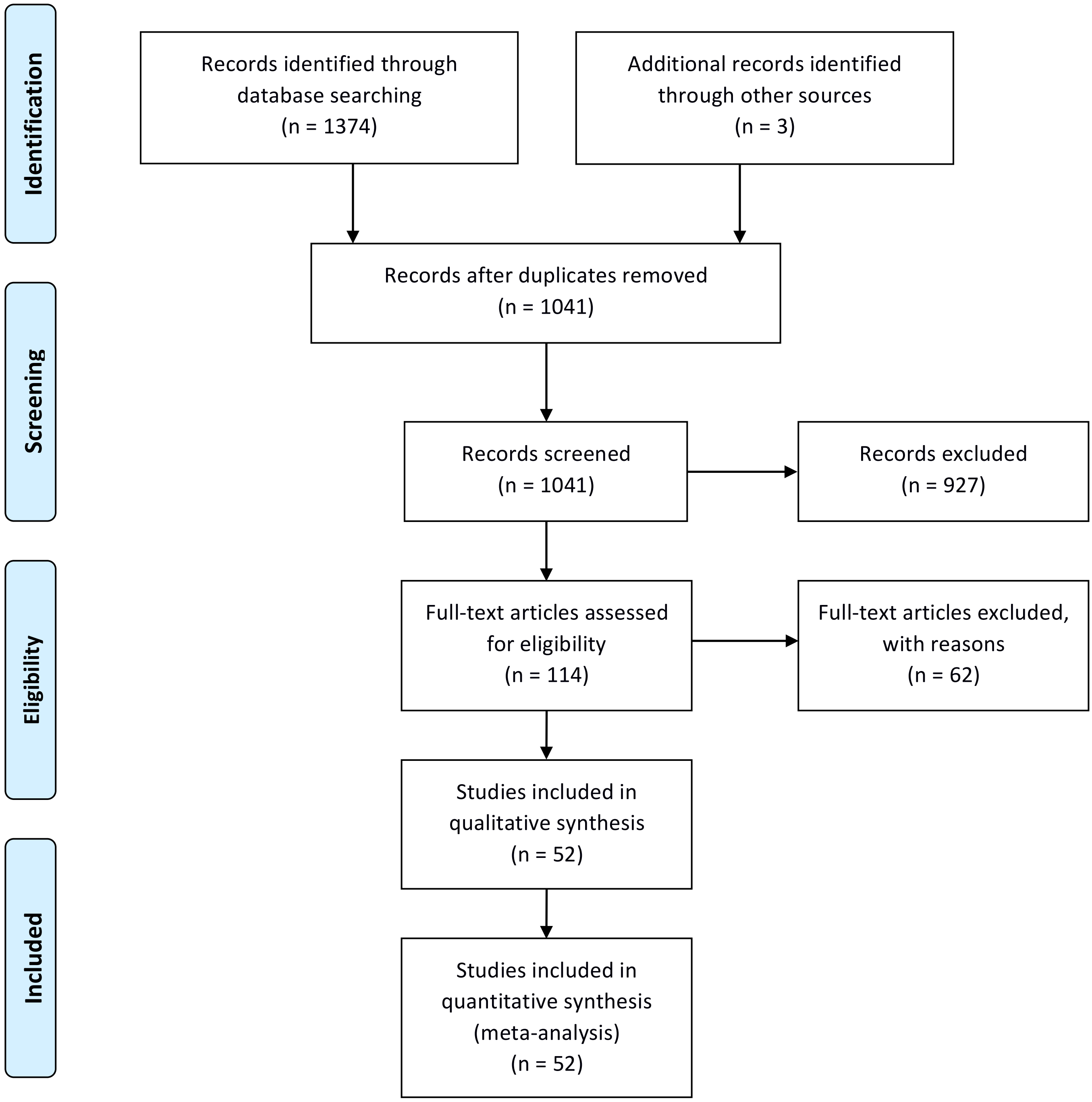

Figure S1. PRISMA flowchart of the meta-analytic review.

Study name	Hedges's g	p-Value
Magalhaes (2012)	-0.14	0.67
Grande (2014)	-0.32	0.27
Piccini (2015)	0.79	0.02
	0.09	0.79

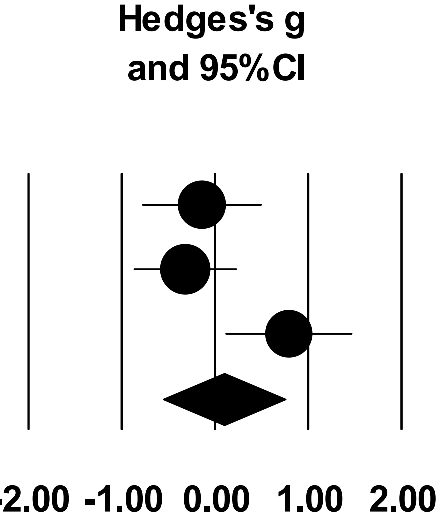

Figure S2. Forest plot for random effects between-group meta-analysis of peripheral BDNF levels in participants with bipolar disorder on a mixed episode.

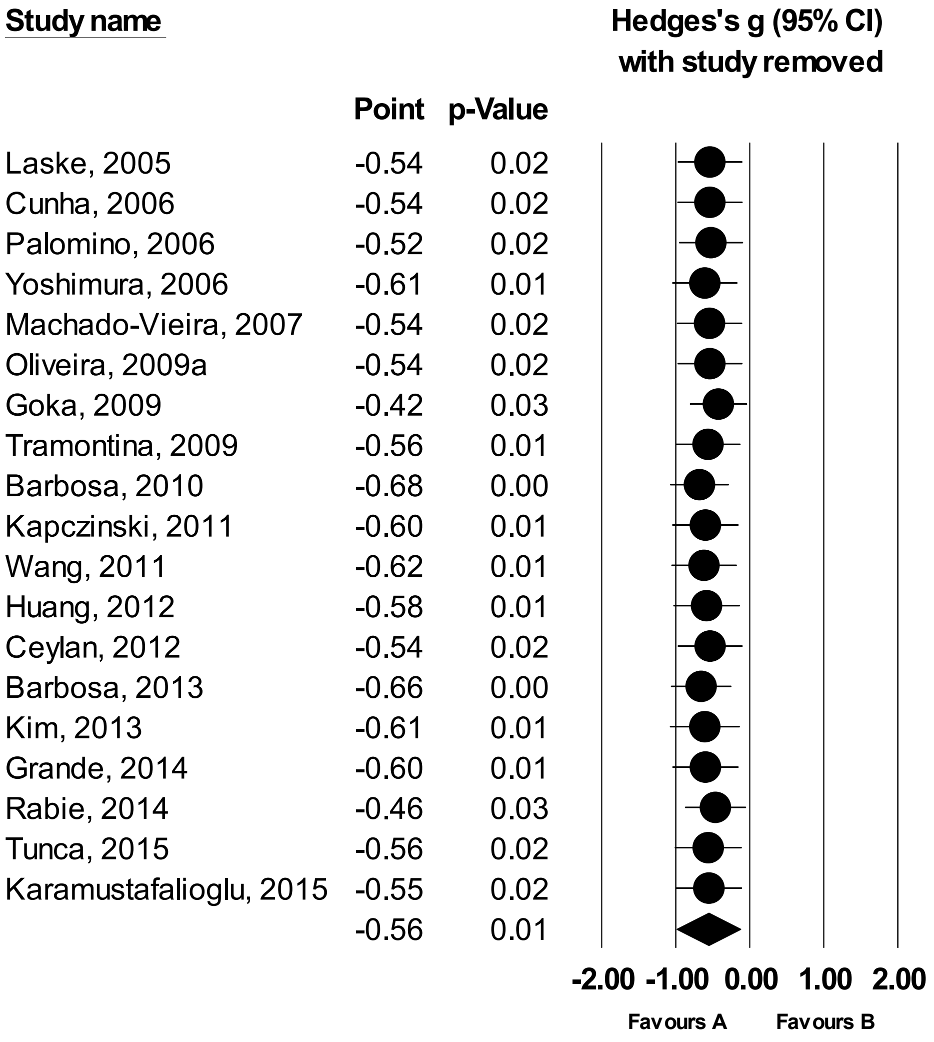

Figure S3. Sensitivity analysis of included studies in between-group meta-analyses of peripheral BDNF levels in participants with bipolar disorder in mania.

Study name	Point	p-Value
Cunha, 2006	-0.97	0.00
Yoshimura, 2006	-0.94	0.00
Mackin, 2007	-1.00	0.00
Oliveira, 2009a	-0.92	0.00
Fernandes, 2009	-0.82	0.00
Dell'Osso, 2010	-0.80	0.00
Kapczinski, 2011	-1.00	0.00
Su, 2011	-0.87	0.00
Magalhaes, 2012	-1.00	0.00
Ceylan, 2012	-0.92	0.00
Panizzutti, 2013	-1.02	0.00
Grande, 2014	-1.03	0.00
Li, 2014	-0.96	0.00
Rabie, 2014	-0.86	0.00
Tunca, 2014	-0.95	0.00
	-0.94	0.00

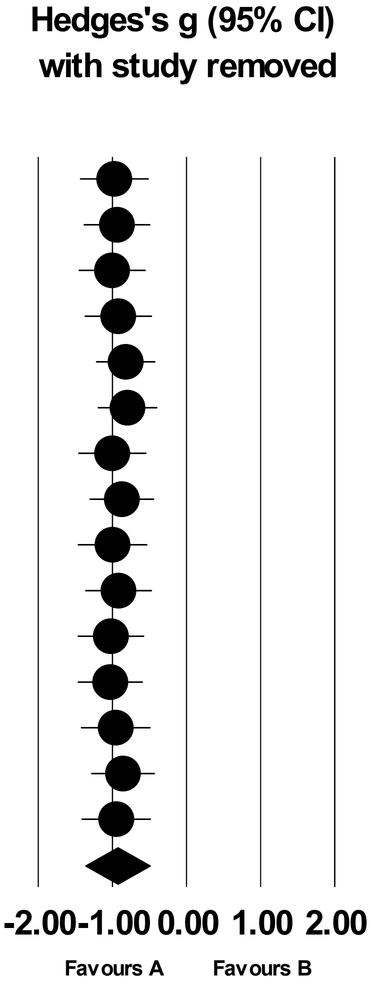

Figure S4. Sensitivity analysis of included studies in between-group meta-analyses of peripheral BDNF levels in participants with bipolar disorder in depression.

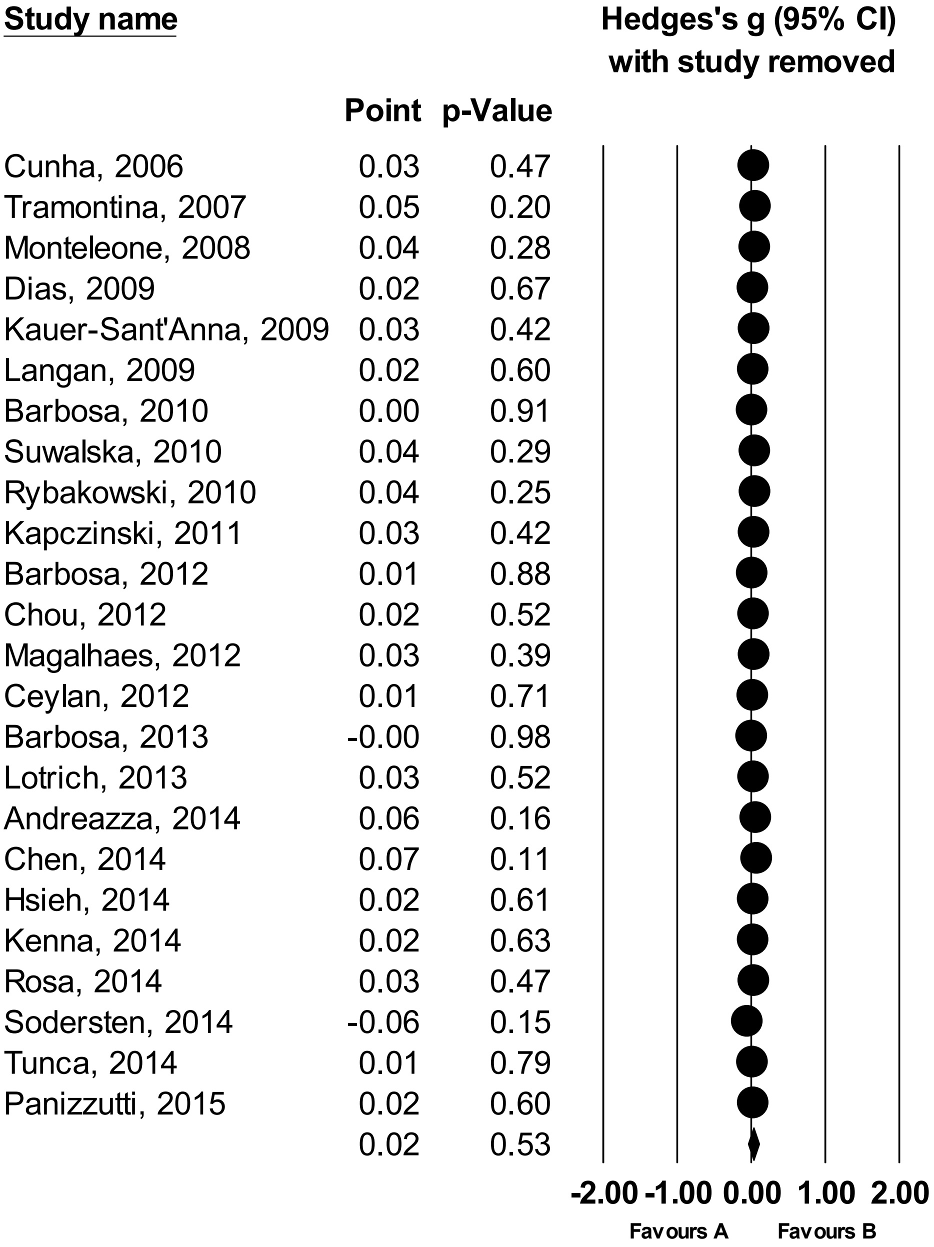

Figure S5. Sensitivity analysis of included studies in between-group meta-analyses of peripheral BDNF levels in participants with bipolar disorder in euthymia.

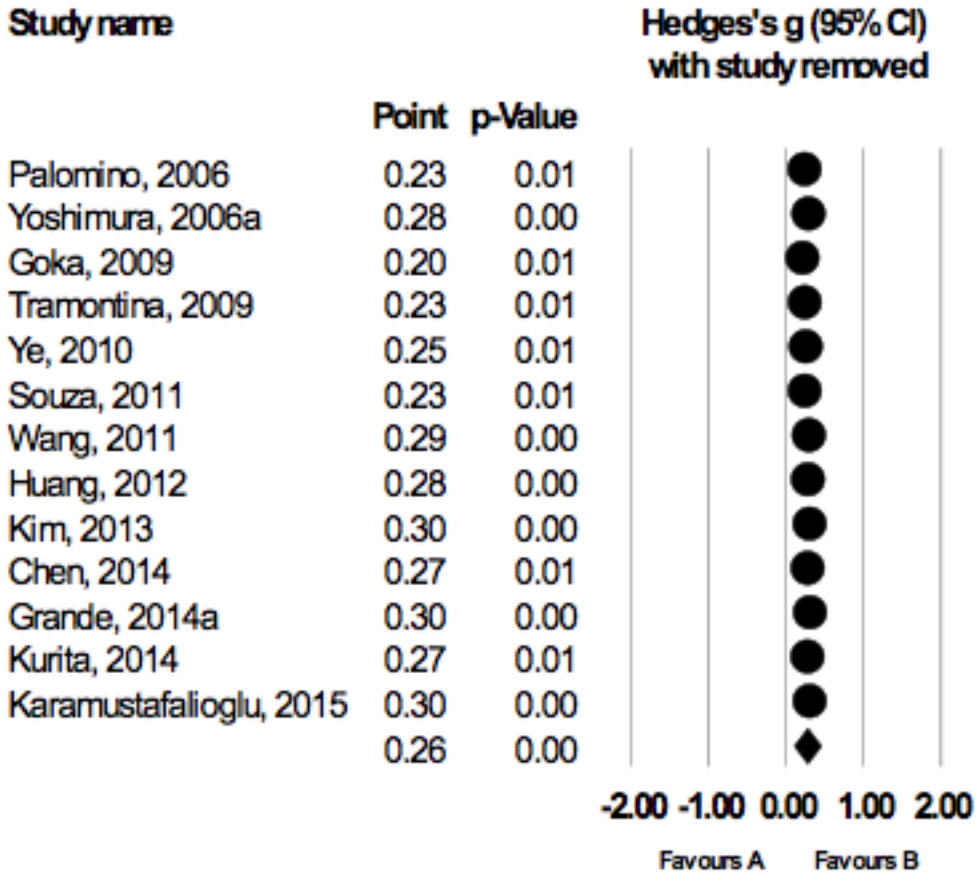

Figure S6. Sensitivity analysis of included studies in within-group meta-analyses of peripheral BDNF levels in participants with bipolar disorder in mania.

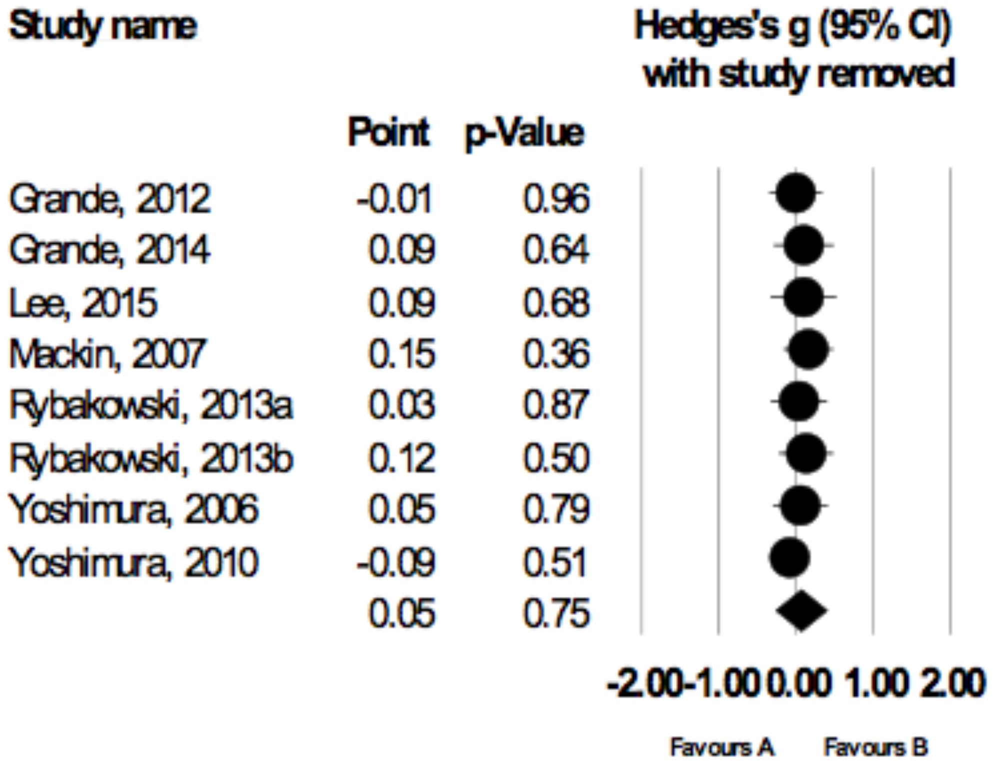

Figure S7. Sensitivity analysis of included studies in within-group meta-analyses of peripheral BDNF levels in participants with bipolar disorder in depression.

Study name	Point	p-Value
Laske, 2005	-1.09	0.01
Cunha, 2006	-1.05	0.00
Palomino, 2006	-1.12	0.00
Yoshimura, 2006	-0.81	0.02
Machado-Vieira, 2007	-0.84	0.00
Oliveira, 2009a	-0.88	0.00
Goka, 2009	-1.19	0.00
Tramontina, 2009	-1.12	0.00
Barbosa, 2010	-0.82	0.06
Kapczinski, 2011	-0.73	0.06
Wang, 2011	-0.63	0.08
Huang, 2012	-0.60	0.06
Ceylan, 2012	-0.63	0.03
Barbosa, 2013	-0.51	0.09
Kim, 2013	-0.46	0.07
Grande, 2014	-0.43	0.07
Rabie, 2014	-0.55	0.03
Tunca, 2015	-0.55	0.02
Karamustafalioglu, 2015	-0.56	0.01
	-0.56	0.01

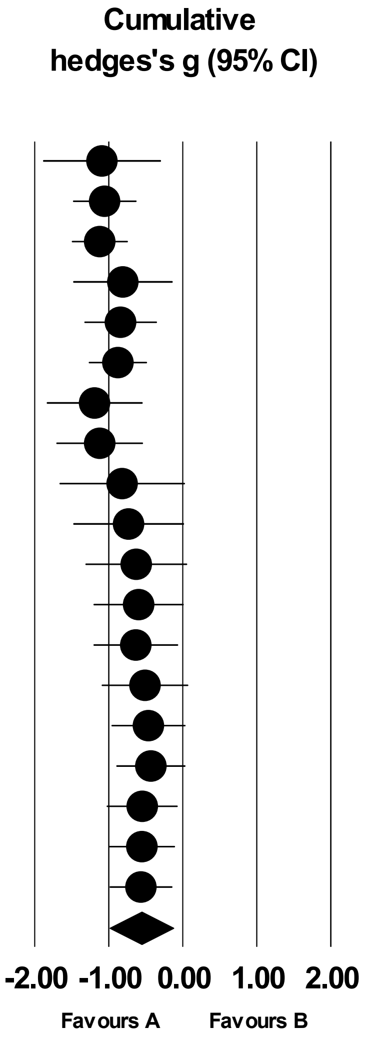

Figure S8. Cumulative Meta-Analysis of included studies in between-group meta-analyses of peripheral BDNF levels in participants with mania.

Study name	Point	p-Value
Cunha, 2006	-0.50	0.07
Yoshimura, 2006	-0.62	0.01
Mackin, 2007	-0.42	0.08
Oliveira, 2009a	-0.64	0.01
Fernandes, 2009	-0.99	0.01
Dell'Osso, 2010	-1.33	0.00
Kapczinski, 2011	-1.15	0.00
Su, 2011	-1.24	0.00
Magalhaes, 2012	-1.11	0.00
Ceylan, 2012	-1.12	0.00
Panizzutti, 2013	-0.99	0.00
Grande, 2014	-0.88	0.00
Li, 2014	-0.86	0.00
Rabie, 2014	-0.95	0.00
Tunca, 2014	-0.94	0.00
	-0.94	0.00

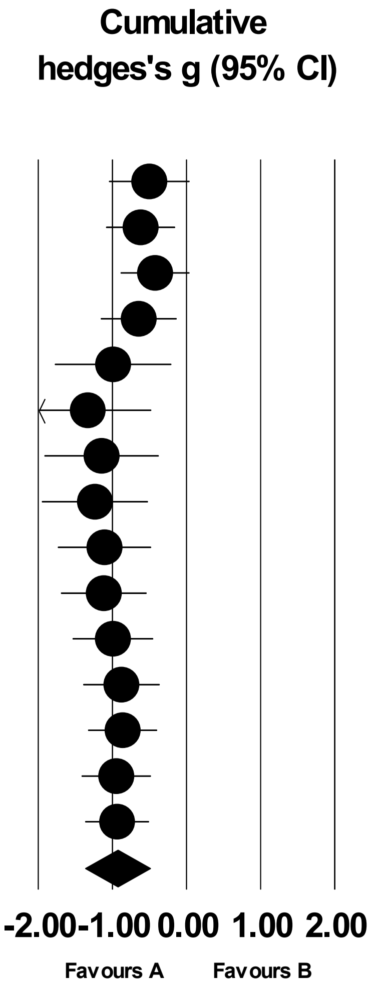

Figure S9. Cumulative Meta-Analysis of included studies in between-group meta-analyses of peripheral BDNF levels in participants with depression.

Study name	Point	p-Value
Cunha, 2006	-0.13	0.60
Tramontina, 2007	-0.22	0.05
Monteleone, 2008	-0.43	0.07
Dias, 2009	-0.27	0.20
Kauer-Sant'Anna, 2009	-0.23	0.14
Langan, 2009	-0.17	0.24
Barbosa, 2010	-0.01	0.97
Suwalska, 2010	-0.04	0.80
Rybakowski, 2010	-0.09	0.54
Kapczinski, 2011	-0.11	0.42
Barbosa, 2012	-0.01	0.96
Chou, 2012	-0.01	0.94
Magalhaes, 2012	-0.05	0.71
Ceylan, 2012	-0.02	0.90
Barbosa, 2013	0.03	0.79
Lotrich, 2013	0.03	0.81
Andreazza, 2014	-0.00	0.99
Chen, 2014	-0.02	0.84
Hsieh, 2014	-0.01	0.93
Kenna, 2014	0.00	0.98
Rosa, 2014	-0.00	0.97
Sodersten, 2014	0.03	0.76
Tunca, 2014	0.05	0.62
Panizzutti, 2015	0.05	0.57
	0.05	0.57

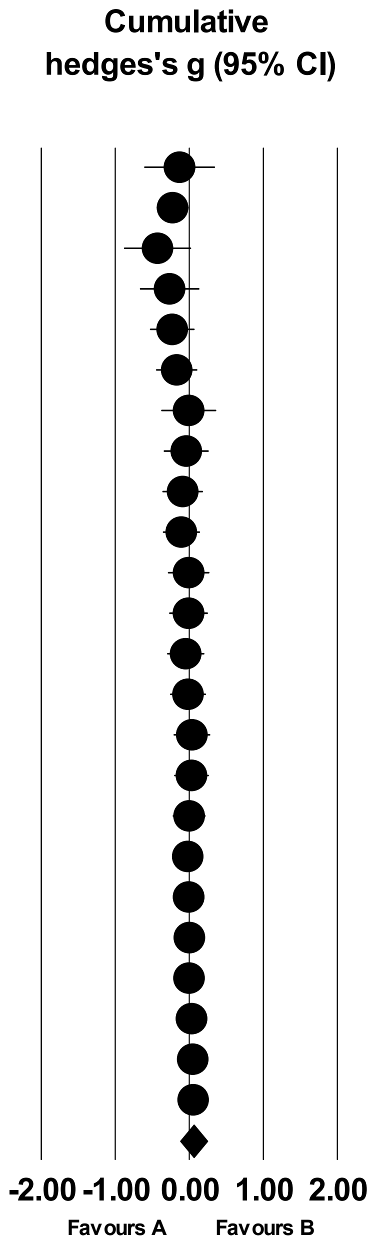

Figure S10. Cumulative Meta-Analysis of included studies in between-group meta-analyses of peripheral BDNF levels in participants with euthymia.

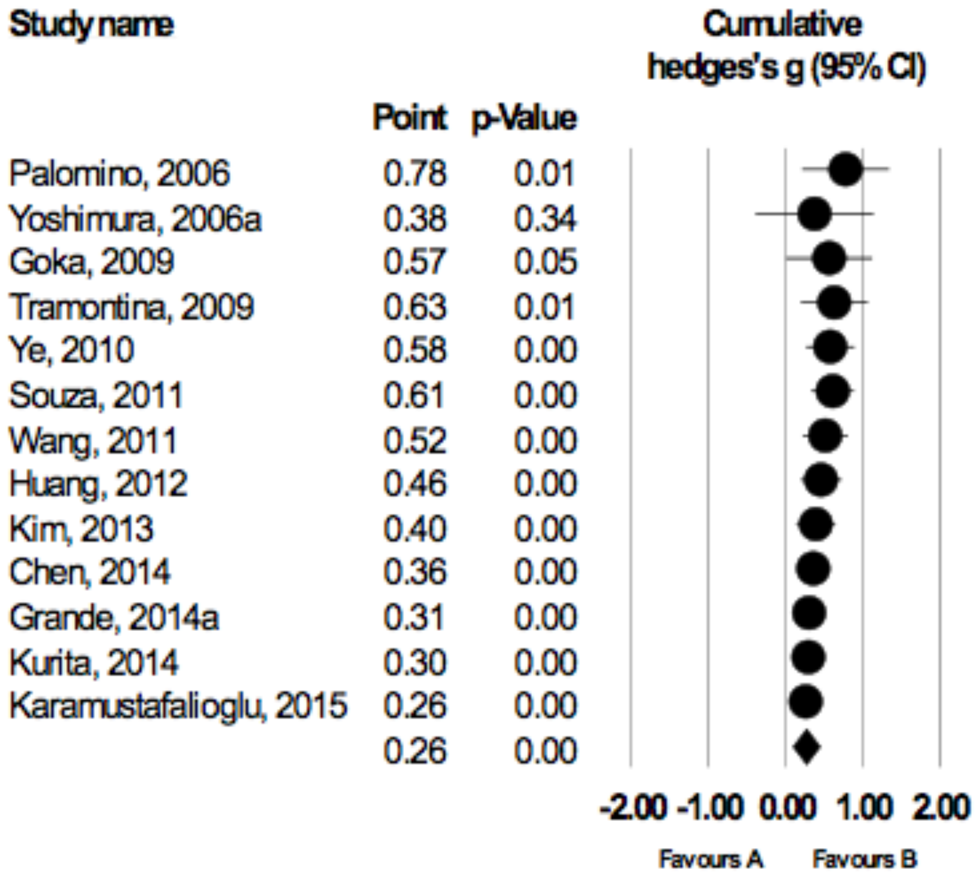

Figure S11. Cumulative Meta-Analysis of included studies in within-group meta-analyses of peripheral BDNF levels in participants with bipolar disorder in mania.

Study name	Point	p-Value
Yoshimura, 2006	0.11	0.75
Mackin, 2007	-0.32	0.41
Yoshimura, 2010	0.23	0.69
Grande, 2012	0.29	0.48
Rybakowski, 2013a	0.27	0.39
Rybakowski, 2013b	0.14	0.60
Grande, 2014	0.09	0.68
Lee, 2015	0.05	0.75
	0.05	0.75

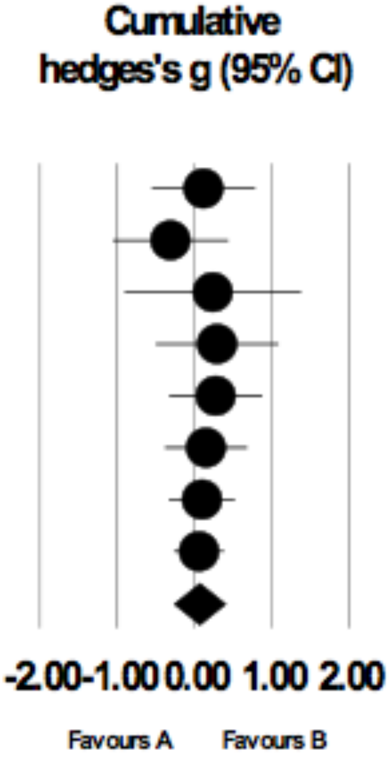

Figure S12. Cumulative Meta-Analysis of included studies in within-group meta-analyses of peripheral BDNF levels in participants with bipolar disorder in depression.

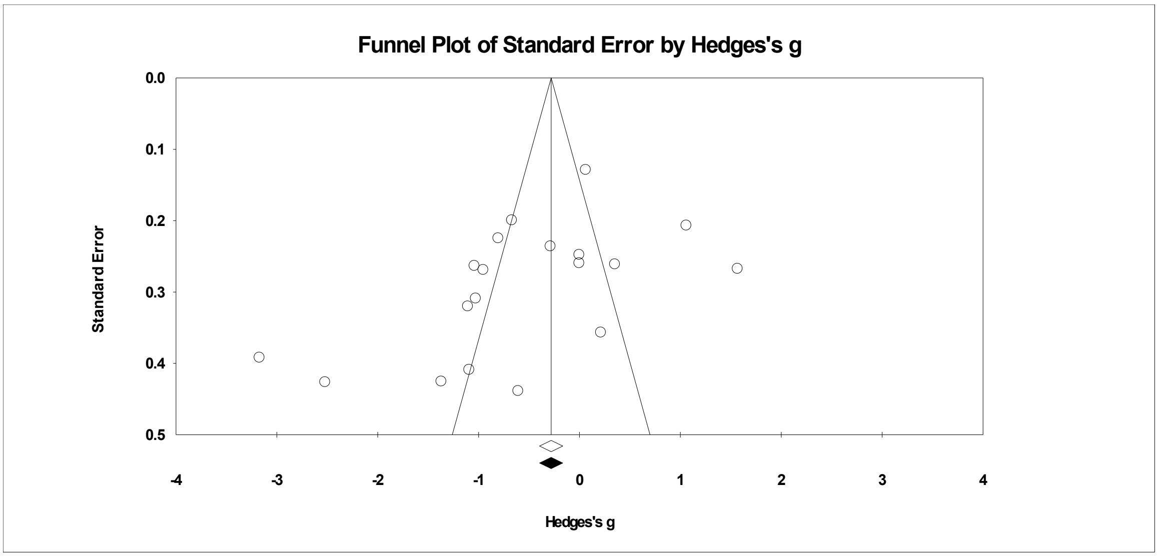

Figure S13. Funnel plot of included studies in between-group meta-analyses of peripheral BDNF levels in participants with bipolar disorder in mania compared to healthy controls. The white diamond shows the observed summary effect size. The black diamond shows the adjusted effect size after the trim and fill procedure.

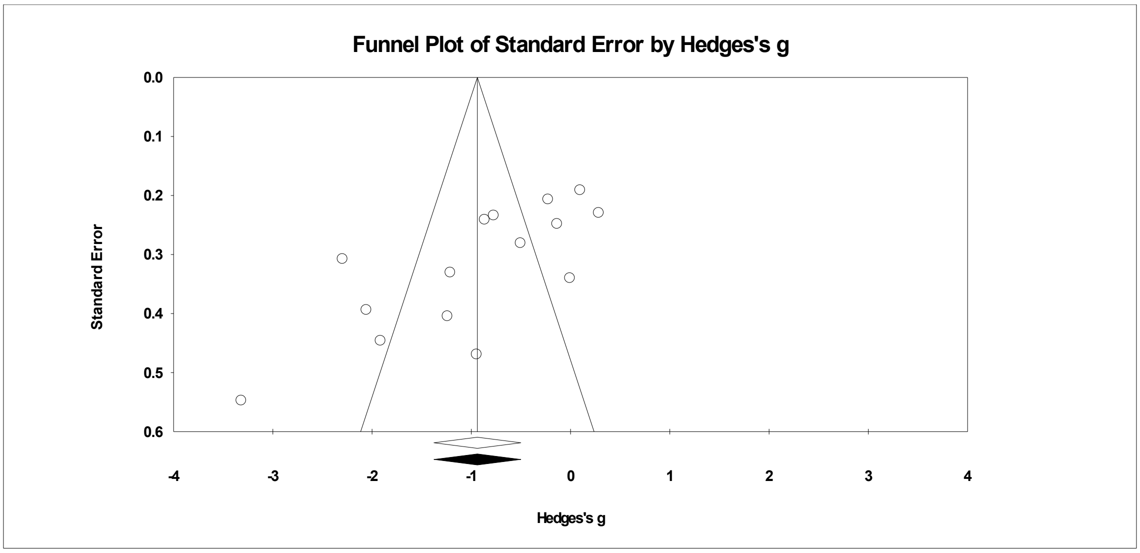

Figure S14. Funnel plot of included studies in between-group meta-analyses of peripheral BDNF levels in participants with bipolar disorder in depression compared to healthy controls.

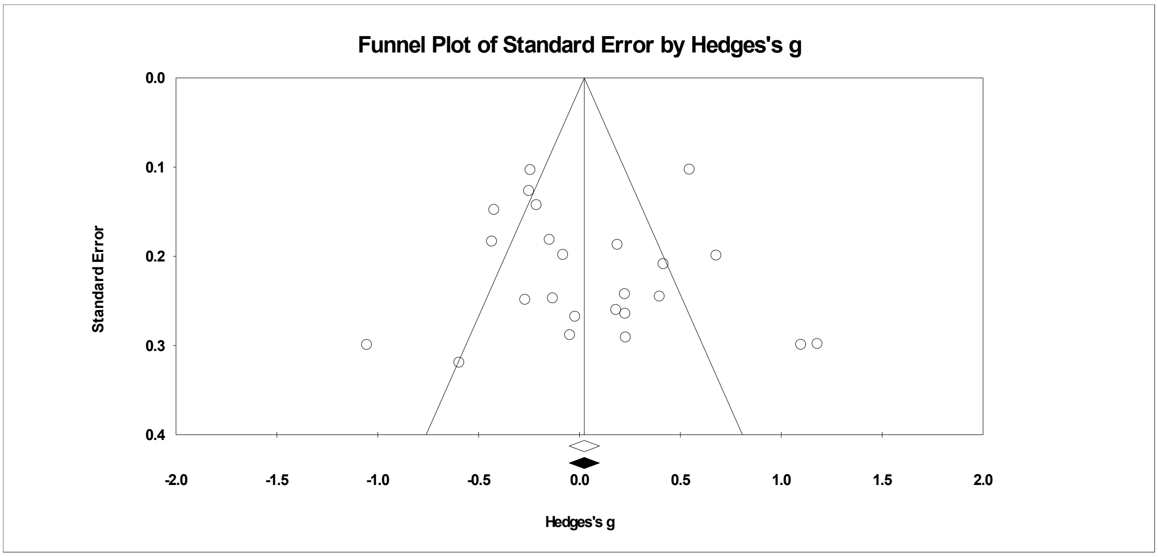

Figure S15. Funnel plot of included studies in between-group meta-analyses of peripheral BDNF levels in participants with bipolar disorder in euthymia compared to healthy controls.

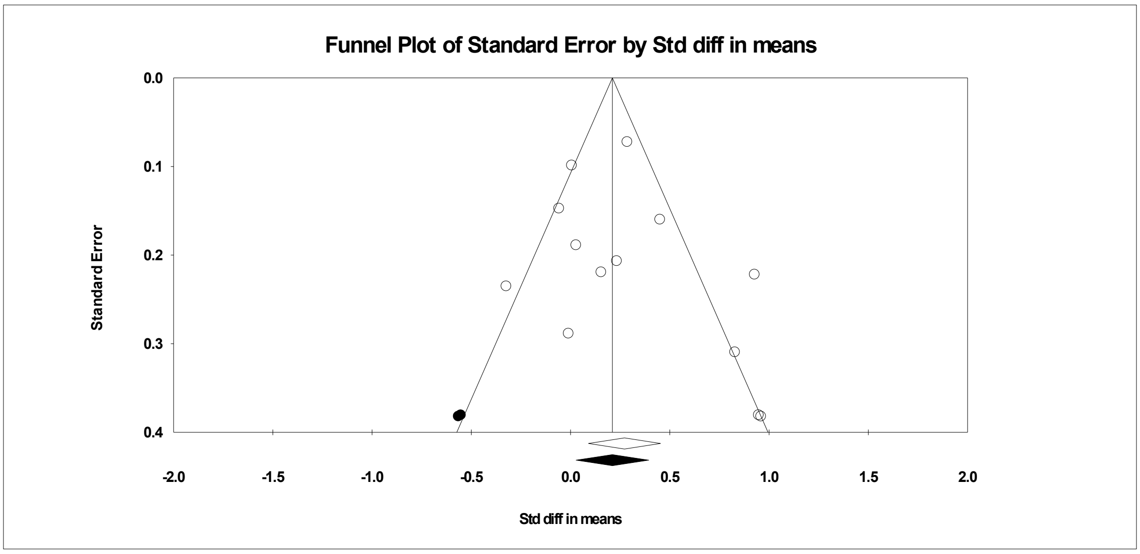

Figure S16. Funnel plot of included studies in within-group meta-analyses of peripheral BDNF level in participants with bipolar disorder in mania before and after treatment.

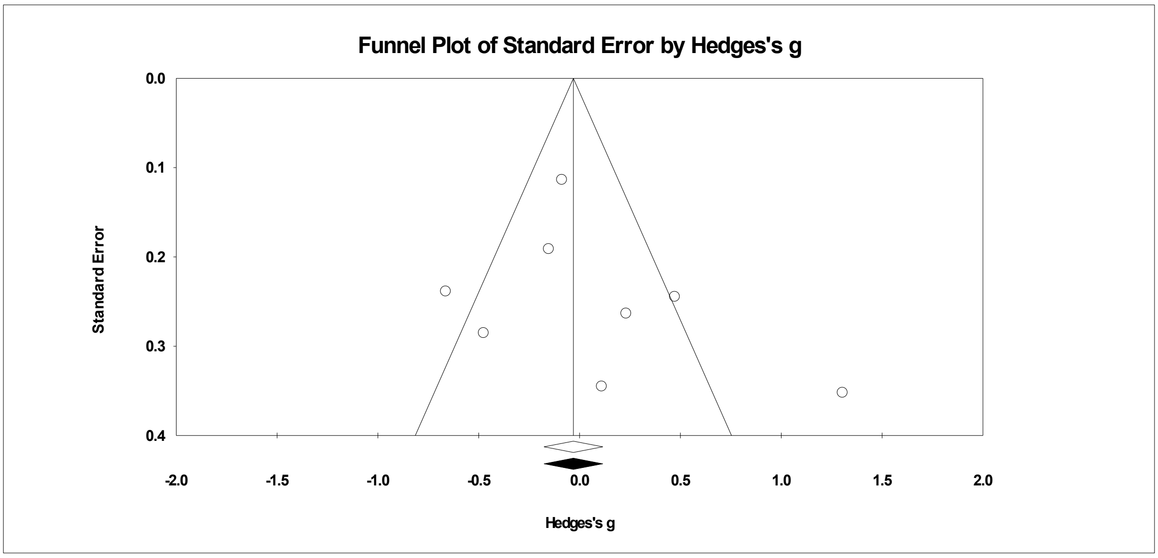

Figure S17. Funnel plot of included studies in within-group meta-analyses of peripheral BDNF levels in participants with bipolar disorder in depression before and after treatment.
