# Supplementary material for: Peripheral brain-derived neurotrophic factor (BDNF) as a biomarker in bipolar disorder: a meta-analysis of 52 studies
Source: BMC Med. 2015 Nov 30;13:289. doi: 10.1186/s12916-015-0529-7 (PMC4666054; doi:10.1186/s12916-015-0529-7)
Supplement: Additional file 2: — Table S1. Studies excluded in secondary screening (with reasons). Table S2. Characteristics of studies included in between-group meta-analyses of peripheral BDNF levels in bipolar disorder, across different mood states. Table S3. Characteristics of studies included in within-group meta-analyses of peripheral BDNF levels in bipolar disorder across different mood states. Table S4. Characteristics of meta-analyses of peripheral BDNF in bipolar disorder. Table S5. Assessment of bias across meta-analyses of peripheral BDNF in bipolar disorder. (PDF 690 kb) [file 12916_2015_529_MOESM2_ESM.pdf]

Table S1. Studies excluded in secondary screening (with reasons).

Ref	Authors (Year)	Reason for exclusion
**S1**	Chatterjee B et al. (2014)	Did not include control group
**S2**	Chatterjee B et al. (2014)	Did not include control group
**S3**	Chen B et al. (2001)	Post mortem study
**S4**	Chen SL et al. (2014)	Meeting abstract; data included in meta-analysis (full-manuscript available)
**S5**	Chou YH et al. (2012)	Meeting abstract; data included in meta-analysis (full-manuscript available)
**S6**	da Graca Cantarelli M et al. (2014)	Did not include control group
**S7**	da Silva PV (2012)	Overlapping data
**S8**	Del Debbio A et al. (2008)	Data unavailable
**S9**	Dell'Osso L et al. (2011)	Overlapping data
**S10**	Duffy A et al. (2014)	Not bipolar disorder; subjects are offspring of bipolar patients
**S11**	Dunham JS et al. (2009)	Postmortem study
**S12**	Ferensztajn E et al. (2014)	Meeting abstract; data included in meta-analysis (full-manuscript available)
**S13**	Ferensztajn E et al. (2014)	Not bipolar disorder; subjects are offspring of bipolar patients
**S14**	Fernandes BS et al. (2010)	Meeting abstract; data included in meta-analysis (full-manuscript available)
**S15**	Filus J et al. (2010)	Did not include control group
**S16**	Gama CS et al. (2007)	Overlapping data
**S17**	Goldstein BI et al. (2011)	Included patients < 18 years
**S18**	Gomes FA et al. (2010)	Not an original article
**S19**	Grande I et al. (2011)	Meeting abstract; data included in meta-analysis (full-manuscript available)
**S20**	Grande I et al. (2012)	Meeting abstract; data included in meta-analysis (full-manuscript available)
**S21**	Grande I et al. (2013)	Meeting abstract; data included in meta-analysis (full-manuscript available)
**S22**	Grande I et al. (2012)	Meeting abstract; data included in meta-analysis (full-manuscript available)
**S23**	Grande I et al. (2014)	Control group included first degree relatives of bipolar patients
**S24**	Grande I et al. (2014)	Duplicated article
**S25**	Gronli O et al. (2009)	Data unavailable
**S26**	Hashimoto K (2014)	Not an original article
**S27**	Hashimoto K (2015)	Not an original article
**S28**	Kapczinski F et al. (2009)	Not an original article
**S29**	Kapczinski F et al. (2008)	Overlapping data
**S30**	Kauer-Sant'Anna M et al. (2010)	Meeting abstract; data included in meta-analysis (full-manuscript available)
**S31**	Kauer-Sant'Anna M (2007)	Did not include a control group

**S32**	Knorr U et al. (2012)	Not bipolar disorder
**S33**	Kunz M et al. (2010)	Meeting abstract; data included in meta-analysis (full-manuscript available)
**S34**	Lahera G et al. (2014)	Insufficient data for meta-analysis
**S35**	Lee SY et al. (2015)	Duplicated article
**S36**	Licinio J et al. (2002)	Not an original article
**S37**	Licinio J et al. (2010)	Not an original article
**S38**	Machado-Vieira R et al. (2009)	Not bipolar disorder
**S39**	Mesman E et al. (2015)	Not bipolar disorder; subjects are offspring of bipolar patients
**S40**	Moreira L et al. (2011)	Not an original article
**S41**	Munkholm K et al. (2014)	Double-counting of patients (Multiple blood samples)
**S42**	Nair A et al. (2006)	Not an original article
**S43**	Permoda-Osip A et al. (2011)	Meeting abstract; data included in meta-analysis (full-manuscript available)
**S44**	Piccinni A et al. (2009)	Not bipolar disorder; data unavailable
**S45**	Post RM (2007)	Not an original article
**S46**	Rakofsky JJ et al. (2012)	Not an original article
**S47**	Rybakowski JK (2014)	Not an original article
**S48**	Scola G et al. (2015)	Not an original article
**S49**	Sousa RT et al. (2011)	Duplicated article
**S50**	Suwalska A et al. (2008)	Meeting abstract; data included in meta-analysis (full-manuscript available)
**S51**	Teixeira AL et al. (2010)	Not an original article
**S52**	Teixeira AL et al. (2011)	Meeting abstract; data included in meta-analysis (full-manuscript available)
**S53**	Terao T (2008)	Not an original article
**S54**	Trajkovska V et al. (2008)	Did not include a control group
**S55**	Tsai SJ (2004)	Not an original article
**S56**	Vasconcelos-Moreno MP et al. (2012)	Not bipolar disorder; subjects are offspring of bipolar patients
**S57**	Videira Dias V et al. (2009)	Data unavailable
**S58**	Vinberg M et al. (2009)	Did not include a control group
**S59**	Yalcin Arslan Y et al. (2013)	Overlapping data
**S60**	Yatham LN et al. (2009)	Overlapping data
**S61**	Yuan TF (2008)	Not an original article
**S62**	Zhang C et al. (2014)	Meeting abstract; data included in meta-analysis (full-manuscript available)

Table S2. Characteristics of studies included in between-group meta-analyses of peripheral BDNF levels in bipolar disorder, across different mood states.

A) Bipolar Depression

Ref	Author (Year)	Sample Size (Patients/ Controls)	Age (mean ± SD) % Females		BDNF Unit	BDNF (mean ± SD)		Baseline Depression Scores	Sample Source (Type of Assay)	Manufacturer of the Test
			Patients	Controls		Patients	Controls			
S63	Ceylan (2012)	8 / 37	45.5 ± 10.8<br>37.5	35.2 ± 11.7<br>64.9	pg/mL	3984.3 ± 606.1	7050.7 ± 2644.3	26.5 ± 2.6	Serum (ELISA)	Chemikine
S64	Cunha (2006)	21 / 32	40.7 ± 9.2<br>71.4	40.7 ± 12.1<br>65.6	pg/µg	0.15 ± 0.13	0.20 ± 0.07	22.8 ± 4.4	Serum (ELISA)	Chemicon
S65	de Oliveira (2009)	20 / 22	35.0 ± 11.2<br>90.0	35.2 ± 8.1<br>90.0	pg/µL	0.22 ± 0.17	0.40 ± 0.12	21.4 ± 7.7	Serum (ELISA)	Chemicon
S66	Dell'Osso (2010)	16 / 15	46.7 ± 17.7<br>50.0	46.9 ± 9.2<br>80.0	pg/mL	2119.0 ± 1339.8	5400.0 ± 2.3	27.8 ± 5.3	Plasma (ELISA)	Promega
S67	Fernandes (2009)	40 / 30	41.3 ± 8.4<br>55.0	41.0 ± 12.0<br>60.0	pg/µg	0.15 ± 0.08	0.38 ± 0.12	23.4 ± 7.5	Serum (ELISA)	Chemicon
S68	Grande (2014)	27 / 62	36.3 ± 11.3<br>74.1	34.2 ± 9.4<br>81.3	ng/mL	42.9 ± 25.5	36.4 ± 21.2	22.1 ± 6.6	Serum (ELISA)	Millipore
S69	Kapczinski (2011)	20 / 80	46.1 ± 9.3<br>80.0	40.7 ± 12.5<br>60.0	pg/µg	0.12 ± 0.05	0.13 ± 0.09	24.3 ± 8.2	Serum (ELISA)	Chemicon
S70	Li (2014)	21 / 167	32.2 ± 4.1<br>71.4	30.9 ± 4.5<br>79.0	pg/mL	2.7 ± 0.5	3.0 ± 0.4	22.2 ± 1.7	Plasma (ELISA)	R&D Systems
S71	Mackin (2007)	20 / 14	48.6 ± 10.8<br>5.0	43.7 ± 12.9<br>14.3	pg/mL	13755.2 ± 7932.2	13800.4 ± 9107.0	18.1 ± 9.9	Serum (ELISA)	Promega
S72	Magalhaes (2012)	31 / 92	21.7 ± 2.4<br>74.2	22.4 ± 2.7<br>57.4	pg/µg	0.24 ± 0.11	0.27 ± 0.15	N/A	N/A (N/A)	N/A
S73	Panizzutti (2013)	54 / 54	50<br>75.9	48<br>72.0	ng/mL	25.9 ± 8.5	25.1 ± 7.7	21.0	Serum (ELISA)	N/A
S74	Rabie (2014)	25 / 15	32.1 ± 8.3<br>60.0	30.5 ± 5.0<br>46.7	pg/mL	23.3 ± 11.1	42.2 ± 12.9	21 ± 5.6	Serum (ELISA)	Quantikine
S75	Su (2011)	10 / 21	22.7 ± 2.9<br>0.0	25.0 ± 3.0<br>0.0	ng/mL	5.4 ± 4.7	12.5 ± 3.0	N/A	Plasma (Other)	Biomedicines
S76	Tunca (2014)	26 / 61	42.5 ± 10.5<br>32.1	38.3 ± 11.6<br>56.7	pg/mL	3639.6 ± 1395.1	5646.2 ± 2587.0	24.0 ± 5.7	Serum (ELISA)	Chemikine

S77	Yoshimura (2006)	6 / 20	N/A<br>N/A	30.0 ± 11.0<br>55.0	pg/mL	16.0 ± 8.1	26.2 ± 11.0	24.0 ± 6.0	Serum (ELISA)	Promega

B) Euthymia

Ref	Author (Year)	Sample Size (Patients/ Controls)	Age (mean ± SD) % Females		BDNF Unit	BDNF (mean ± SD)		Sample Source (Type of Assay)	Manufacturer of the Test
			Patients	Controls		Patients	Controls		
S78	Andreazza (2014)	76 / 118	64.0 ± 9.7<br>N/A	65.9 ± 9.6<br>N/A	pg/µg	8.95 ± 6.22	12.29 ± 8.81	N/A	N/A
S79	Barbosa (2010)	19 / 38	44.5 ± 10.9<br>57.9	42.9 ± 9.7<br>52.6	pg/mL	2695.8 ± 1570.1	1211.0 ± 1043.4	Plasma (ELISA)	DuoSet, R&D Systems
S80	Barbosa (2012)	25 / 25	50.9 ± 9.1<br>68.0	48.0 ± 7.1<br>56.0	pg/mL	3901.0 ± 2357.1	1754.4 ± 1354.9	Plasma (ELISA)	DuoSet, R&D Systems
S81	Barbosa (2013)	48 / 58	47.8 ± 10.4<br>74.3	46.8 ± 9.4<br>72.4	pg/mL	2649.1 ± 1861.0	1615.8 ± 1144.0	Plasma (ELISA)	DuoSet, R&D Systems
S63	Ceylan (2012)	30 / 37	37.3 ± 10.8<br>60.0	35.2 ± 11.7<br>64.9	pg/mL	8041.5 ± 2205. 6	7050.7 ± 2644.3	Serum (ELISA)	Chemikine
S82	Chen (2014)	367 / 126	31.7 ± 11.2<br>48.8	32.4 ± 7.5<br>38.8	ng/mL	14.9 ± 10.00	17.3 ± 9.1	Plasma (ELISA)	Quantikine
S83	Chou (2012)	23 / 33	36.5 ± 8.9<br>73.9	37.6 ± 7.8<br>63.6	pg/mL	328.0 ± 242.4	334.5 ± 343.6	Plasma (ELISA)	Promega
S64	Cunha (2006)	32 / 32	40.3 ± 12.0<br>62.5	40.7 ± 12.1<br>65.6	pg/µg	0.19 ± 0.08	0.20 ± 0.07	Serum (ELISA)	Chemicon
S84	Dias (2009)	65 / 50	37.8 ± 10.5<br>63.1	33.6 ± 9.7<br>72.0	pg/µg	0.28 ± 0.21	0.24 ± 0.21	Serum (RIA)	Promega
S85	Hsieh (2014)	28 / 28	36.6 ± 8.1<br>67.9	36.6 ± 7.9<br>67.9	pg/mL	586.0 ± 758.2	445.4 ± 408.0	Serum (ELISA)	N/A
S69	Kapczinski (2011)	20 / 80	46.6 ± 12.7<br>60.0	40.7 ± 12.5<br>60.0	pg/µg	0.11 ± 0.01	0.13 ± 0.01	Serum (ELISA)	Chemicon
S86	Kauer-Sant'Anna (2009)	60 / 60	31.9 ± 11.6<br>63.3	32.6 ± 11.8<br>65.0	pg/mL	0.6 ± 0.3	0.6 ± 0.3	Serum (ELISA)	Chemicon
S87	Kenna (2014)	26 / 47	32.9 ± 6.4<br>100.0	31.8 ± 6.4<br>100.0	ng/mL	53.8 ± 52.6	43.5 ± 40.8	Plasma (ELISA)	Promega

S88	Langan<br>(2009)	24 / 22	40.7 ± 9.0<br>41.6	40.4 ± 7.9<br>54.5	ng/mL	35.9 ± 8.2	33.5 ± 11.9	Serum<br>(N/A)	N/A
S89	Lotrich<br>(2013)	21/26	64.8 ±9.1<br>53.9	65.5 ± 8.4<br>61.9	N/A	21.3 ± 5.4	21.6 ± 7.1	Serum<br>(ELISA)	R&D Systems
S72	Magalhaes<br>(2012)	11 / 92	21.8 ± 2.4<br>72.7	22.4 ± 2.7<br>57.4	pg/µg	0.19 ± 0.07	0.27 ± 0.15	N/A	N/A
S90	Monteleone<br>(2008)	28 / 22	45.1 ± 11.2<br>60.7	40.1 ± 16.4<br>63.6	pg/mL	27.8 ± 14.7	42.6 ± 12.7	Serum<br>(RIA)	Promega
S91	Panizzutti<br>(2014)	31/27	46.4 ± 12.9<br>74.2	45.9 ± 6.8<br>74.1	ng/mL	27.9 ± 7.8	26.5 ± 7.3	Serum<br>(ELISA)	Chemicon
S92	Rosa<br>(2014)	50 / 50	41.3 ± 13.4<br>56.0	41.6 ± 13.6<br>56.0	ng/mL	1.6 ± 0.8	1.6 ± 0.8	Plasma<br>(ELISA)	R&D Systems
S93	Rybakowski<br>(2010)	60/60	52.6 ± 10.2<br>58.3	52.1 ± 13.6<br>58.3	ng/mL	23.6 ± 13.3	28.9 ± 10.9	Plasma<br>(ELISA)	Quantikine
S94	Sodersten<br>(2014)	262 / 155	39.0 ± 12.6<br>63.1	37.8 ± 14.6<br>56.8	ng/mL	29.8 ± 7.9	25.3 ± 8.6	Serum<br>(ELISA)	Adipo<br>Bioscience
S95	Suwalska<br>(2010)	141 / 75	53.7 ± 12.7<br>63.8	55.7 ± 11.7<br>69.3	ng/mL	24.2 ± 17.0	27.4 ± 10.4	Serum<br>(ELISA)	Quantikine
S96	Tramontina<br>(2007)	114/137	42.5 ± 11.5<br>71.9	44.1 ± 13.8<br>67.1	pg/µg	0.14 ± 0.08	0.16 ± 0.08	Serum<br>(ELISA)	Chemicon
S76	Tunca<br>(2014)	37 / 61	36.2 ± 10.0<br>62.2	38.3 ± 11.6<br>56.7	pg/mL	6851.0 ± 3275.9	5646.2 ± 2587.0	Serum<br>(ELISA)	Chemikine

C) (Hypo)mania

Ref	Author (Year)	Sample Size (Patients/ Controls)	Age (mean ± SD) % of Females		BDNF Unit	BDNF (mean ± SD)		Baseline YMRS Scores	Sample Source (Type of Assay)	Manufacturer of the Test
			Patients	Controls		Patients	Controls			
S79	Barbosa<br>(2010)	34 / 38	49.6 ± 14.2<br>61.8	42.9 ± 9.7<br>52.6	pg/mL	3161.1 ±<br>1409.3	1211.0 ±<br>1043.4	28.5 ± 6.2	Plasma<br>(ELISA)	DuoSet, R&D<br>Systems
S81	Barbosa<br>(2013)	48 / 58	51.0 ± 13.7<br>62.5	46.8 ± 9.4<br>72.4	pg/mL	3075.3 ±<br>1592.6	1615.8 ±<br>1144.0	25.2 ± 7.9	Plasma<br>(ELISA)	DuoSet, R&D<br>Systems
S63	Ceylan	15 / 37	37.7 ± 11.1	35.2 ± 11.7	pg/mL	4422.7 ±	7050.7 ±	30.1 ± 11.8	Serum	Chemikine

	(2012)		66.7	64.9		1279.7	2644.3		(ELISA)	
S64	Cunha<br>(2006)	32 / 32	40.1 ± 12.6<br>43.8	40.7 ± 12.1<br>65.6	pg/µg	0.14 ± 0.04	0.20 ± 0.07	34.5 ± 7.1	Serum<br>(ELISA)	Chemicon
S65	de Oliveira<br>(2009)	24 / 22	41.8 ± 13.5<br>66.6	35.2 ± 8.1<br>66.6	pg/µL	0.28 ± 0.11	0.40 ± 0.12	32.2 ± 10.1	Serum<br>(ELISA)	Chemicon
S97	Goka<br>(2009)	29 / 29	32.6 ± 8.3<br>48.3	33.8 ± 6.6<br>48.3	pg/mL	14.3 ± 5.8	40.2 ± 9.7	35.9 ± 9.4	Serum<br>(ELISA)	Quantikine
S68	Grande<br>(2014)	19 / 62	36.3 ± 13.2<br>36.8	36.7 ± 13.3<br>22.2	ng/mL	36.4 ± 30.5	36.4 ± 21.2	26.0 ± 9.4	Serum<br>(ELISA)	Millipore
S98	Huang<br>(2012)	26 / 56	33.2 ± 11.7<br>53.8	32.5 ± 5.7<br>64.3	ng/mL	4.2 ± 4.0	6.7 ± 10.1	42.1 ± 7.5	Serum<br>(RIA)	Promega
S69	Kapczinski<br>(2011)	20 / 80	37.9 ± 12.1<br>60.0	40.7 ± 12.5<br>60.0	pg/µg	0.13 ± 0.01	0.13 ± 0.01	30.1 ± 6.9	Serum<br>(ELISA)	Chemicon
S99	Karamustafalio-<br>glu (2015)	68/30	34.7 ± 10.6<br>0	30.9 ± 7.6<br>0	ng/ml	2.4 ± 2.3	4.9 ± 4.4	45.1 ± 7.7	Plasma<br>(ELISA)	N/A
S100	Kim<br>(2013)	116 / 123	35.9 ± 11.7<br>63.8	35.4 ± 10.4<br>54.6	pg/mL	976.9 ± 904.8	927.6 ± 609.4	34.0 ± 9.6	Whole Blood<br>(ELISA)	DuoSet, R&D Systems
S101	Laske<br>(2005)	8/30	50.9 ± N/A<br>33.3	58.1 ± N/A<br>73.3	ng/ml	15.7 ± 3.0	21.3 ± 5.4		Serum<br>(ELISA)	DuoSet, R&D Systems
S102	Machado-Vieira<br>(2007)	30 / 30	26.0 ± 4.0<br>76.7	26.5 ± 5.2<br>76.7	pg/mL	224.8 ± 76.5	318.5 ± 114.2	36.9 ± 0.5	Plasma<br>(ELISA)	Chemicon
S103	Palomino<br>(2006)	14 / 12	23.7 ± 6.9<br>N/A	25.5 ± 5.2<br>N/A	ng/mL	3.8 ± 2.0	7.9 ± 3.7	N/A	Plasma<br>(ELISA)	Chemicon
S74	Rabie<br>(2014)	25 / 15	29.7 ± 6.7<br>36.0	30.5 ± 5.0<br>46.7	pg/mL	19000 ±<br>5657.0	42200 ± 1298	48 ± 3.86	Serum<br>(ELISA)	Quantikine
S104	Tramontina<br>(2009)	10 / 10	34.9 ± 13.8<br>50.0	34.3 ± 4.0<br>50.0	pg/µg	0.26 ± 0.10	0.31 ± 0.05	26.2 ± 9.5	Serum<br>(ELISA)	Chemicon
S76	Tunca<br>(2014)	33 / 61	36.8 ± 12.0<br>65.7	38.3 ± 11.6<br>56.7	pg/mL	4129.2 ±<br>1704.0	5646.2 ±<br>2587.0	30.4 ± 11.1	Serum<br>(ELISA)	Chemikine
S105	Tunca<br>(2015)	39 / 78	36.6 ± 11.7<br>66.7	38.2 ± 11.5<br>51.3	pg/mL	3743.9 ±<br>2113.6	5457.2 ±<br>2734.3	31.6 ± 11.1	Serum<br>(ELISA)	ChemiKine
S106	Wang<br>(2011)	28 / 30	37.5 ± 13.6<br>60.7	36.9 ± 6.3<br>56.6	pg/mL	1194.9 ±<br>2214.6	639.9 ± 257.4	31.2 ± 5.0	Whole Blood<br>(ELISA)	N/A

S77	Yoshimura (2006)	12 / 20	34.1 ± 15.0 N/A	30.0 ± 11.0 55.0	pg/mL	26.2 ± 11.0	24.2 ± 7.9	22.0 ± 5.0	Plasma (ELISA)	Promega

D) Mixed Episode

Ref	Author (Year)	Sample Size (Patients/ Controls)	Age (mean + SD) % Females		BDNF Unit	BDNF (mean + SD)		Baseline YMRS (HDRS) Scores	Sample Source (Type of Assay)	Manufacturer of the Test
			Patients	Controls		Patients	Controls			
S68	Grande (2014)	15 / 62	40.3 ± 10.4 75.0	37.2 ± 10.6 73.3	ng/mL	44.2 ± 34.9	36.4 ± 21.2	19.9 ± 8.3 (18.3 ± 8.9)	Serum (ELISA)	Millipore
S72	Magalhaes (2012)	10 / 92	21.6 ± 2.0 70.0	22.4 ± 2.7 57.4	pg/µg	0.30 ± 0.11	0.27 ± 0.15	N/A	N/A	N/A
S107	Piccini (2015)†	19 / 15	38.2 ± 10.0 47.4	36.9 ± 9.2 80.0	ng/mL	3.3 ± 2.6	5.4 ± 2.3	N/A (6.0 ± 0.2†)	Plasma (ELISA)	Promega

† Applied CGI-S, instead of HDRS

Table S3. Characteristics of studies included in within-group meta-analyses of peripheral BDNF levels in bipolar disorder across different mood states.

A) Bipolar Depression

Ref	Author (Year)	Sample Size (Baseline/ Final)	Age (mean ± SD) % Females	BDNF Unit	BDNF (mean ± SD) Baseline	BDNF (mean ± SD) Post-Treatment	Baseline HDRS Scores (Change)	Intervention (Length of follou-up)	Source, Manufacturer of the Test
S108	Grande (2012)	17 / 17	38.7 ± 11.7 77.0	ng/mL	49.3 ± 11.6	57.0 ± 17.6	21.6 ± 5.6 (-9.3)	Quetiapine ER (16 weeks)	Serum, Millipore
S68	Grande (2014)	27 / 26	36.3 ± 11.3 74.1	ng/mL	42.9 ± 25.5	39.4 ± 16.1	22.1 ± 6.6 (-14.2)	Quetiapine ER or TAU (16 weeks)	Serum, Millipore
S109	Lee (2015)	117 / 76	30.7 ± 11.1 44.4	ng/mL	17.6 ± 8.5	16.8 ± 9.7	19.2 ± 5.4 (-9.8)	Valproate (12 weeks)	Plasma, Quantikine
S71	Mackin (2007)	20 / 20	48.6 ± 10.8 5.0	pg/mL	13755.2 ± 7932.2	9001.7 ± 4511.5	18.1 (N/A)	Mifepristone (1 week)	Serum, Promega
S110	Rybakowski (2013) Responders	13 / 13	49.0 ± 11.0 84.0	ng/mL	38071 ± 9711	40402 ±9038	21.0 ± 5.0 (-17.0)	Ketamine (2 weeks)	Serum, R&D Systems
S110	Rybakowski (2013) Non-responders	12 / 12	49.0 ± 11.0 84.0	ng/mL	42387 ± 7414	38904 ±6024	21.0 ± 5.0 (-2.0)	Ketamine (2 weeks)	Serum, R&D Systems
S77	Yoshimura (2006)	6 / 6	N/A	ng/mL	16.0 ± 8.1	17.0 ± 7.0	24.0 ± 6.0 (-5.0)	Risperidone (4 weeks)	Plasma, Promega
S111	Yoshimura (2010)	14/14	N/A	ng/mL	1.1 ± 0.8	2.1 ± 0.6	19.0 ± 2.5 (-8.6)	Atypical antipsychotics (4 weeks)	Plasma, Promega

Abbreviation: TAU = treatment as usual; **ER** = extended-release.

B) (Hypo)mania

Ref	Author (Year)	Sample Size (Baseline/ Final)	Age (mean ± SD) % Females	BDNF Unit	BDNF (mean ± SD)		Baseline YMRS Scores (Change)	Intervention (Length of follou-up)	Manufacturer of the Test
					Baseline	Post-Treatment			
S82	Chen (2014)	309/198	31.4 ± 11.2 47.2	ng/mL	15.0 ± 10.7	17.9 ± 9.4	11.3 ± 6.0 (-6.1)	Valproate + Dextromethorphan (12 weeks)	Plasma, Quantikine
S112	de Sousa (2011)	10 / 10	25.4 ± 7.5 40.0	pg/mL	406.3 ± 69.5	510.9 ± 127.1	37.3 ± 9.5 (-26.6)	Lithium (4 weeks)	Plasma, Chemicon
S97	Goka (2009)	29 / 29	32.6 ± 8.3 48.3	pg/mL	14.3 ± 5.8	20.5 ± 7.3	35.9 ± 9.4 (-30.7)	Multiple drugs (4 weeks)	Serum, Quantikine
S68	Grande (2014)	19 / 19	36.3 ± 13.2 36.8	ng/mL	36.4 ± 30.5	27.8 ± 11.7	26.0 ± 9.4 (-21.7)	Quetiapine ER or TAU (16 weeks)	Serum, Millipore
S98	Huang (2012)	26 / 21	31.7 ± 10.6 61.9	ng/mL	4.0 ± 4.0	4.6 ± 3.7	42.1 ± 7.5 (-36.2)	Lithium, valproate, various antipsychotics (4 weeks)	Serum Promega
S99	Karamustafalioglu (2015)	46 / 46	N/A 0.0	ng/mL	2.2 ± 1.8	2.1 ± 1.7	43.7 ± 5.8 (-40.7)	Haloperidol + quetiapine (4 weeks)	Plasma, N/A
S100	Kim (2013)	116 / 102	36.0 ± 11.7 62.7	pg/mL	976.9 ± 904.8	983.1 ± 770.3	34.0 ± 9.6 (-28.7)	Multiple drugs (6 weeks)	Whole blood, DuoSet
S113	Kurita (2014)	24/24	54.5 ± 16.0 45.8	pg/mL	5493 ± 4905	6619 ± 4709	32.5 ± 8.3 (-22.5)	Lithium, valproate, various antipsychotics (6 weeks)	Plasma, N/A
S103	Palomino (2006)	14 / 14	N/A	ng/mL	3.8 ± 2.0	5.7 ± 2.5	N/A	Lithium, mood stabilizers, atypical antipsychotics (12 weeks)	Plasma, Chemicon
S104	Tramontina (2009)	10 / 10	34.9 ± 13.8 50.0	pg/µg	0.26 ± 0.10	0.38 ± 0.14	26.2 ± 9.5 (-20.3)	Lithium, valproate, various antipsychotics (8 weeks)	Serum, Chemicon

S106	Wang (2011)	28 / 28	N/A 60.7	pg/mL	1194.9 ± 2214.6	1252.7 ± 1629.7	31.2 ± 5.0 (-26.0)	Lithium and Quetiapine (8 weeks)	Plasma, N/A
S114	Ye (2010)	58 / 43	38.4 ± 14.6 55.2	ng/mL	20.3 ± 8.1	24.7 ± 10.8	35.4 ± 5.4 (-18.1)	N/A (4 weeks)	Serum, Transhold
S77	Yoshimura (2006)	12 / 12	N/A	ng/mL	26.2 ± 11.0	26.1 ± 11.0	22.0 ± 5.0 (-17.0)	Risperidone (4 weeks)	Plasma, Promega

Abbreviation: TAU = treatment as usual; **ER** = extended release.

C) Mixed Episode

Ref	Author (Year)	Sample Size (Baseline/ Final)	Age (mean + SD) % Females	BDNF Unit	BDNF (mean + SD)		Baseline YMRS Scores (Change)	Intervention	Manufacturer of the Test
					Baseline	Post-Treatment			
S68	Grande (2014)	15 / 15	40.3 ± 10.4<br>75.0	ng/mL	44.2 ± 34.9	36.9 ± 13.0	19.9 ± 8.3<br>(-18.2)	Quetiapine ER or TAU	Millipore

Abbreviation: TAU = treatment as usual; **ER** = extended release.

Table S4. Characteristics of meta-analyses of peripheral BDNF in bipolar disorder.

Mood State	Study Data Sets, N	Cases/Controls, N	Meta-analysis ES (95% CI)	95 % Prediction Interval	P Value[a]	Largest Study ES (95% CI)	I^2, %	Egger's Test *P* Value
**Between-group MA**								
Bipolar depression	15	345 / 722	-0.94 (-1.38 – -0.50)	-2.73 – 0.86	**< 0.001**	-0.77 (-1.23 – -0.31)	89	**0.002**
Euthymia	24	1598 / 1459	0.05 (-0.13 – 0.23)	-0.79 – 0.90	0.569	-0.24 (-0.44 – -3.95)	81	0.632
Mania	19	605 / 792	-0.56 (-0.99 – -0.13)	-2.53 – 1.40	**0.010**	0.06 (-0.19 – 0.32)	92	**0.038**
Serum	11	255 / 451	-1.01 (-1.50 – -0.51)	-2.85 – 0.84	**< 0.001**	-0.67 (-1.06 – -0.28)	88	**0.040**
Plasma	6	206 / 188	-0.03 (-0.98 – 0.92)	-3.51 – 3.45	0.951	1.06 (0.66 – 1.47)	95	0.527
Plasma (without Barbosa)	4	124 / 92	-0.72 (-1.27 – -0.17)	-3.06 – 1.61	**0.010**	-0.80 (-1.24 – -0.36)	70	0.885
**Within-group MA**								
Bipolar depression	8	184	0.05 (-0.28 – 0.38)	-1.02 – 1.13	0.754	-0.09 (-0.74 – -0.36)	77	0.423
Mania	13	556	0.26 (0.09 – 0.44)	-0.32 – 0.84	**0.003**	0.29 (-0.17 – 0.19)	69	0.369

Abbreviations: CI = confidence interval; **ES** = effect size; **MA** = meta-analysis

[a] *P* value in test for overall effect (z).

Table S5. Assessment of bias across meta-analyses of peripheral BDNF in bipolar disorder.

Mood State	Significance threshold reached	Observed Positive Data Sets, N	Expected positive data sets[a]		Estimate of heterogeneity[c]	Small-study effects rule[d]	Prediction interval rule[e]
			N	*P* value[b]			
**Between-group meta-analysis**							
Bipolar depression	**< 0.01**	9	11.0	0.251	Very large	**Yes**	**Yes**
Euthymia	> 0.05	10	5.8	**0.048**	Very large	No	**Yes**
Mania	**< 0.01**	12	1.1	**< 0.001**	Very large	**Yes**	**Yes**
Serum	**< 0.01**	7	7.1	0.975	Very large	**Yes**	**Yes**
Plasma	> 0.05	5	5.5	0.447	Very large	No	**Yes**
Plasma (without Barbosa)	**> 0.01 but < 0.05**	3	2.9	0.896	Large	No	**Yes**
**Within-group meta-analysis**							
Bipolar depression	> 0.05	2	0.5	**0.042**	Very large	No	**Yes**
Mania	**< 0.01**	6	4.6	0.431	Large	No	**Yes**

[a] Expected is the sum of the power of all studies to detect a difference equal to the effect size of the largest study with α = 5%.

[b] *P* value in χ^2-based excess significance test.

[c] Large: 50% ≤ I^2 < 75%; Very Large: I^2 ≥ 75%

[d] *P* < 0.1 in Egger's test of publication bias and effect size of the largest study more conservative than the effect size of the meta-analysis

[e] 95% prediction interval includes the null value (zero)

References

S1. Chatterjee B, Sagar R, Vivekanandan S, Sahu A. Correlation of serum BDNF level with clinical symptom domains in bipolar disorder-I. *Bipolar disorders* 2014; **16**: 66.

S2. Chatterjee B, Sahu A, Sagar R, Vivekanandan S. Association of serum BDNF with verbal and visual memory deficit in subjects with bipolar disorder I. *Bipolar disorders* 2014; **16**: 81-82.

S3. Chen B, Dowlatshahi D, MacQueen GM, Wang JF, Young LT. Increased hippocampal BDNF immunoreactivity in subjects treated with antidepressant medication. *Biological psychiatry* 2001; **50**(4)**:** 260-265.

S4. Chen SL, Lee SY, Chang YH, Chen PS, Wang TY, Lu RB. Therapeutic effects of add-on low-dose dextromethorphan plus valproic acid in bipolar disorder. *Journal of Neurochemistry* 2014; **130**: 60-61.

S5. Chou YH, Wang SJ, Lirng JF. Impaired cognition in bipolar i disorder: Searching for a biological substrate. *International Journal of Neuropsychopharmacology* 2012; **15**: 80.

S6. da Graca Cantarelli M, Nardin P, Buffon A, Eidt MC, Antonio Godoy L, Fernandes BS et al. Serum triglycerides, but not cholesterol or leptin, are decreased in suicide attempters with mood disorders. *Journal of affective disorders* 2014; **172C**: 403-409.

S7. da Silva PV. Peripheral biomarkers in bipolar disorder: a population-based study in young adults. *Arquivos de Neuro-Psiquiatria* 2012; **70**: 79-79.

S8. Del Debbio A, Bianchi C, Roncaglia I, Mariotti A, Dell'Osso MC, Mannari C et al. BDNF in plasma following Electroconvulsive therapy: A pilot study in depressed patients. *European Neuropsychopharmacology* 2008; **18**(S4): S301-S302.

S9. Dell'Osso L, Piccinni A, Carlini M, Veltri A, Baroni S, Marazziti D. BDNF plasma levels in bipolar and unipolar depression. *European Psychiatry* 2011; 26(suppl 1): 903.

S10. Duffy A, Horrocks J, Doucette S, Keown-Stoneman C, Grof P, Andreazza A et al. Immunological and neurotrophic markers of risk status and illness development in high-risk youth: Understanding the neurobiological underpinnings of bipolar disorder. *International Journal of Bipolar disorders* 2014; **2**(1): 1-9.

S11. Dunham JS, Deakin JF, Miyajima F, Payton A, Toro CT. Expression of hippocampal brain-derived neurotrophic factor and its receptors in Stanley consortium brains. *Journal of psychiatric research* 2009; **43**(14): 1175-1184.

S12. Ferensztajn E, Skibinska M, Kaczmarek M, Losy J, Rybakowski JK. Mood disorders in the offspring of lithium treated bipolar patients-clinical and neurobiological correlates. *European Neuropsychopharmacology* 2014; **24**: S421-S422.

S13. Ferensztajn E, Skibinska M, Kaczmarek M, Losy J, Rybakowski JK. Neurobiological and temperamental assessment in the offspring of bipolar patients with different degrees of lithium response *European Neuropsychopharmacology* 2014; **24**: S55-S56.

S14. Fernandes BS, Gomes FA, Fries G, Stertz L, Cereser KM, Pessoa C et al. Brain-derived neurotrophic factor as a possible biomarker of bipolar disorder activity. *Bipolar disorders* 2010; **12**: 19.

S15. Filus J, Rybakowski J. Serum BDNF levels and intensity of depressive symptoms. [Polish] Stezenie BDNF w surowicy a nasilenie objawow depresji. *Neuropsychiatria i Neuropsychologia* 2010; **5**(3-4): 155-162.

S16. Gama CS, Andreazza AC, Kunz M, Berk M, Belmonte-de-Abreu PS, Kapczinski F. Serum levels of brain-derived neurotrophic factor in patients with schizophrenia and bipolar disorder. *Neuroscience letters* 2007; **420**(1): 45-48.

S17. Goldstein BI, Collinger KA, Lotrich F, Marsland AL, Gill MK, Axelson DA et al. Preliminary findings regarding proinflammatory markers and brain-derived neurotrophic factor among adolescents with bipolar spectrum disorders. *Journal of Child and Adolescent Psychopharmacology* 2011; **21**(5): 479-484.

S18. Gomes FA, Fernandes BS, Gama CS, Magalhaes PV, Walz JC, Kapczinski F. Correlation between serum brain-derived neurotrophic factor and neurotrophin-3 levels in bipolar disorder. *Bipolar disorders* 2010; **12**: 22.

S19. Grande I, Chendo I, Frey BN, Cereser KM, Fries GR, Stertz L et al. A longitudinal assessment of serum Brain Derived Neurotrophic Factor levels in bipolar patients. *Bipolar disorders* 2011; **13**: 51.

S20. Grande I, Chendo I, Frey BN, Cereser KM, Stertz L, Colpo G et al. Brain-derived neurotrophic factor as a biomarker of clinical response in bipolar disorder: 16 week follow-up study. *European Psychiatry* 2012; 27: 1.

S21. Grande I, Magalhaes P, Chendo I, Sterzt L, Fries G, Cereser K et al. The predictive role of Val66Met polymorphism on levels of BDNF along treatment of medication-free patients with bipolar disorder. *European Neuropsychopharmacology* 2013; **23**: S367-S368.

S22. Grande I, Magalhaes PV, Chendo I, Stertz L, Fries GR, Gama CS et al. Role of biomarkers in the staging of bipolar disorder: Inflammatory factors and oxidative stress. *Bipolar disorders* 2012; **14**: 79.

S23. Grande I, Magalhaes PV, Chendo I, Stertz L, Panizutti B, Colpo GD et al. Staging bipolar disorder: clinical, biochemical, and functional correlates. *Acta Psychiatrica Scandinavica* 2014; **129**(6): 437-444.

S24. Grande I, Magalhaes PVS, Chendo I, Stertz L, Fries GR, Cereser KM et al. Val66Met polymorphism and serum brain-derived neurotrophic factor in bipolar disorder: An open-label trial. *Acta Psychiatrica Scandinavica* 2014; **129**(5): 393-400.

S25. Gronli O, Stensland GO, Wynn R, Olstad R. Neurotrophic factors in serum following ECT: a pilot study. *The World Journal of Biological Psychiatry* 2009; **10**(4): 295-301.

S26. Hashimoto K. BDNF and proBDNF as biomarkers for bipolar disorder *The British journal of psychiatry : the journal of mental science* 2014; **205**(5): 410.

S27. Hashimoto K. Brain-derived neurotrophic factor (BDNF) and its precursor proBDNF as diagnostic biomarkers for major depressive disorder and bipolar disorder. *European Archives of Psychiatry and Clinical Neuroscience* 2015; **265**(1): 83-4.

S28. Kapczinski F, Fernandes BS, Kauer-Sant'Anna M, Gama CS, Yatham LN, Berk M. The concept of staging in bipolar disorder: The role of BDNF and TNF-alpha as biomarkers. *Acta Neuropsychiatrica* 2009; **21**(6): 272-274.

S29. Kapczinski F, Frey BN, Andreazza AC, Kauer-Sant'Anna M, Cunha ABM, Post RM. Increased oxidative stress as a mechanism for decreased BDNF levels in acute manic episodes. *Revista Brasileira de Psiquiatria* 2008; **30**(3): 243-245.

S30. Kauer-Sant'Anna M. Neuroplasticity and early adverse experiences in women with bipolar disorder. *Bipolar disorders* 2010; **12**: 30.

S31. Kauer-Sant'Anna M, Tramotina J, Andreaza AC, Cereser K, da Costa S, Santin A et al. Traumatic life events in bipolar disorder: Impact on BDNF levels and psychopathology. *Bipolar disorders* 2007; **9**(Suppl1): 128-135.

S32. Knorr U, Sondergaard MHG, Koefoed P, Jorgensen A, Faurholt-Jepsen M, Vinberg M et al. Increased whole blood brain-derived neurotrophic factor in healthy individuals with a family history of affective disorder: A case-control study. *Bipolar disorders* 2012; **14**: 94.

S33. Kunz M, Andreazza AC, Bond DJ, Kauer-Sant'Anna M, Yatham LN. Brain-derived neurotrophic factor and inflammatory markers as predictors of outcome in bipolar disorder: Prospective data from the Systematic Treatment Optimization Program for Early Mania (STOP-EM). *Bipolar disorders* 2010; **12**: 33.

S34. Lahera G, Bayon C, Bravo-Ortiz MF, Rodriguez-Vega B, Barbeito S, Saenz M et al. Mindfulness-based cognitive therapy versus psychoeducational intervention in bipolar outpatients with sub-threshold depressive symptoms: A randomized controlled trial. *BMC Psychiatry* 2014; **14:** 215.

S35. Lee SY, Chen SL, Chang YH, Chen PS, Huang SY, Tzeng NS et al. Correlation of plasma brain-derived neurotrophic factor and metabolic profiles in drug-naive patients with bipolar II disorder after a twelve-week pharmacological intervention. *Acta Psychiatrica Scandinavica* 2015; **131**(2): 120-8.

S36. Licinio J, Wong M. Brain-derived neurotrophic factor (BDNF) in stress and affective disorders. *Molecular psychiatry* 2002; **7**(6): 519.

S37. Licinio J, Wong M. Brain-derived neurotrophic factor in depression: A male problem? *Molecular psychiatry* 2010; **15**(3): 227.

S38. Machado-Vieira R, Yuan P, Brutsche N, DiazGranados N, Luckenbaugh D, Manji HK et al. Brain derived neurotrophic factor and initial antidepressant response to an NMDA antagonist. *Bipolar disorders* 2009; **11**(S1): 58-59.

S39. Mesman E, Hillegers MH, Ambree O, Arolt V, Nolen WA, Drexhage HA. Monocyte activation, brain-derived neurotrophic factor (BDNF), and S100B in bipolar offspring: a follow-up study from adolescence into adulthood. *Bipolar disorders* 2015; **17**(1): 39-49.

S40. Moreira L, Neves FS, Romano-Silva MA, Malloy-Diniz LF, Correa H. BDNF and episodic memory in patients with bipolar disorder. *Revista Brasileira de Psiquiatria* 2011; **33**(1): 96-97.

S41. Munkholm K, Pedersen BK, Kessing LV, Vinberg M. Elevated levels of plasma brain derived neurotrophic factor in rapid cycling bipolar disorder patients. *Psychoneuroendocrinology* 2014; **47**: 199-211.

S42. Nair A, Vaidya VA. Cyclic AMP response element binding protein and brain-derived neurotrophic factor: Molecules that modulate our mood? *Journal of Biosciences* 2006; **31**(3): 423-434.

S43. Permoda-Osip A, Adamski R, Bartkowska-Sniatkowska A, Chlopocka-Wozniak M, Skibinska M, Rybakowski JK. Efficacy of single ketamine infusion in bipolar depression: Relationship with serum BDNF. *European Neuropsychopharmacology* 2011; **21**: S428-S429.

S44. Piccinni A, Del Debbio A, Medda P, Bianchi C, Roncaglia I, Veltri A et al. Plasma Brain-Derived Neurotrophic Factor in treatment-resistant depressed patients receiving electroconvulsive therapy. *European Neuropsychopharmacology* 2009; **19**(5): 349-355.

S45. Post RM. Role of BDNF in bipolar and unipolar disorder: clinical and theoretical implications. *Journal of psychiatric research* 2007; **41**(12): 979-990.

S46. Rakofsky J, Ressler K, Dunlop B. BDNF function as a potential mediator of bipolar disorder and post-traumatic stress disorder comorbidity. *Molecular psychiatry* 2012; **17**(1): 22-35.

S47. Rybakowski JK. Response to Lithium in Bipolar Disorder: Clinical and Genetic Findings. *ACS Chemical Neuroscience* 2014.

S48. Scola G, Andreazza AC. The role of neurotrophins in bipolar disorder. *Progress in Neuro-Psychopharmacology and Biological Psychiatry* 2015; **56**: 122-128.

S49. Sousa RT, van de Bilt MT, Diniz BS, Ladeira RB, Portela LV, Souza DO et al. Lithium increases plasma brain-derived neurotrophic factor in acute bipolar mania: a preliminary 4-week study. *Neuroscience letters* 2011; **494**(1): 54-56.

S50. Suwalska A, Sobieska M, Lojko D, Rybakowski JK. Serum brain derived neurotrophic factor in euthymic bipolar patients on prophylactic lithium therapy. *European Neuropsychopharmacology* 2008; **18**(S4): S373.

S51. Teixeira AL, Barbosa IG, Diniz BS, Kummer A. Circulating levels of brain-derived neurotrophic factor: correlation with mood, cognition and motor function. *Biomarkers in Medicine* 2010; **4**(6): 871-887.

S52. Teixeira AL, Barbosa IG, Huguet RB, Bauer ME. Inflammatory parameters and neurotrophic factors in bipolar disorder: Searching trait and state biomarkers. *Inflammation Research* 2011; **60**: S213-S214.

S53. Terao T. Mania is probably associated with hypoactivity of central brain-derived neurotrophic factor. *Medical Hypotheses* 2008; **70**(6): 1232.

S54. Trajkovska V, Vinberg M, Aznar S, Knudsen GM, Kessing LV. Whole blood BDNF levels in healthy twins discordant for affective disorder: association to life events and neuroticism. *Journal of affective disorders* 2008; **108**(1-2): 165-169.

S55. Tsai SJ. Is mania caused by overactivity of central brain-derived neurotrophic factor? *Medical Hypotheses* 2004; **62**(1): 19-22.

S56. Vasconcelos-Moreno MP, Kunz M, Ferrari P, Passos IC, Bucker J, Burque RK et al. BDNF levels in first-degree relatives of patients with bipolar disorder: Preliminary data. *Bipolar disorders* 2012; **14**: 107.

S57. Videira Dias V, Brissos S, Frey BN, Andreazza AC, Kapczinski F. Brain-derived neurotrophic factor levels and cognitive dysfunction in euthymic bipolar type I patients. *Bipolar disorders* 2009; **11**(S1): 88.

S58. Vinberg M, Trajkovska V, Bennike B, Knorr U, Knudsen GM, Kessing LV. The BDNF Val66Met polymorphism: relation to familiar risk of affective disorder, BDNF levels and salivary cortisol. *Psychoneuroendocrinology* 2009; **34**(9): 1380-1389.

S59. Yalcin Arslan Y, Ceylan D, Ozerdem A, Resmi H, Cimrin D, Tunca Z. Interaction between BDNF (brain derived neurotrophic factor) levels and thyroid hormones in adult bipolar disorder patients. *Bipolar Disorders Conference: 10th International Conference on Bipolar Disorder Miami Beach, FL United States Conference Start* 2013; **15**(pp 106): 106.

S60. Yatham LN, Kapczinski F, Andreazza AC, Trevor Young L, Lam RW, Kauer-Sant'anna M. Accelerated age-related decrease in brain-derived neurotrophic factor levels in bipolar disorder. *International Journal of Neuropsychopharmacology* 2009; **12**(1): 137-139.

S61. Yuan TF. A broken BDNF hypothesis. *Medical Hypotheses* 2008; **71**(2): 322.

S62. Zhang C, Li Z, Fang Y. Brain-derived neurotrophic factor levels predict bipolar disorders in their first depressive episode: Result of a longitudinal study. *Bipolar disorders* 2014; **16**: 54.

S63. Ceylan D, Ozerdem A, Gurz Yalcin SN, Hidirotlu C, Aslan YC, Batci B et al. Can serum BDNF levels be identified as a candidate endophenotype in bipolar disorder? *Bipolar disorders* 2012; **14**: 66.

S64. Cunha AB, Frey BN, Andreazza AC, Goi JD, Rosa AR, Goncalves CA et al. Serum brain-derived neurotrophic factor is decreased in bipolar disorder during depressive and manic episodes. *Neuroscience letters* 2006; **398**(3): 215-219.

S65. de Oliveira GS, Cereser KM, Fernandes BS, Kauer-Sant'Anna M, Fries GR, Stertz L et al. Decreased brain-derived neurotrophic factor in medicated and drug-free bipolar patients. *Journal of psychiatric research* 2009; **43**(14): 1171-1174.

S66. Dell'Osso L, Bianchi C, Del Debbio A, Roncaglia I, Veltri A, Carlini M et al. Plasma brain-derived neurotrophic factor in bipolar and unipolar depression. *Italian Journal of Psychopathology* 2010; **16**(2): 138-143.

S67. Fernandes BS, Gama CS, Kauer-Sant'Anna M, Lobato MI, Belmonte-de-Abreu P, Kapczinski F. Serum brain-derived neurotrophic factor in bipolar and unipolar depression: a potential adjunctive tool for differential diagnosis. *Journal of psychiatric research* 2009; **43**(15): 1200-1204.

S68. Grande I, Magalhaes PV, Chendo I, Stertz L, Fries GR, Cereser KM et al. Val66Met polymorphism and serum brain-derived neurotrophic factor in bipolar disorder: an open-label trial. *Acta Psychiatrica Scandinavica* 2014; **129**(5): 393-400.

S69. Kapczinski F, Dal-Pizzol F, Teixeira AL, Magalhaes PV, Kauer-Sant'Anna M, Klamt F et al. Peripheral biomarkers and illness activity in bipolar disorder. *Journal of psychiatric research* 2011; **45**(2): 156-161.

S70. Li Z, Zhang C, Fan J, Yuan C, Huang J, Chen J et al. Brain-derived neurotrophic factor levels and bipolar disorder in patients in their first depressive episode: 3-year prospective longitudinal study. *The British journal of psychiatry : the journal of mental science* 2014; **205**(1): 29-35.

S71. Mackin P, Gallagher P, Watson S, Young A, Ferrier IN. Changes in brain-derived neurotrophic factor following treatment with mifepristone in bipolar disorder and schizophrenia. *Australian and New Zealand Journal of Psychiatry* 2007; **41**(4): 321-326.

S72. Magalhaes PV, Jansen K, Pinheiro RT, Fries GR, Teixeira AL, Da Silva RA et al. A nested population-based case-control study on peripheral inflammation markers and brainderived neurotrophic factor in early-stage mood disorders. *Bipolar disorders* 2012; **14**: 48.

S73. Panizzutti BS, Wollenhaupt-Aguiar B, Pfaffenseller B, Spanemberg L, Caldieraro MA, Vares EA et al. Evaluation of peripheral biomarkers in bipolar and unipolar depression. *European Neuropsychopharmacology* 2013; **23**: S361-S362.

S74. Rabie MA, Mohsen M, Ibrahim M, El-Sawy Mahmoud R. Serum level of brain derived neurotrophic factor (BDNF) among patients with bipolar disorder. *Journal of affective disorders* 2014; **162**: 67-72.

S75. Su SC, Sun MT, Wen MJ, Lin CJ, Chen YC, Hung YJ. Brain-derived neurotrophic factor, adiponectin, and proinflammatory markers in various subtypes of depression in young men. *International journal of psychiatry in medicine* 2011; **42**(3): 211-226.

S76. Tunca Z, Ozerdem A, Ceylan D, Yalcin Y, Can G, Resmi H et al. Alterations in BDNF (brain derived neurotrophic factor) and GDNF (glial cell line-derived neurotrophic factor) serum levels in bipolar disorder: The role of lithium. *Journal of affective disorders* 2014; **166**: 193-200.

S77. Yoshimura R, Nakano Y, Hori H, Ikenouchi A, Ueda N, Nakamura J. Effect of risperidone on plasma catecholamine metabolites and brain-derived neurotrophic factor in patients with bipolar disorders. *Human psychopharmacology* 2006; **21**(7): 433-438.

S78. Andreazza AC, Rajji TK, Gildengers A, Soares AT, Lafer B, Young LT et al. Decrease brain-derived neurotrophic factor (BDNF) in older patients with bipolar disorder. *Biological psychiatry* 2014; **75**(suppl): 41S.

S79. Barbosa IG, Huguet RB, Mendonca VA, Neves FS, Reis HJ, Bauer ME et al. Increased plasma levels of brain-derived neurotrophic factor in patients with long-term bipolar disorder. *Neuroscience letters* 2010; **475**(2): 95-98.

S80. Barbosa IG, Rocha NP, Huguet RB, Ferreira RA, Salgado JV, Carvalho LA et al. Executive dysfunction in euthymic bipolar disorder patients and its association with plasma biomarkers. *Journal of affective disorders* 2012; **137**(1-3): 151-155.

S81. Barbosa IG, Rocha NP, de Miranda AS, Huguet RB, Bauer ME, Reis HJ et al. Increased BDNF levels in long-term bipolar disorder patients. *Revista Brasileira de Psiquiatria* 2013; **35**(1): 67-69.

S82. Chen SL, Lee SY, Chang YH, Chen SH, Chu CH, Wang TY et al. The BDNF Val66Met polymorphism and plasma brain-derived neurotrophic factor levels in Han Chinese patients with bipolar disorder and schizophrenia. *Progress in Neuro-Psychopharmacology and Biological Psychiatry* 2014; **51**: 99-104.

S83. Chou YH, Wang SJ, Lirng JF, Lin CL, Yang KC, Chen CK et al. Impaired cognition in bipolar I disorder: the roles of the serotonin transporter and brain-derived neurotrophic factor. *Journal of affective disorders* 2012; **143**(1-3): 131-137.

S84. Dias VV, Brissos S, Frey BN, Andreazza AC, Cardoso C, Kapczinski F. Cognitive function and serum levels of brain-derived neurotrophic factor in patients with bipolar disorder. *Bipolar disorders* 2009; **11**(6): 663-671.

S85. Hsieh WC, Jou YT, Lin JL, Wang SJ, Chou YH. The effect of cortisol and bdnf on serotonin transporter in bipolar I disorder. *Bipolar disorders* 2014; **16**: 89.

S86. Kauer-Sant'Anna M, Kapczinski F, Andreazza AC, Bond DJ, Lam RW, Young L et al. Brain-derived neurotrophic factor and inflammatory markers in patients with early- vs. late-stage bipolar disorder. *International Journal of Neuropsychopharmacology* 2009; **12**(4): 447-458.

S87. Kenna HA, Reynolds-May M, Stepanenko A, Ketter TA, Hallmayer J, Rasgon NL. Blood levels of brain derived neurotrophic factor in women with bipolar disorder and healthy control women. *Journal of affective disorders* 2014; **156**: 214-218.

S88. Langan C, Doyle K, Kelly J, Emsell L, Skinner R, McDonald C. Serum BDNF levels in euthymic bipolar disorder: Preliminary results from the galway bipolar study. *Bipolar disorders* 2009; **11**(S1): 56.

S89. Lotrich FE, Butters MA, Aizenstein H, Marron MM, Reynolds CF, 3rd, Gildengers AG. The relationship between interleukin-1 receptor antagonist and cognitive function in older adults with bipolar disorder. *International journal of geriatric psychiatry* 2014; **29**(6)**:** 635-644.

S90. Monteleone P, Serritella C, Martiadis V, Maj M. Decreased levels of serum brain-derived neurotrophic factor in both depressed and euthymic patients with unipolar depression and in euthymic patients with bipolar I and II disorders. *Bipolar disorders* 2008; **10**(1): 95-100.

S91. Panizzutti BS, Gubert C, Schuh AL, Ferrari P, Bristot G, Fries GR *et al.* Increased serum levels of CCL11/eotaxin in late stage bipolar patients. *Biological psychiatry* 2014; **1):** 183S.

S92. Rosa AR, Singh N, Whitaker E, de Brito M, Lewis AM, Vieta E et al. Altered plasma glutathione levels in bipolar disorder indicates higher oxidative stress; a possible risk factor for illness onset despite normal brain-derived neurotrophic factor (BDNF) levels. *Psychological medicine* 2014; **44**: 2409–2418.

S93. Rybakowski JK, Suwalska A. Excellent lithium responders have normal cognitive functions and plasma BDNF levels. *The international journal of neuropsychopharmacology / official scientific journal of the Collegium Internationale Neuropsychopharmacologicum (CINP)* 2010; **13**(5)**:** 617-622.

S94. Sodersten K, Palsson E, Ishima T, Funa K, Landen M, Hashimoto K et al. Abnormality in serum levels of mature brain-derived neurotrophic factor (BDNF) and its precursor proBDNF in mood-stabilized patients with bipolar disorder: a study of two independent cohorts. *Journal of affective disorders* 2014; **160**: 1-9.

S95. Suwalska A, Sobieska M, Rybakowski J. Serum brain-derived neurotrophic factor in euthymic bipolar patients on prophylactic lithium therapy. *Neuropsychobiology* 2010; **62**(4): 229-234.

S96. Tramontina J, Frey B, Andreazza A, Zandona M, Santin A, Kapczinski F. Val66met polymorphism and serum brain-derived neurotrophic factor levels in bipolar disorder. *Molecular psychiatry* 2007; **12**(3)**:** 230-231.

S97. Goka E, Goka S, Aydemir C, Aksaray S, Yalcin ES, Kisa C. BDNF levels and change with treatment in patients with bipolar disorder manik episode. *Bulletin of Clinical Psychopharmacology* 2009; **19**(Suppl. 1): S8-S13 [Turkish]

S98. Huang TL, Hung YY, Lee CT, Chen RF. Serum protein levels of brain-derived neurotrophic factor and tropomyosin-related kinase B in bipolar disorder: effects of mood stabilizers. *Neuropsychobiology* 2012; **65**(2): 65-69.

S99. Karamustafalioglu N, Genc A, Kalelioglu T, Tasdemir A, Umut G, Incir S et al. Plasma BDNFs level initially and post treatment in acute mania: comparison between ECT and atypical antipsychotic treatment and healthy controls. *Journal of Psychopharmacology* 2015.

S100. Kim Y-K, Na K-S, Hwang J-A, Yoon H-K, Lee H-J, Hahn S-W et al. High insulin-like growth factor-1 in patients with bipolar I disorder: A trait marker? *Journal of affective disorders* 2013; **151**(2): 738-743.

S101. Laske C, Stransky E, Eschweiler GW, Wittorf A, Richartz-Salzburger E, Bartels M *et al.* Brain-derived neurotrophic factor (BDNF) in serum of patients with mania, major depression and healthy controls. *Neurology Psychiatry and Brain Research* 2005; **12**(1)**:** 25-28.

S102. Machado-Vieira R, Dietrich MO, Leke R, Cereser VH, Zanatto V, Kapczinski F et al. Decreased plasma brain derived neurotrophic factor levels in unmedicated bipolar patients during manic episode. *Biological psychiatry* 2007; **61**(2): 142-144.

S103. Palomino A, Vallejo-Illarramendi A, Gonzalez-Pinto A, Aldama A, Gonzalez-Gomez C, Mosquera F et al. Decreased levels of plasma BDNF in first-episode schizophrenia and bipolar disorder patients. *Schizophrenia research* 2006; **86**(1-3): 321-322.

S104. Tramontina JF, Andreazza AC, Kauer-Sant'anna M, Stertz L, Goi J, Chiarani F et al. Brain-derived neurotrophic factor serum levels before and after treatment for acute mania. *Neuroscience letters* 2009; **452**(2): 111-113.

S105. Tunca Z, Kıvırcık Akdede B, Özerdem A, Alkın T, Polat S, Ceylan D et al. Diverse glial cell line-derived neurotrophic factor (GDNF) support between mania and schizophrenia: a comparative study in four major psychiatric disorders. *European Psychiatry* 2015; **30**(2): 198–204.

S106. Wang ZW, Li ZZ, Lin ZG, Wu ZG, Yuan CM, Hong W et al. Changes of plasma brain-derived neurotrophic factor in patients with bipolar disorder type I. *Journal of Shanghai Jiaotong University (Medical Science)* 2011; **31**(11): 1532-1535.

S107. Piccinni A, Veltri A, Costanzo D, Vanelli F, Franceschini C, Moroni I et al. Decreased plasma levels of brain-derived neurotrophic factor (BDNF) during mixed episodes of bipolar disorder. *Journal of affective disorders* 2015; **171**: 167-170.

S108. Grande I, Kapczinski F, Stertz L, Colpo GD, Kunz M, Cereser KM et al. Peripheral brain-derived neurotrophic factor changes along treatment with extended release quetiapine during acute mood episodes: An open-label trial in drug-free patients with bipolar disorder. *Journal of psychiatric research* 2012; **46**(11): 1511-1514.

S109. Lee SY, Chen SL, Chang YH, Chen PS, Huang SY, Tzeng NS et al. Correlation of plasma brain-derived neurotrophic factor and metabolic profiles in drug-naive patients with bipolar II disorder after a twelve-week pharmacological intervention. *Acta Psychiatrica Scandinavica* 2015; **131**(2): 120-8.

S110. Rybakowski JK, Permoda-Osip A, Skibinska M, Adamski R, Bartkowska-Sniatkowska A. Single ketamine infusion in bipolar depression resistant to antidepressants: are neurotrophins involved? *Human psychopharmacology* 2013; **28**(1): 87-90.

S111. Yoshimura R, Ikenouchi-Sugita A, Hori H, Umene-Nakano W, Katsuki A, Hayashi K *et al.* Adding a low dose atypical antipsychotic drug to an antidepressant induced a rapid increase of plasma brain-derived neurotrophic factor levels in patients with treatment-resistant depression. *Progress in neuro-psychopharmacology & biological psychiatry* 2010; **34**(2**)**: 308-312.

S112. de Sousa RT, van de Bilt MT, Diniz BS, Ladeira RB, Portela LV, Souza DO et al. Lithium increases plasma brain-derived neurotrophic factor in acute bipolar mania: a preliminary 4-week study. *Neuroscience letters* 2011; **494**(1): 54-56.

S113. Kurita M, Nishino S, Numata Y, Okubo Y, Sato T. The noradrenaline metabolite MHPG is a candidate biomarker from the manic to the remission state in bipolar disorder I: a clinical naturalistic study. *PloS one* 2014; **9**(6)**:** e100634.

S114. Ye C, Xu Y, Hu H. The serum concentration of brain-derived neurotrophic factor (BDNF) increased after four weeks treatment in acute mania patients. *International Journal of Neuropsychopharmacology* 2010; **13**: 133.
